# Carriage of rare *APOB* variants predisposes to severe steatotic liver disease and hepatocellular carcinoma

**DOI:** 10.1172/JCI201762

**Published:** 2026-02-10

**Authors:** Matteo Mureddu, Serena Pelusi, Oveis Jamialahmadi, Marijana Vujkovic, Lorenzo Miano, Hadi Eidgah Torghabehei, Luisa Ronzoni, Francesco Malvestiti, Marco Saracino, Giulia Periti, Vittoria Moretti, Craig C. Teerlink, Julie A. Lynch, Philip S. Tsao, Josephine P. Johnson, Vincenzo La Mura, Robertino Dilena, Saleh A. Alqahtani, Alessandro Cherubini, EPIDEMIC Study Investigators, Million Veteran Program, Francesco Paolo Russo, Roberta D’Ambrosio, Mirella Fraquelli, Salvatore Petta, Luca Miele, Umberto Vespasiani-Gentilucci, Elisabetta Bugianesi, Rosellina M. Mancina, Paolo Parini, Daniele Prati, Kyong-Mi Chang, Carolin V. Schneider, Stefano Romeo, Luca VC Valenti

**Affiliations:** 1 Università degli Studi di Milano, Department of Pathophysiology and Transplantation, Milan, Italy.; 2 Precision Medicine and Biological Resource Center, Fondazione IRCCS Ca’ Granda Ospedale Maggiore Policlinico Milano, Milan, Italy.; 3 Department of Molecular and Clinical Medicine, Institute of Medicine, Sahlgrenska Academy, Wallenberg Laboratory, University of Gothenburg, Gothenburg, Sweden.; 4 Corporal Michael J. Crescenz VA Medical Center, Philadelphia, Pennsylvania, USA.; 5 University of Pennsylvania Perelman School of Medicine, Philadelphia, Pennsylvania, USA.; 6 Scientific Direction, Fondazione IRCCS Ca’ Granda Ospedale Maggiore Policlinico Milano, Milan, Italy.; 7 Department of Internal Medicine and Therapeutics, University of Pavia, Pavia, Italy.; 8 VA Salt Lake City and; 9 University of Utah School of Medicine, Salt Lake City, Utah, USA.; 10 VA Palo Alto Health Care System and Cardiovascular Medicine, Stanford University School of Medicine, Palo Alto, California, USA.; 11 Fondazione IRCCS Ca’ Granda Ospedale Maggiore Policlinico, Angelo Bianchi Bonomi Hemophilia and Thrombosis Center, Milan, Italy.; 12 Neurology, Fondazione IRCCS Ca’ Granda Ospedale Maggiore Policlinico Milano, Milan, Italy.; 13 King Faisal Specialist Hospital and Research Centre, Riad, Saudi Arabia.; 14 The EPIDEMIC Study Investigators is detailed in Supplemental Acknowledgments.; 15 The Million Veteran Program is detailed in Supplemental Acknowledgments.; 16 Hepatology, University of Padua, Padua, Italy.; 17 Gastroenterology and Hepatology, and; 18 Gastroenterology and Endoscopy, Fondazione IRCCS Ca’ Granda Ospedale Maggiore Policlinico Milano, Milan, Italy.; 19 Gastroenterology and Hepatology, PROMISE, Università di Palermo, Palermo, Italy.; 20 Department of Internal Medicine, Fondazione Policlinico A. Gemelli, Università Cattolica di Roma, Rome, Italy.; 21 Clinical Medicine and Hepatology Unit, Department of Internal Medicine and Geriatrics, Campus Bio-Medico University, Rome, Italy.; 22 Department of Medical Sciences, Division of Gastro-Hepatology, A.O. Città della Salute e della Scienza di Torino, Università di Torino, Turin, Italy.; 23 Department of Life Science, Health, and Health Professions, Link Campus University, Rome, Italy.; 24 Research Unit of Clinical Medicine and Hepatology, Department of Medicine and Surgery, Università Campus Bio-Medico di Roma, Rome, Italy.; 25 Medical Unit Endocrinology, Theme Inflammation and ageing, Karolinska University Hospital, Huddinge, Sweden.; 26 Gastroenterology, Metabolic Diseases, and Intensive Care, Department of Medicine III, University Hospital RWTH Aachen, Aachen, Germany.; 27 Department of Medicine (H7), CeRM, Karolinska Institute, Huddinge, Sweden.; 28 Department of Cardiology, Sahlgrenska University Hospital, Gothenburg, Sweden.; 29 Clinical Nutrition Unit, Department of Medical and Surgical Science, University Magna Graecia, Catanzaro, Italy.

**Keywords:** Genetics, Hepatology, Genetic risk factors, Lipoproteins, Liver cancer

## Abstract

**BACKGROUND:**

Metabolic dysfunction–associated steatotic liver disease (MASLD) has a substantial inherited component. Rare variants in apolipoprotein B gene (*APOB*) have been implicated in susceptibility to liver steatosis, but their role in disease progression and outcomes is unclear.

**METHODS:**

We investigated *APOB* rare variants in a case-control cohort of people with advanced MASLD versus healthy controls (*n* = 510 and 261, respectively), a family-based study (*n* = 43 and literature meta-analysis), the Million Veteran Program (MVP) cohort (*n* = 94,885), and the UK Biobank (UKBB) (*n* = 417,657).

**RESULTS:**

In the clinical cohort, *APOB* variants were enriched in people with advanced MASLD (OR 13*.*8, 95% CI: 2*.*7–70*.*7, *P* = 0*.*002) and associated with lower circulating lipids, but higher MASLD activity and fibrosis (*P* < 0*.*05). In the family study, *APOB* variants segregated with hepatic steatosis and fibrosis (*P* < 0*.*05). Cross-ancestry meta-analysis of the study cohorts yielded pooled ORs for cirrhosis and hepatocellular carcinoma (HCC) of 1*.*82, 95% CI: 1*.*33–2*.*49 and 3*.*53, 95% CI: 2*.*09–5*.*98, respectively. Variants affecting specifically ApoB100 had a 3-fold greater effect on hepatic lipid metabolism compared with those impairing also ApoB48 and were specifically protective against coronary artery disease (*P* < 0*.*05). The variants affected cirrhosis risk similarly, but ApoB48/100 had a larger effect on HCC (*P* < 0*.*05).

**CONCLUSIONS:**

Rare *APOB* variants predispose individuals to advanced MASLD and HCC, with distinct contributions from disrupted VLDL and chylomicrons secretion. These findings highlight the interplay between hepatic and intestinal lipid handling, suggesting that *APOB* genotyping may enhance MASLD risk stratification and patient identification.

**FUNDING:**

European Union, Italian Ministry of Health, Swedish Research Council, Veterans Health Administration, NIH.

## Abstract 2

In this study we show how different types of Apolipoprotein B (APOB) mutations affect the risk of developing cirrhosis and liver cancer

## Graphical abstract

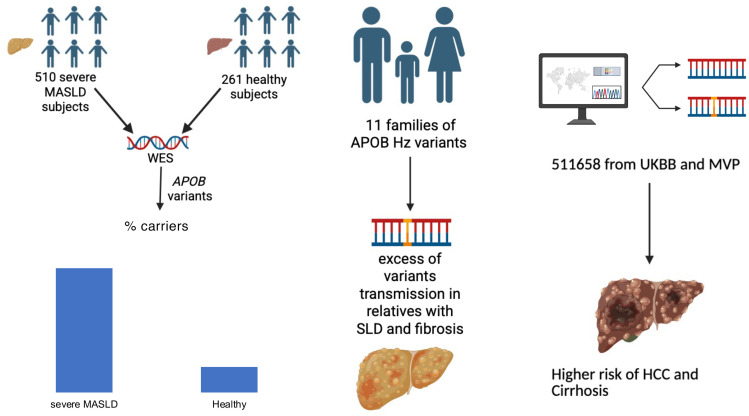

## Introduction

Driven by the global epidemics of obesity and type 2 diabetes (T2D), metabolic dysfunction–associated steatotic liver disease (MASLD) is now the primary cause of liver disease, affecting 1 in 3 individuals worldwide (1). MASLD can progress to steatohepatitis (metabolic dysfunction–associated steatohepatitis [MASH]), which promotes hepatic fibrogenesis and is emerging as a major cause of cirrhosis and hepatocellular carcinoma (HCC) (2). At the cellular level, MASLD is defined by excessive accumulation of triglycerides within intracellular lipid droplets (LDs), reflecting a fundamental disturbance of lipid metabolism (3). Despite its high prevalence and clinical impact, noninvasive tools for accurate risk stratification remain a major unmet clinical need (4).

Heritability accounts for approximately 50% of MASLD interindividual variability, and GWASs have identified the major common genetic contributors to disease susceptibility (5). The genetic architecture of common variants indicates a largely linear relationship between genetic effects on hepatic fat accumulation and the risk of downstream outcomes, including cirrhosis and HCC (6–8). We previously demonstrated that the main genetic determinants of steatohepatitis dissociate hepatic and cardiometabolic complications of MASLD (9). Subsequent work highlighted that some genetic variants drive liver-specific outcomes, whereas others influence cardiometabolic complications (10). Nonetheless, a large portion of the variability of severe MASLD remains unexplained (11).

Apolipoprotein B (APOB) codes for the major apolipoprotein responsible for triglyceride secretion in the liver and small intestine. Tissue-specific posttranscriptional mRNA editing (C->U deamination) introduces a premature stop codon resulting in the synthesis of 2 distinct isoforms: the full-length ApoB100 (~500 kDa) in hepatocytes, which is required for very low-density lipoprotein (VLDL) secretion, and the truncated ApoB48 (~240 kDa) in enterocytes, which is required for chylomicron formation (12). Heterozygous APOB loss-of-function (LoF) variants are associated with reduced VLDL secretion and protection against atherosclerosis (13). Furthermore, homozygous familial hypobetalipoproteinemia, caused by APOB LoF mutations, leads to very low levels of ApoB-containing lipoproteins, lipid malabsorption, and an elevated risk of cirrhosis (14). Accordingly, heterozygous APOB LoF rare variants anecdotally cosegregated with cirrhosis and HCC in extended family pedigrees (15, 16). The association between APOB LoF variants and liver disease has been corroborated by studies in the population-based UK Biobank (UKBB) cohort (17, 18). However, the contribution of these variants to severe liver disease and HCC in clinical studies has not been systematically examined, especially for their potential to explain individual risk and to cosegregate with liver disease in families (15, 16, 18–20). Additionally, the effect of the mutation location, whether disrupting both ApoB48 and ApoB100 (upstream of amino acid 2154) or only ApoB100 (downstream), has not been investigated.

Here, we aimed to determine how rare *APOB* variants affecting ApoB48 and/or ApoB100 contribute to severe MASLD, defined as the presence of advanced fibrosis or HCC. To this end, we applied a comprehensive approach using clinical and histological familial analysis, and large-scale population-based biobank data.

## Results

### APOB variants are enriched in people with severe MASLD.

**Figure 1:**
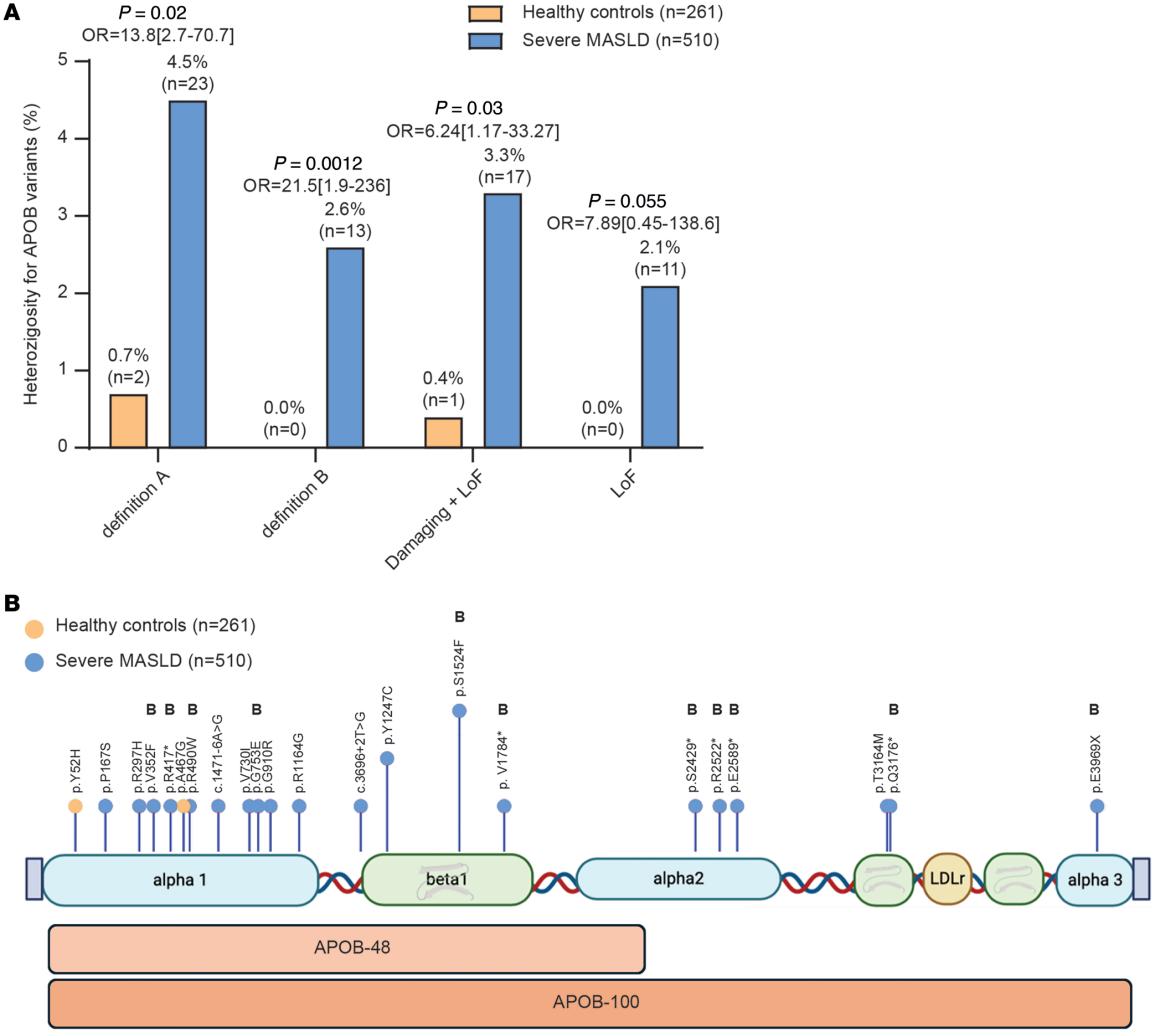
Risk associated with carriage of rare *APOB* variants in a severe MASLD case-control cohort according to definition A, definition B, damaging plus LoF variants, and LoF *APOB* variants. OR, 95% CI, and *P* values were calculated by logistic regression analysis adjusted for age and sex. (**A**) Prevalence of variants in cases versus controls. (**B**) Lollipop graph showing the aminoacidic residues affected by the mutations.

We started by examining a case-control study of people with severe MASLD (*n* = 510 people with MASLD; *n* = 261 controls; Supplemental Table 3; supplemental material available online with this article; https://doi.org/10.1172/JCI201762DS1). Rare variants in *APOB* (definition A) (Supplemental Methods) were enriched more than 6-fold in individuals with MASLD compared with controls (4.5% vs 0.7%, Figure 1A). After adjusting for age and sex, *APOB* variant carriers had an almost 14-fold higher risk for severe MASLD (Figure 1, A and B, and Supplemental Table 4 reporting burden test results; adjusted OR 13.8, 95% CI: 2.7–70.7, *P* = 0.002). In a sensitivity analysis, using the more stringent definition B (Supplemental Methods), this risk increase was even larger (adjusted OR for definition B: 21.5, 95% CI: 1.9–236, *P* = 0.012; Figure 1 and Supplemental Table 4). Similar results were obtained for pure LoF variants (Supplemental Table 5).

Among people at the clinic, *APOB* variant carriers exhibited markedly reduced LDL cholesterol and triglyceride levels alongside modestly elevated HDL cholesterol in plasma (*P* < 0.05; Supplemental Table 3), although they were less frequently on statins (*P* = 0.059), supporting a LoF effect of the variants identified. In people who were not on statins, lower LDL cholesterol levels (71.7 vs. 104.1 mg/dL, *P* = 0.002 for definition A; 48.6 ± 30.0 vs. 103.9 ± 41.6, *P* < 0.0001 for definition B in carriers vs. noncarriers) allowed prediction of the presence of *APOB* variants (area under the receiver operating characteristic [AUROC] 0.73, best threshold 55.4 mg/dL, 50% sensitivity and 42% specificity for definition A; AUROC 0.89, best threshold 70.6 mg/dL, 90% sensitivity and 72% specificity for definition B). *APOB* variant carriers had lower platelet numbers (*P* = 0.006, Supplemental Figure 1A) and a higher Fibrosis-4 index (FIB-4) score (*P* = 0.04, Supplemental Figure 1B), as well as a higher prevalence of advanced liver fibrosis (11 of 11, 100%, vs. 309/499, 61.9%, *P* = 0.03). Among people with available liver biopsies (*n* = 156), *APOB* variant carriers had higher grades of ballooning (*P* = 0.014, Supplemental Table 3).

### Inheritance of APOB variants is associated with the MASLD spectrum within families.

Next, we characterized 11 families (Milan family study, Supplemental Figure 2 and Supplemental Table 2), whose probands had severe MASLD. Overall, we examined 32 first-degree relatives, 12 (37.5%) with steatosis, and 7 (21.8%) with liver fibrosis. Within these pedigrees, *APOB* variants cosegregated with liver steatosis (*P* = 0.0045), severe steatosis (*P* = 0.004), and fibrosis (*P* = 0.034, Table 1). These associations were strengthened by including additional family pedigrees from previous studies in the analyses (Table 1).

### Carriage of APOB variants is associated with cirrhosis and HCC in large population cohorts.

**Figure 2:**
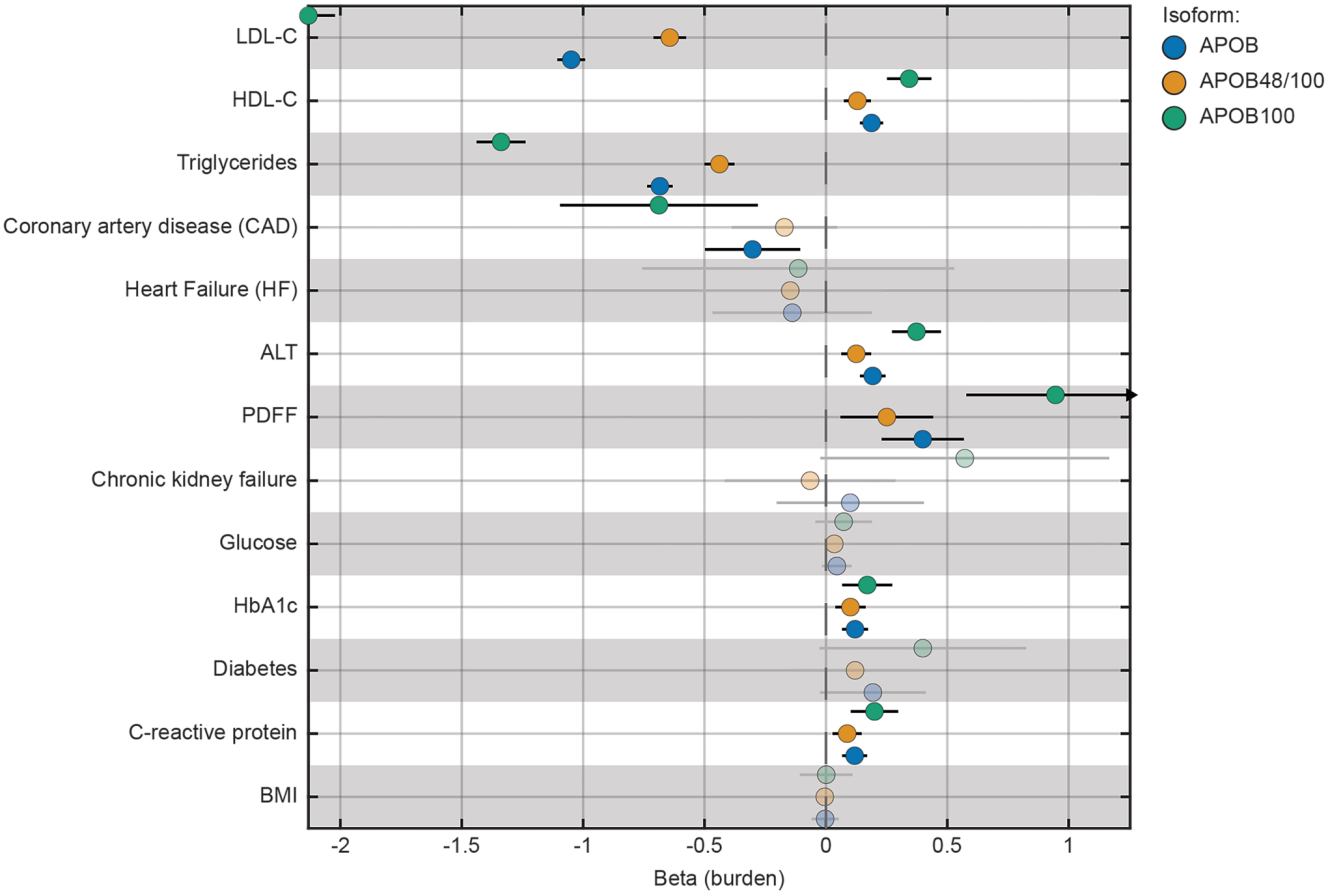
Risk of cardiometabolic and liver outcomes associated with rare pathogenic *APOB* LoF variants in individuals from UKBB (*n* = 417,657). The *x*-axis shows either β (continuous traits) or log Firth’s OR (binary traits) from a burden test, while adjusting for age, sex, age × sex, age^2^ × sex, age^2^, smoking, alcohol consumption, and BMI, with additional adjustments for diabetes and hypertension for CAD. Blue, orange, and green dots denote LoF variants affecting the full *APOB* gene, both ApoB48/100 and ApoB100, and exclusively ApoB100 protein isoforms, respectively. Transparent dots indicate nonsignificant associations.

**Figure 3:**
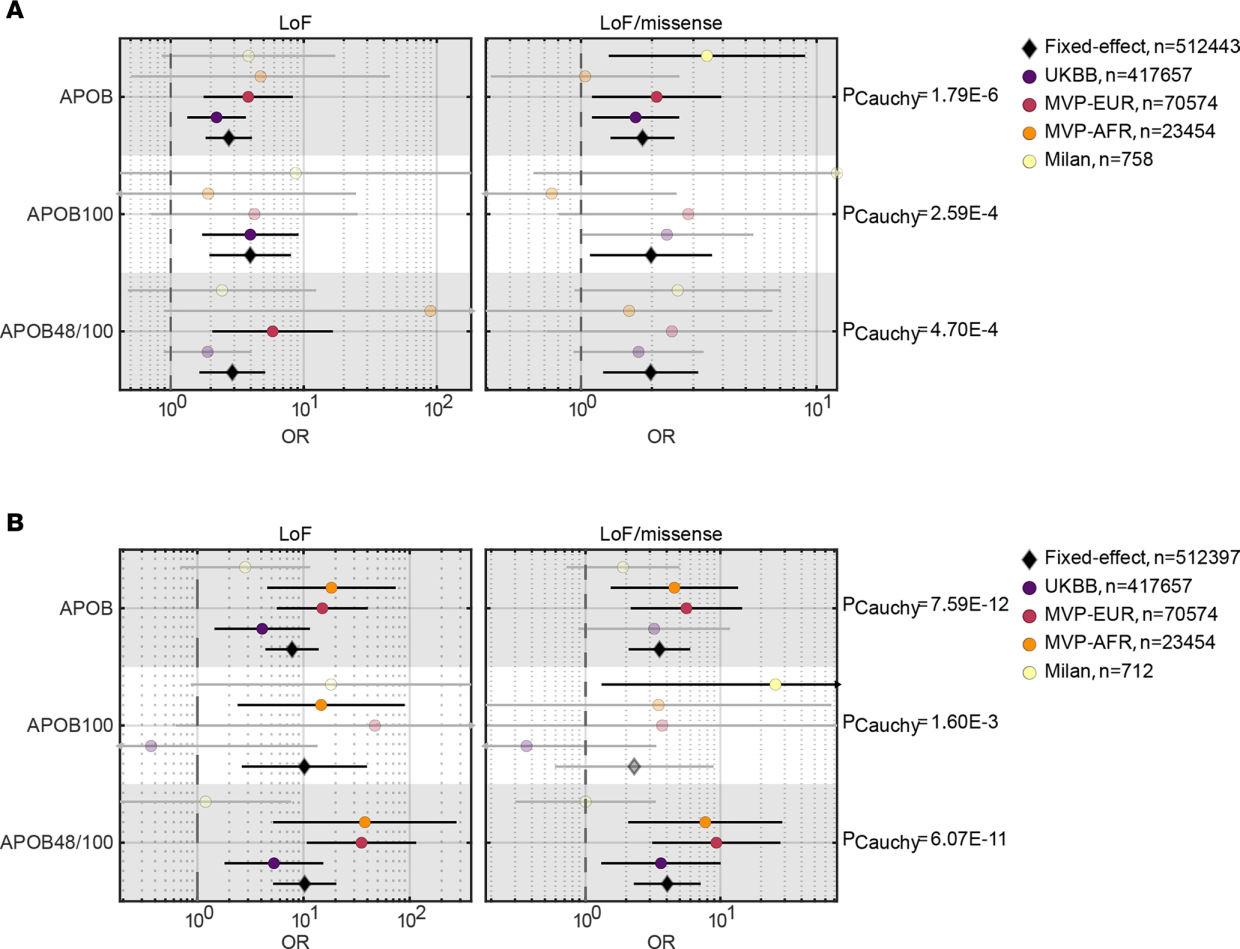
Cross-ancestry meta-analysis of rare *APOB* variants effects on liver outcomes in 3 study cohorts. (**A**) Cirrhosis and (**B**) HCC Firth’s corrected OR for each study is displayed for all variants affecting the *APOB* gene (ApoB), specifically affecting ApoB48/100, and exclusively affecting ApoB100 protein isoforms. Diamond marker shows the fixed-effect meta-analysis of each mask-isoform pair. *P* values for each mask were combined using Cauchy distribution as shown on the right. Transparent dots indicate nonsignificant associations. Milan, Milan Biobank case-control cohort; MVP-EUR, MVP Cohort Biobank, European Americans; MVP-AFR, MVP Cohort Biobank, African Americans.

Next, we sought to replicate our findings in 2 large independent cohorts, namely the Million Veteran Program (MVP) biobank and the UKBB. Considering the large study size and the study design, in these cohorts we focused the analyses on LoF and LoF/deleterious missense variants. Among MVP participants (Supplemental Table 6), carriage of LoF *APOB* variants was associated with an increased risk of cirrhosis in individuals with European ancestry and of HCC in both African and European ancestries (*P* < 0.05 for all). In the whole cohort, *APOB* variants conferred an increased risk of cirrhosis (OR 3.28, *P* = 0.009) and HCC (OR 11.68, *P* = 6.35 × 10^–5^). In the UKBB, rare variants were associated with a higher risk of cirrhosis and HCC (Supplemental Table 7). The effect of rare *APOB* variants on liver and cardiometabolic-related phenotypes in the UKBB is presented in Figure 2 and Supplemental Table 8. As expected, *APOB* carriers had lower LDL cholesterol levels, lower circulating triglyceride levels, and higher HDL cholesterol levels and were protected against coronary artery disease (CAD). Conversely, they had higher hepatic triglyceride content, liver enzymes, and prevalence of chronic liver disease (*P* < 0.05 for all). Interestingly, *APOB* variant carriers had higher C-reactive protein (CRP), glucose, glycated hemoglobin (HbA1c), and prevalence of diabetes (all *P* < 0.05). Finally, we performed a cross-ancestry meta-analysis of liver outcomes using harmonized definition of LoF and LoF/missense in the 3 studies, which revealed that *APOB* carriers had a higher risk of cirrhosis (*P*_Cauchy_ = 1.79 × 10^–6^; Figure 3A) and HCC (*P*_Cauchy_ = 7.59 × 10^–12^, Figure 3B). The pooled OR for cirrhosis was 1.82 (95% CI: 1.33–2.49) for LoF/missense variants and 2.74 (95% CI: 1.83–4.09) for LoF variants. The pooled OR for HCC was 3.53 (95% CI: 2.09–5.98) for LoF/missense variants and 7.79 (95% CI: 4.37–13.92) for LoF variants.

### Differential effect of LoF variants on ApoB isoforms.

Next, we examined the effect of *APOB* variants on liver outcomes based on the amino acidic sequence location where the variants occur and therefore their effect on the 2 ApoB isoforms. Specifically, if the mutation occurred downstream of amino acid 2154, the LoF affected only ApoB100. Conversely, if the mutation occurred upstream of this position, both isoforms were affected, namely, ApoB48/100 (Figure 1B and Figure 3). The results are reported in detail in Supplemental Tables 6–8 and in Figure 3. While both the LoF and LoF/missense mutations in ApoB48/100 and ApoB100 isoforms showed a similar level of significance in the association with cirrhosis (*P*_Cauchy_ = 4.70 × 10^–4^ for ApoB48/100 and *P*_Cauchy_ = 2.59 × 10^–4^ for ApoB100), mutations in ApoB48/100 were more strongly associated with HCC (*P*_Cauchy_ = 6.07 × 10^–11^) than mutations in ApoB100 alone (*P*_Cauchy_ = 1.60 × 10^–3^). Concerning ApoB48/100 variants, the pooled OR for cirrhosis was 1.98 (95% CI: 1.24–2.14) for LoF/missense variants, and 2.90 (95% CI: 1.64–5.14) for LoF variants. The pooled OR for HCC was 4.04 (95% CI: 2.28–7.14) for LoF/missense variants and 10.26 (95% CI: 5.16–20.38) for LoF variants.

For ApoB100 variants, the pooled OR for cirrhosis was 1.98 (95% CI: 1.09–3.60) for LoF/missense variants and 3.96 (95% CI: 1.96–8.02) for LoF variants. The pooled OR for HCC was 2.30 (95% CI: 0.59–8.91) for LoF/missense variants and 10.19 (95% CI: 2.62–39.58) for LoF variants.

**Figure 4:**
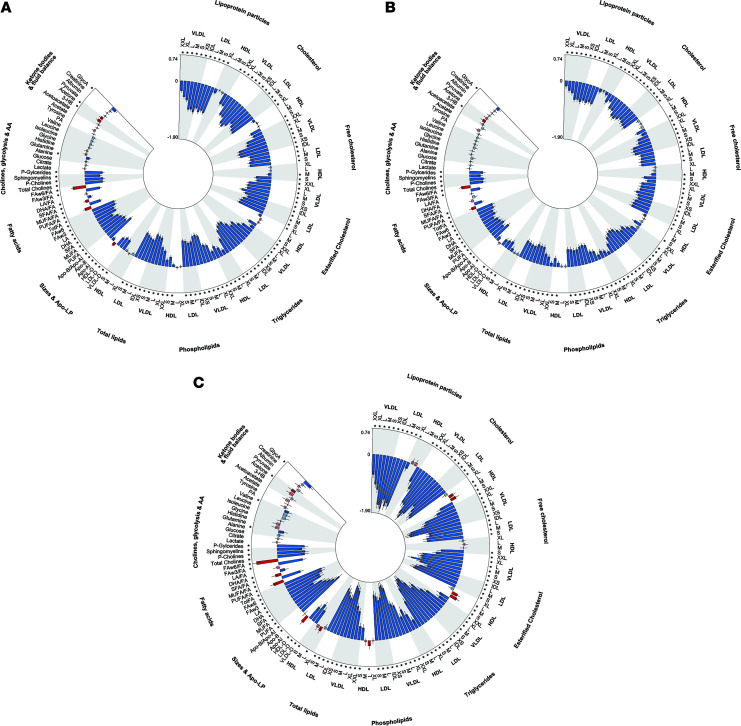
Circle plots for circulating lipoproteins, lipidomics, and metabolomics analysis of *APOB* LoF variants carriers in the UKBB. Associations of metabolic biomarkers in carriers with European ancestry from the UKBB. (**A**) Overall variants, (**B**) variants affecting both ApoB48/100s, and (**C**) variants affecting specifically ApoB100. *P* values were calculated using a whole-genome regression model as implemented in REGENIE; β coefficients (with 95% CIs) are presented per 1 SD change in metabolic biomarker, adjusted for age, sex, age2, age × sex, age2 × sex, BMI, first 10 PCs of ancestry and genotyping array. *Adjusted *P* < 0.05 (FDR controlled). Positive associations are displayed in red, whereas negative associations are blue. DHA, docosahexaenoic acid; FAw3, omega-3 fatty acid; FAw6, omega-6 fatty acid; HDL-D, high-density lipoprotein particle diameter; LA, linoleic acid; LDL-D, low-density lipoprotein particle diameter; LP, lipoprotein; MUFA, monounsaturated fatty acids; PUFA, polyunsaturated fatty acids; SFA, saturated fatty acids; VLDL-D, very low-density lipoprotein particle diameter.

To gain insight as to whether the differential effect of different types of mutations on liver outcomes correlated with the modulation of lipid metabolism, we next examined the effect of *APOB* LoF isoforms on circulating lipoproteins and CAD risk. Carriers of ApoB100 disrupting variants had lower LDL cholesterol, triglycerides, and ALT levels and higher liver triglyceride content and HDL cholesterol mirrored by protection against CAD (Figure 2). Interestingly, these differences were reduced in carriers of ApoB100/48 variants. Consistently, when we examined NMR metabolomics, carriers of ApoB variants had lower circulating lipid levels, accompanied by differences in other metabolic species (Figure 4A). Carriers of ApoB100 disrupting variants had an approximately 3-fold larger decrease in VLDL size, triglyceride levels within the VLDL subclasses, and biomarkers of lipoprotein metabolism, all suggesting a more severe impairment of VLDL secretion (Figure 4, B and C). These data support the idea of a larger effect of variants specifically affecting ApoB100 on circulating lipoprotein levels. While carriage of *APOB* variants was negatively associated with statin use (OR 0.61 and 0.67, for LoF and LoF/damaging variants, respectively), this inverse relationship was larger for variants disrupting ApoB100 than for those affecting ApoB48/100 (OR 0.18 and 0.52 vs. OR 0.79 and 0.78 for LoF and LoF/damaging variants, respectively, Supplemental Table 9).

The effect of *APOB* variants on circulating proteomics is reported in the supplemental material (Supplemental Figure 3 and Supplemental Table 11). Altogether, these data suggest that the larger effect of mutations affecting both ApoB48/100 and ApoB100 on HCC risk is mediated through mechanisms independent of lipid retention in hepatocytes that may involve impaired secretion of chylomicrons from enterocytes.

## Discussion

The main finding of this study is that rare *APOB* LoF variants, causing heterozygous hypobetalipoproteinemia, confer a strong predisposition to severe MASLD, namely cirrhosis and HCC. This predisposition was consistently observed across clinical cohorts, familial cosegregation analyses, and population-based studies. Accordingly, the severity of clinical outcomes increased with the predicted functional effect of the variants and the elevated HCC risk was present in both people of European ancestry and those of African ancestry and across the entire steatotic liver disease (SLD) spectrum.

Here, we started by showing that *APOB* variants are markedly (13.8-fold) enriched in people with advanced MASLD compared with the general population. Although hypobetalipoproteinemia is rare, it accounted for a substantial fraction (4.5%) of people with advanced MASLD in our large tertiary care cohort, suggesting a role for targeted genetic testing in people with advanced fibrosis or HCC and low LDL cholesterol levels. Clinically, *APOB* variant carriers had lower LDL cholesterol and triglyceride levels and higher HDL cholesterol levels, with lower circulating LDL cholesterol levels identifying with high accuracy carriers of more severe *APOB* variants. Furthermore, this latter group of carriers showed more severe hepatocyte ballooning, higher indices of fibrosis, and portal hypertension.

While common genetic variants account for a substantial fraction of MASLD heritability, they do not explain familial progression to advanced fibrosis (17). In contrast, *APOB* variants segregated with severe liver steatosis and fibrosis, demonstrating that heterozygous familial hypobetalipoproteinemia contributes to familial MASLD clustering and suggesting a role for genetic cascade screening in proband families.

By combining multiple cohorts at a cross-ancestry level, we show that rare LoF *APOB* variants increase the risk of HCC and confirmed previous data on the association with cirrhosis and chronic liver disease. However, our analysis revealed that *APOB* variants confer a 2-fold larger increase in the risk of HCC compared with cirrhosis in the MVP and UKBB cohorts. In line with previous observations (21, 22), these findings reinforce the notion that (a) *APOB* variants cause chronic liver disease, and (b) hepatic lipid accumulation has a direct carcinogenic effect on the liver. Moreover, they provide a granular evaluation of the effect of a specific class of mutations, depending on whether only the hepatic isoform (ApoB100) or the intestinal and hepatic isoform (ApoB48/100) was affected.

Mechanistically, *APOB* variants drive MASLD by impairing lipid export and thus resulting in hepatic lipid accumulation and inflammation. Carriers had elevated hepatic lipid, CRP, and HbA1c levels and a higher risk of diabetes, despite similar BMIs. These data are in line with the notion that hepatic lipid retention promotes inflammation, insulin resistance, and T2D, as previously shown by Mendelian randomization (14). The underlying mechanism seems to be driven by lipotoxicity and may be mediated by a reduction in insulin clearance, an induction of hepatic insulin resistance, oxidative stress, and the release of inflammatory mediators (23, 24).

A key insight from this study is the marked phenotypic divergence between variants affecting both ApoB48 and ApoB100 and those selectively affecting ApoB100. Mutations disrupting both isoforms, which also may impair chylomicron assembly in enterocytes (25), were associated with more severe liver disease and elevated HCC risk, despite modest changes in circulating lipoproteins and diminished protection against CAD. Conversely, ApoB100 mutations led to a greater reduction in lipoproteins and higher hepatic triglyceride content and circulating glucose levels. Notably, although these ApoB mutations conferred stronger protection against CAD, they showed only a weak association with HCC, despite comparable degrees of liver inflammation.

Taken together, these findings suggest that (a) in carriers of *APOB* LoF variants, hepatic lipid accumulation is necessary but not sufficient to drive HCC; (b) in carriers of ApoB48/100 variants additional extrahepatic tumor promoter mechanisms, namely impaired chylomicron secretion leading to intestinal barrier dysfunction and alterations of the extracellular matrix, dysbiosis, and oxidative stress via malabsorption of lipids and fat-soluble antioxidants (e.g., vitamin E), may further contribute to hepatocarcinogenesis (26), even though previous studies did not find lower lipid absorption following standardized meals in heterozygotes carrying ApoB48/100 variants (27); (c) an impairment of mitochondrial activity in carriers of ApoB48/ApoB100 variants may underpin the higher HCC risk. Indeed, the increased ratio of β-hydroxybutyrate over acetoacetate observed in carriers of these variants may reflect reduced NADH production, a mechanism linked to hepatic carcinogenesis and oxidative stress susceptibility (28) and to common genetic determinants of MASLD (29). However, more severe mutations affecting ApoB48 function may have been underrepresented or excluded, as they are likely to cause pronounced pediatric manifestations due to impaired lipid absorption during critical developmental periods; and (d) in carriers of ApoB100 specific variants, who have a more favorable lipoprotein profile, higher HDL levels may confer protection against malignant transformation of liver disease (30). Further mechanistic validation and experimental data are required to test these hypotheses.

These findings have several clinical implications. First, they reinforce the notion that hepatic lipoproteins retention drives MASLD (12, 14) and T2D (14), while also protecting against cardiovascular disease (15). Second, these findings support genetic screening for *APOB* variants in people with advanced MASLD and low LDL cholesterol, alongside family cascade screening (17, 31). Finally, combining rare variants with polygenic risk scores could further refine MASLD stratification, enabling precision medicine approaches (16, 32). In this respect, the present study demonstrates a higher penetrance of LoF ApoB48/100 mutations in hepatic carcinogenesis compared with LoF ApoB100. On the other hand, ApoB100 mutations protected against CAD, probably by more selective impairment of lipoprotein metabolism.

This study has some limitations related to its retrospective design, although it is fair to say that germline mutations are randomized at birth and present during a person’s entire lifespan. Moreover, liver histology was not systematically available, and the study populations were limited to those of European or African ancestry.

In conclusion, rare *APOB* LoF variants increase the risk of advanced MASLD, particularly HCC, with distinct effects based on their effect on ApoB isoforms. These insights highlight the detection of *APOB* mutations as a promising tool for case finding, risk stratification, and early intervention in advanced MASLD.

## Methods

### Sex as a biological variable

Both sexes were included in this study. In the Milan cohort, a total of 498 male participants and 273 female participants were enrolled.

### Study cohorts

#### Severe MASLD case-control cohort.

The severe MASLD case-control cohort comprises the EPIDEMIC-NAFLD (now MASLD), a cross-sectional Italian multicenter case-control study cohort aimed at the identification of genetic variants predisposing to the development of HCC in unrelated individuals with MASLD including ethnically matched controls, and the prospective SERENA study of consecutive patients with MASLD with advanced liver fibrosis without HCC at baseline. Part of this cohort has previously been described (33).

The enrollment, phenotyping, and genotyping of this cohort are described in the Supplemental Methods; the clinical features of the patients and controls are presented in Supplemental Table 1.

#### Family study.

Evaluation of first-degree family members of probands with severe MASLD bearing rare coding *APOB* variants was conducted at the Milan center within the RF-2016-02364358 project (17), as described in Supplemental Methods. The clinical features are reported in Supplemental Table 2.

For the meta-analysis of the effect of rare *APOB* variants on MASLD-related phenotypes, the literature was systematically searched for articles published in the English language containing “NAFLD,” MAFLD,” “steatosis,” “fatty liver disease,” “cirrhosis,” “hepatocellular carcinoma,” AND [“ApoB” OR “apob” OR “Apolipoprotein B”]. References of identified articles were also assessed for possible referral to other publications. Six articles were identified as reporting independent families for whom the carriage of rare pathogenic *APOB* variants was linked to liver traits (1, 32, 34–37).

#### MVP cohort.

Liver disease characterization was performed as described previously (38).

#### UKBB.

Liver disease and genetic characterization were performed as described previously (2, 16) and are reported in the supplemental material. Metabolomics, lipidomics, and proteomics analyses are described in the supplemental material.

### Definition of APOB variants

For the main analysis in the clinical cohort, we considered the following criteria to select likely LoF and damaging variants in *APOB* based on the literature (definition A being more liberal and B more restrictive; see the supplemental material).

In the UKBB and the MVP, we conducted a main analysis focusing on LoF mutations in *APOB,* either rare (minor allele frequency [MAF] <0.01) or ultrarare (singletons). We conducted an additional analysis selecting likely damaging missense variants when at least 4 of the following in silico prediction criteria were consistent with high pathogenicity: REVEL ≥0.5, CADD ≥20, SIFT, PolyPhen, LRT, MutationTaster, M-CAP, and AlphaMissense.

To discriminate whether the effect of *APOB* mutations differed according to the lipoproteins affected, we considered 3 sets of *APOB* variants: LoF in the whole *APOB* and stratified according to the genomic location based on the predicted effect on both ApoB48/100 and ApoB100 (upstream of amino acid 2154) or specifically on ApoB100 (downstream of amino acid 2154).

### Statistics

For descriptive statistics, categorical variables are shown as numbers and proportions. Continuous variables are shown as the median and IQR, as appropriate. Observational associations were performed by fitting data to generalized linear models. Logistic models were fit to examine binary traits, such as the presence of MASLD and advanced liver fibrosis. Analyses were adjusted for the main clinical and genetic confounders. Non-normally distributed variables were log-transformed before entering the analyses. Missing values in less than 5% of cases were imputed on the basis of the sex-specific median.

To examine the specific contribution of common SLD genetic risk variants to the inheritance of SLD and advanced fibrosis, we applied the transmission disequilibrium test (TdT), testing the overtransmission of risk alleles to affected family members as compared with chance inheritance (39).

In the UKBB and MVP, the association between liver outcomes or lipidomics and metabolic data and burden of rare (MAF < 0.01) LoF *APOB* variants was tested using a whole-genome regression approach implemented in REGENIE, as detailed in the Supplemental Methods.

Statistical analysis was carried out using JMP Pro 18.0.2 Statistical Analysis Software (SAS Institute) and R statistical analysis software, version 4.3.2 (http://www.R-project.org/). *P* values of less than 0.05 (2-tailed) were considered significant. To determine significance, we used the Exact Fisher test for categorical variables, and Kruskal-Wallis for continuous and ordinal variables; linear regression models were applied where appropriate. In figures, continuous variables were expressed as means ± SD or medians (interquartile range) for normally and not normally distributed variables, respectively. Categorical variables were expressed as absolute and relative frequencies (*n*, %).

### Study approval

Informed consent was obtained from all patients enrolled in the Milan Cohort and the family study. The MVP biobank study is a national multicenter biorepository linked to longitudinal electronic health records data from the United States Veterans, who provided informed consent to participate in the MVP Cohort study (MVP000), which was approved by Central Institutional Review Board of the Veteran Health Administration (VHA) Office of Research and Development. Data used in this study were obtained under MVP003/028 “Genetics of Cardiometabolic Diseases in the VA Population.” The UKBB study was approved by the North-West Multicenter Research Ethics Committee (reference no. 11/NW/0382). Data used in this study were obtained under Application Number 37142, Centre for Reproduction, Metabolism and Molecular medicine (CeRM), Department of Medicine (H7), Karolinska Institute, Huddinge, Sweden.

### Data availability

Clinical and *APOB* genetic data on the Milan cohort are provided in the Supporting Data Values file. UKBB data are available upon request. The summary statistics for genetic analyses in the study cohorts are available at: (https://github.com/Ojami/APOB-WES-analysis/tree/333c923815d05d545935b1efe9398b23e3fdded6). All code and scripts used for analyses are available at this location also.

## Authors contributions

LVCV, SR, S Pelusi, and MM conceptualized the work. LVCV, SR, MM, CVS, OJ, AC, and RMM drafted the manuscript. PP edited the manuscript. S Petta, GP, LR, MM, S Pelusi, LVCV, MS, VLM, RD, FPR, RDA, MF, L Miele, UVG, and EB enrolled the clinical study participants and collected clinical data. VM, L Miano, FM, and HET ran the genetic and bioinformatics analyses of the clinical cohort data. MM, S Pelusi, OJ, L Miano, and MV performed the clinical cohort analyses. MM, S Pelusi, and LR performed the family study. OJ and CVS performed analysis of UKBB data. MV, CTT, JAL, JPJ, and PST performed analysis of MVP data. LVCV, SR, CVS, KMC, and DP supervised the conduct of the study. LVCV, SR, and SA funded the study. All authors read and approved the final version of the manuscript. The order of the co–first authors’ names has been openly discussed and approved by all co–first and co–senior authors.

## Conflict of interest

LV reports receiving speaking fees from Viatris, Novo Nordisk, GSK and consulting fees from Novo Nordisk, Pfizer, Boehringer Ingelheim, Resalis, and Almac. SR received research grants from Novo Nordisk for research on diabetes complications and from AstraZeneca for research on steatotic liver disease; consults for AstraZeneca, GSK, Celgene Corporation, Ribocure AB, Madrigal, Ultragenyx, Amgen, Sanofi, Wave Life Sciences, Lipigon, Novartis, Profluent, Aina, and Echosens and Chiesi; declares equity in Heptabio; and is an inventor on a patent with title “Method for Treating Fatty Liver Disease” on PSD3 (US application number 17,480266 filed on September 21, 2021).

## Funding support

This work is the result of NIH funding, in whole or in part, and is subject to the NIH Public Access Policy. Through acceptance of this federal funding, the NIH has been given a right to make the work publicly available in PubMed Central.

- National Institute for Diabetes and Digestive and Kidney Diseases, R01DK134575 (to MV).
- Italian Ministry of Health (Ministero della Salute), Ricerca Finalizzata 2021 RF-2021-12373889, Italian Ministry of Health, Ricerca Finalizzata PNRR 2022 “RATIONAL” PNRR-MAD-2022-12375656 (to LVCV).
- Italian Ministry of Health (Ministero della Salute), Fondazione IRCCS Ca’ Granda Ospedale Maggiore Policlinico, Ricerca Corrente (to LVCV and DP).
- The European Union, H2020-ICT-2018-20/H2020-ICT-2020-2 programme “Photonics” under grant agreement No. 101016726 - REVEAL (to LVCV).
- The European Union, HORIZON-MISS-2021-CANCER-02-03 programme “Genial” under grant agreement “101096312” (to LVCV and SP).
- Italian Ministry of Research (MUR) PNRR – M4 - C2 “National Center for Gene Therapy and Drugs based on RNA Technology” CN3, Spoke 4 “ASSET” (to LVCV).
- PRIN 2022 MUR: “Disentangling genetic, epigenetic and hormonal regulation of Fe/heme metabolism in the gender-specific nature of NAFLD (DEFENDER).”
- Bando Ricerca Corrente and Piano Nazionale Complementare Ecosistema Innovativo della Salute – Hub Life Science-Diagnostica Avanzata (HLS-DA) – PNC-E3-2022-23683266 – “INNOVA” (to LVCV).
- Department of Pathophysiology and Transplantation, University of Milan, funded by the Italian Ministry of Education and Research (MUR): Dipartimenti di Eccellenza Program 2023 to 2027) (to LVCV).
- VA Merit Funding BX003362: “Genetics of Cardiometabolic Diseases in the VA Population” from the VA Office of R&D, VHA, USA (to KMC and PST).
- The Swedish Cancerfonden (22 2270 Pj), the Swedish Research Council (Vetenskapsradet (VR), 2023-02079), the Swedish state under the Agreement between the Swedish government and the county councils (the ALF agreement, ALFGBG-965360), the Swedish Heart Lung Foundation (20220334), the Novonordisk Distinguished Investigator Grant – Endocrinology and Metabolism (NNF23OC0082114), the Novonordisk Project grants in Endocrinology and Metabolism (NNF24OC0091535) (to SR).
- This research is also based on data from the MVP, the Office of Research and Development, and the VHA and was supported by MVP000 as well as by a VA Merit Award for MVP003/028 #BX003362 (to KMC) and the Department of Veterans Affairs Informatics and Computing Structure (VINCI), including data analytics conducted by its Precision Medicine research team, which is funded under the research priority to Put VA Data to Work for Veterans (VA ORD 24-D4V-02).

## Supplementary Material

Supplemental data

ICMJE disclosure forms

Supporting data values

## Figures and Tables

**Table 1:**
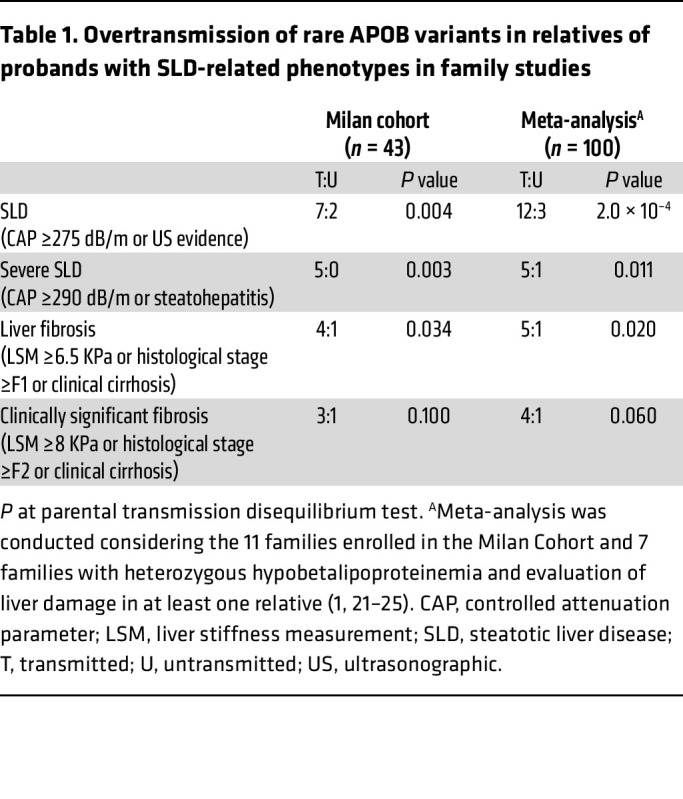
Overtransmission of rare APOB variants in relatives of probands with SLD-related phenotypes in family studies

## Version 1 02/10/2026

In-Press Preview

## Version 2 04/15/2026

Electronic publication
