# Supplementary material for: Carriage of rare APOB variants predisposes to severe steatotic liver disease and hepatocellular carcinoma
Source: J Clin Invest. 2026 Feb 10;136(8):e201762. doi: 10.1172/JCI201762 (PMC13078886; doi:10.1172/JCI201762)
Supplement: Supplemental data [file jci-136-201762-s105.pdf]

Carriage of rare *APOB* variants predisposes to severe steatotic liver disease and hepatocellular carcinoma

Matteo Mureddu[1]*, Serena Pelusi[2]*, Oveis Jamialahmadi[3]*, Marijana Vujkovic[4,5], Lorenzo Miano[1,2], Hadi Eidgah Torghabehei[6], Luisa Ronzoni[2], Francesco Malvestiti[1], Marco Saracino[7], Giulia Periti[2], Vittoria Moretti[2], Craig Teerlink[8,9], Julie A Lynch[8,9], Philip S Tsao[10], Josephine P Johnson[4,5], Vincenzo La Mura[1,11], Robertino Dilena[12], Saleh Alqahtani[13], Alessandro Cherubini[2], EPIDEMIC Study Investigators^, Million Veteran Program[#], Francesco Paolo Russo[14], Roberta D'Ambrosio[15], Mirella Fraquelli[16], Salvatore Petta[17], Luca Miele[18], Umberto Vespasiani-Gentilucci[19], Elisabetta Bugianesi[20], Rosellina M Mancina[3,21,22], Paolo Parini[25], Daniele Prati[2], Kyong-Mi Chang[4,5], Carolin V Schneider[23°], Stefano Romeo[3,24,25,26,27°], Luca VC Valenti[1,2°].

SUPPLEMENTARY INFORMATION 4

EPIDEMIC Study Investigators Acknowledgement list 4

Veteran Affairs (VA) Million Veteran Program (MVP): 4

SUPPLEMENTARY METHODS 6

Severe MASLD case-control cohort enrolment and characterization 6

Clinical cohort genotyping 6

Family study 7

UK Biobank cohort 7

Definition of APOB variants in the clinical cohort 8

Metabolomics, lipidomics and proteomics in UKBB cohort 8

Million Veteran Program 9

Gene-based analysis in biobanks 10

Cross-ancestry meta-analysis 11

SUPPLEMENTARY RESULTS 12

Combined effect of APOB variants and polygenic predisposition on MASLD 12

Proteomic analysis 12

SUPPLEMENTARY FIGURES 13

Supplementary Figure 1. 13

Supplementary Figure 3. 15

SUPPLEMENTARY TABLES 16

Supplementary Table 1. 16

Supplementary Table 2. 17

Supplementary Table 3. 18

Supplementary Table 4. 19

Supplementary Table 5. 20

Supplementary Table 6. 21

Supplementary Table 7. 22

Supplementary Table 8. 23

Supplementary Table 9. 25

Supplementary table 10. 26

Supplementary Table 11. 27

SUPPLEMENTARY REFERENCES **29**

SUPPLEMENTARY INFORMATION

EPIDEMIC Study Investigators Acknowledgement list

Fondazione IRCCS Ca' Granda Ospedale Policlinico Milano, University of Milan, Precision Medicine and Omics Lab.
Serena Pelusi, Giulia Periti, Vittoria Moretti, Stefania Mira, Sara Margarita, Luisa Ronzoni, Daniele Marchelli, Hadi Heidgah Torgabehei, Maria De Filippo, Alessandra Cazzaniga, Elena Sinopoli, Laura Cerami, Elia Casirati, Eniada Rrapaj, Divya Akkaldev, Francesco Malvestiti, Martina Tranchina, Valentina Vaira, Luca Valenti.

Fondazione IRCCS Ca' Granda Ospedale Policlinico Milano, University of Milan, Gastroenterology & Hepatology.
Roberta D'Ambrosio, Pietro Lampertico.

Fondazione IRCCS Ca' Granda Ospedale Policlinico Milano, University of Milan, Pathology.
Marco Maggioni.

Fondazione IRCCS Ca' Granda Ospedale Policlinico Milano, University of Milan, Internal Medicine and Metabolic Diseases.
Annalisa Cespiati, Paola Dongiovanni, Anna Ludovica Fracanzani.

Gastroenterology, University of Turin.
Chiara Rosso, Angelo Armandi, Elisabetta Bugianesi.

Gastroenterology, University of Palermo.
Grazia Pennisi, Salvatore Petta.

Policlinico Gemelli, Università Cattolica Roma.
Antonio Liguori, Luca Miele.

University of Udine.
Giorgio Soardo.

University of Campania "Luigi Vanvitelli" Naples.
Alessandro Federico.

Unicampus, Roma.
Federica Tavaglione, Umberto Vespasiani-Gentilucci.

Veteran Affairs (VA) Million Veteran Program (MVP):

Core Acknowledgements for Publications May 2024

MVP Program Office
- Sumitra Muralidhar, Ph.D., Program Director
US Department of Veterans Affairs, 810 Vermont Avenue NW, Washington, DC 20420
- Jennifer Moser, Ph.D., Associate Director, Scientific Programs
US Department of Veterans Affairs, 810 Vermont Avenue NW, Washington, DC 20420
- Jennifer E. Deen, B.S., Associate Director, Cohort & Public Relations
US Department of Veterans Affairs, 810 Vermont Avenue NW, Washington, DC 20420

MVP Executive Committee
- Co-Chair: Philip S. Tsao, Ph.D.
VA Palo Alto Health Care System, 3801 Miranda Avenue, Palo Alto, CA 94304
- Co-Chair: Sumitra Muralidhar, Ph.D.
US Department of Veterans Affairs, 810 Vermont Avenue NW, Washington, DC 20420
- J. Michael Gaziano, M.D., M.P.H.

VA Boston Healthcare System, 150 S. Huntington Avenue, Boston, MA 02130
- Elizabeth Hauser, Ph.D.
Durham VA Medical Center, 508 Fulton Street, Durham, NC 27705
- Amy Kilbourne, Ph.D., M.P.H.
VA HSR&D, 2215 Fuller Road, Ann Arbor, MI 48105
- Michael Matheny, M.D., M.S., M.P.H.
VA Tennessee Valley Healthcare System, 1310 24th Ave. South, Nashville, TN 37212
- Dave Oslin, M.D.
Philadelphia VA Medical Center, 3900 Woodland Avenue, Philadelphia, PA 19104
- Deepak Voora, MD
Durham VA Medical Center, 508 Fulton Street, Durham, NC 27705

MVP Co-Principal Investigators
- J. Michael Gaziano, M.D., M.P.H.
VA Boston Healthcare System, 150 S. Huntington Avenue, Boston, MA 02130
- Philip S. Tsao, Ph.D.
VA Palo Alto Health Care System, 3801 Miranda Avenue, Palo Alto, CA 94304

MVP Core Operations
- Jessica V. Brewer, M.P.H., Director, MVP Cohort Operations
VA Boston Healthcare System, 150 S. Huntington Avenue, Boston, MA 02130
- Mary T. Brophy M.D., M.P.H., Director, VA Central Biorepository
VA Boston Healthcare System, 150 S. Huntington Avenue, Boston, MA 02130
- Kelly Cho, M.P.H, Ph.D., Director, MVP Phenomics
VA Boston Healthcare System, 150 S. Huntington Avenue, Boston, MA 02130
- Lori Churby, B.S., Director, MVP Regulatory Affairs
VA Palo Alto Health Care System, 3801 Miranda Avenue, Palo Alto, CA 94304
- Scott L. DuVall, Ph.D., Director, VA Informatics and Computing Infrastructure (VINCI) VA Salt Lake City Health Care System, 500 Foothill Drive, Salt Lake City, UT 84148
- Saiju Pyarajan Ph.D., Director, Data and Computational Sciences
VA Boston Healthcare System, 150 S. Huntington Avenue, Boston, MA 02130
- Robert Ringer, Pharm.D., Director, VA Albuquerque Central Biorepository
New Mexico VA Health Care System, 1501 San Pedro Drive SE, Albuquerque, NM 87108
- Luis E. Selva, Ph.D., Director, MVP Biorepository Coordination
VA Boston Healthcare System, 150 S. Huntington Avenue, Boston, MA 02130
- Shahpoor (Alex) Shayan, M.S., Director, MVP PRE Informatics
VA Boston Healthcare System, 150 S. Huntington Avenue, Boston, MA 02130
- Brady Stephens, M.S., Principal Investigator, MVP Information Center Canandaigua VA Medical Center, 400 Fort Hill Avenue, Canandaigua, NY 14424
- Stacey B. Whitbourne, Ph.D., Director, MVP Cohort Development and Management VA Boston Healthcare System, 150 S. Huntington Avenue, Boston, MA 02130

MVP Publications and Presentations Committee
- Co-Chair: Themistocles L. Assimes, M.D., Ph. D
VA Palo Alto Health Care System, 3801 Miranda Avenue, Palo Alto, CA 94304
- Co-Chair: Adriana Hung, M.D.; M.P.H
VA Tennessee Valley Healthcare System, 1310 24th Ave. South, Nashville, TN 37212
- Co-Chair: Henry Kranzler, M.D.
Philadelphia VA Medical Center, 3900 Woodland Avenue, Philadelphia, PA 19104

SUPPLEMENTARY METHODS

Severe MASLD case-control cohort enrolment and characterization

The severe MASLD case-control cohort is made up of the EPIDEMIC-NAFLD (now MASLD) (“Exome sequencing for the identification of genetic mutations promoting hepatocellular carcinoma development in nonalcoholic fatty liver disease”), a cross-sectional Italian multicenter case-control study cohort aimed at the identification of genetic variants predisposing to the development of HCC in unrelated individuals with MASLD including ethnically matched controls, and the prospective “SERENA” study of consecutive patients with MASLD with advanced liver fibrosis without HCC at baseline. Part of this global cohort has previously been described (1).

MASLD diagnosis was based on the demonstration of steatosis by imaging at the time of study inclusion or a previous positive clinical history in patients with advanced disease, daily alcohol intake <30/20 g/day in males/females, and absence of concurrent liver diseases and other hepatotoxic factors (including chronic viral or autoimmune hepatitis; genetic liver diseases including hereditary hemochromatosis, Wilson’s disease, AAT deficiency, use of steatogenic/hepatotoxic drugs). All patients fulfilled the metabolic criteria for MASLD (2). Severe MASLD was diagnosed in presence of either histological evidence of liver fibrosis stage F2-F4, liver stiffness measurement (LSM) by Fibroscan© ≥8 kPa, or FIB-4 index ≥2.67. Advanced liver fibrosis and HCC were diagnosed according to the EASL criteria (3, 4).

Controls were ethnically matched apparently healthy individuals with no history of metabolic syndrome or of liver disease and apparently normal liver enzymes enrolled at the Fondazione IRCCS Ca’ Granda Hospital Milan.

The study protocol conformed to the ethical guidelines of the 1975 Declaration of Helsinki. The EPIDEMIC and SERENA study were approved by the Ethical Committee of the Fondazione IRCCS Ca’ Granda Ospedale Maggiore Policlinico Milan and participating centers (EPIDEMIC-TERT study Ethical approval n. 1882_2013; Perspective-SERENA multicenter Study approval n. 485_2017, Fondazione IRCCS Ca’ Granda Ethical Committee). Informed consent was obtained from each participant.

Clinical cohort genotyping

The bioinformatic pipeline for variant calling, annotation and quality control in the Milan cohort has previously been described (5, 6). DNA libraries were enriched for whole exome sequencing (WES) by the SureSelect Human All Exon v8 kit (Agilent, Milan, Italy). Sequencing was performed at the Fondazione Ca’ Granda Genomic Facility

on the Illumina NextSeq2000 with a minimal sequencing mean depth of 90x and Variant calling will be performed following Genome Analysis Toolkit (GATK) best-practice using GATK suite tools version 4.6.0 (7). Raw reads quality control was performed using FastQC 0.12.1 (Brabaham bioinformatics, Cambridge, UK) and Fastp 0.23.4 to remove low quality reads. Reads mapping on the human GRCh38 genome was performed using the MEM algorithm of Burrows Wheeler Aligner (BWA) 0.7.17-r1188 (8). Duplicate reads were marked by MarkDuplicate (Picard suite 3.2) and subsequently selected to generate high quality bam files (9). Mapping quality control was performed using Mosedepth 0.3.3 (10) and Bedtools 1.17 (11), variant calling using HaplotypeCaller 2.2.0. Variant quality score log-odds (VQSLOD) above 99% tranche were considered true positives and variants present in <20% of total reads discarded. Indel left normalization was performed using BCFtools (12), variant annotation using both Ensembl Variant Effect Predictor (VEP-v112) and Annovar version 20240301 (13).

All *APOB* genetic variants in the Italian severe MASLD and controls cohort were confirmed by Sanger sequencing. In family members, the specific variants detected in the probands were searched for by Sanger sequencing. The primer sequences were synthesized by Sigma Aldrich and are available upon request.

Family study

Evaluation of first-degree family members of probands with severe MASLD bearing rare coding *APOB* variants was carried on at the Milan center within the RF-2016-02364358 project (14). Briefly, relatives who consented to the study underwent clinical, metabolic and biochemical evaluation. Liver damage was non-invasively assessed by liver enzymes and Fibroscan with continuous attenuation parameter (CAP) and LSM measurement. All individuals gave written informed consent to study participation. Part of this cohort has previously been described (14).

The clinical features of probands, affected and unaffected family members are presented in Supplementary Table 2, and pedigrees in Supplementary Figure 2. In the family study all rare missense variants with CADD score ≥10 and or classified as VUS were included if at least one family member was available.

UK Biobank cohort

The UK Biobank study has been approved by the North-West Multicenter Research Ethics Committee (reference number 11/NW/0382). Data used in this study were obtained under the Application Number 37142. Liver disease and genetic characterization was performed as described previously (5, 15). Here, we considered only unrelated UKBB participants of European ancestry, and excluded individuals with withdrawn consent, excessive relatives,

a mismatch between the self-reported and genetically inferred gender, putative sex chromosome aneuploidy, and those who were identified by the UKBB as outliers. Individuals were excluded if they were diagnosed with other causes of liver disease, chronic viral hepatitis (B18, B19, E83.0, E83.1, K71, K74.3, K74.4, K74.5, K75.2, K75.3, K75.4, K75.8, K75.9) or any other type of cancer except C22.0. HCC was defined by combining ICD-10 code C22.0 from the mentioned resources (data-fields 41270, 40001, 40002, and 40006) and individuals without HCC or diagnosed with any other type of liver and intrahepatic bile ducts cancers (C22 except C22.0) were further excluded. Liver cirrhosis (K70.3, K70.4, K72.1, K72.9, K74.1, K74.2, K74.6, K76.6, K76.7, I85.0, I85.9) was defined using in-hospital admissions and death registry after excluding any diagnosis of chronic viral hepatitis (B18 and B19).

Whole-exome sequencing data for 450,000 subjects were released in October 2021 and accessed via the UKB DNAnexus platform from OQFE pipeline. Quality control (QC) metrics were applied to Variant Call Format (VCF), including genotype level filters for depth and genotype quality (10). We used final 500K release of WES with 20x sequence coverage on ~96% of sites and mapped to GRCh38 genome assembly using OQFE pipeline. We used the QC filters provided by UKBB Research Analysis Platform (RAP) on DNAnexus and excluded variants with <10 read depth in 90% of the samples. Furthermore, we excluded variants or samples with more than 15% missingness or Hardy–Weinberg equilibrium $P>10^{-15}$ [8, 10].

Definition of APOB variants in the clinical cohort

A required criterium was a minor allelic frequency (MAF) <0.01 in ExAC non-Finnish European (NFE) database and in the local ethnically-matched control group, plus either one of the following: a) frameshift, truncating, and exon skipping variants; b) variants previously linked in the literature with familial hypobetalipoproteinemia; c) missense variants with CADD score ≥20 and classified as pathogenic or likely pathogenic according to the American College of Medical Genetics and Genomics (ACMG) at July 2024; d) variants of uncertain significance (VUS) limited to exon 1-25 (excluding those in exon 26 exclusive of ApoB100 and less likely to be complete LoF). As a sensitivity analysis, to keep more robust criteria, we also used an alternative definition excluding criterion (d) (definition B) or considering only pathogenic predicted loss of function (LoF) mutation (definition C). Definition A was used to compare clinical characteristics of severe MASLD subjects carrying *APOB* variants.

Metabolomics, lipidomics and proteomics in UKBB cohort

The association analyses between 143 plasma nuclear magnetic resonance (NMR) biomarkers in n=234,843 Europeans from UK Biobank and LoF variants on APOB were performed using whole-genome regression analysis as implemented in REGENIE. All analyses were adjusted for age, sex, age^2, age×sex, age^2×sex, BMI, first 10 principal components (PCs) of ancestry, and genotyping array (16). For visualization, circle plots were generated using the "circlize" R package to highlight associations and confidence intervals for metabolites. Metabolites were categorized based on lipid classes, fatty acids, and other metabolic categories.

Plasma proteomic data were available for approximately 3000 plasma proteins in 39,103 unrelated European participants from the UKBB (17). Using a burden test approach, we examined the association between LoF and LoF/missense APOB variants and 2923 plasma proteins through linear regression analysis adjusted for age, sex, age^2, age×sex, age^2×sex, BMI, first 10 principal components (PCs) of ancestry, genotyping array and batch. For each protein-isoform pair, the p-values from LoF and LoF/missense masks were then combined using Cauchy distribution (18). The associations with a Benjamini–Hochberg false discovery rate (FDR) <0.05 were considered significant. We then uploaded the significant proteins for each isoform on Metascape to examine common metabolic pathways altered by *APOB* impairment (Supplementary Table 9). The selection of pathways of interest was performed as previously described (12).

All plasma NMR biomarkers and protein levels were rank-based inverse normal transformed prior to the analyses.

Million Veteran Program

The Million Veteran Program (MVP) generated deep whole-genome sequencing (WGS) data for 109,826 participants, processed using the Broad Institute's GATK "$5 genome" pipeline (https://github.com/gatk-workflows/five-dollar-genome-analysis-pipeline). Reads were aligned to GRCh38 with BWA-MEM (v0.7.15), compressed to CRAM, and variant calling was performed using GATK 4.1.0.0 in gVCF mode, and multi-level quality control was implemented which consisted of sequencing QC using FastQC, alignment QC using SAMtools and verifyBamID, and variant QC using RTG Tools. Stringent filters were applied at the sample, variant, and genotype levels. Samples required ≥97% call rate, ≥18x mean depth, and <5% contamination. Genotype-level thresholds varied by genotype type, incorporating quality, allele balance, and depth criteria. Variants required ≥80% call rate and presence of at least one alternate allele.

After QC, 102,677 individuals and 663,351,127 variants remained for analysis. All variants were annotated using Ensembl VEP (v110), focusing on rare, high-impact variants in APOB. Predicted loss-of-function (pLoF) variants were defined as frameshift, stop-gain/loss, splice-site, or start/stop codon disruptions in canonical transcripts, and

flagged as "High Confidence" by LOFTEE. Rare deleterious missense variants were defined as those predicted "likely pathogenic" by AlphaMissense, also limited to the canonical transcript and isoforms. Variants with minor allele frequency <1% were considered rare.

HCC was defined using national cancer registry data. Cirrhosis was identified using a combination of ICD-9/10 codes—either at least two codes for cirrhosis (K70.3, K74.6), or one cirrhosis code plus one code indicating a cirrhotic complication (I85.0, I85.1, I86.4, K72.9, R18.8, K70.11, K70.31, K65.2, K76.6, K76.7, K76.81). To enhance specificity for cirrhosis, a laboratory-based fibrosis score (Fib-4 > 2.67) was required at the time of diagnosis. Controls were individuals with no documented history of cirrhosis or HCC.

Gene-based analysis in biobanks

In UKBB and MVP, the association between liver and cardiometabolic outcomes and burden of rare (MAF <0.01) LoF *APOB* variants was tested using a whole-genome regression approach implemented in REGENIE (15, 19). For liver cirrhosis and HCC, the analyses were adjusted for age, sex, age^2, age×sex, age^2×sex, first 10 PCs of ancestry and genotyping array. All other cardiometabolic and liver outcomes were further adjusted for BMI, smoking status (never/previous/current), and alcohol consumption (grams per day), with additional adjustments for diabetes and hypertension in the case of coronary artery disease. To fit the whole-genome regression model in step 1 of REGENIE, a subset of directly genotyped common variants (MAF > 1%) was used. After excluding variants on long-range linkage disequilibrium (LD) and major histocompatibility complex regions, variants with a missingness <0.01, and with Hardy–Weinberg equilibrium P>1E-15 were retained. Finally, 146,833 markers left following an LD pruning with a window of 500,000 base pairs and pairwise $r^2<0.8$. For binary traits with a nominal P<0.05, we re-estimated OR and SE using Firth's logistic regression in REGENIE-FIRTH (20).

We used high-confidence LoF Variants as determined by VEP LOFTEE. Deleterious missense variants were selected based on an AlphaMissense pathogenicity score between 0.564 and 1, which corresponds with a classification of "likely pathogenic"

Predicted LoF genetic variants include (a) insertions or deletions resulting in a frameshift, (b) insertions, deletions or single nucleotide variants resulting in the introduction of a premature stop codon or in the loss of the transcription start site or stop site, and (c) variants in donor or acceptor splice sites. Functional annotation is restricted to canonical transcripts in protein coding genes only, and only LoFs flagged as High Confidence by LOFTEE were included.

Cross-ancestry meta-analysis

We performed a cross-ancestry inverse-variance-weighted fixed-effect meta-analysis of the three studies comprising two ancestries (European and African) using METAL version “2020-05-05” (21). For cirrhosis or HCC, the meta-analysis was performed for each isoform-mask separately, using the log Firth’s odds ratio of each study. The definition criteria for each mask were the same for all studies. We then combined the meta-analysis P-values for each isoform across masks using the Cauchy distribution (18).

Supplementary Results

Combined effect of APOB variants and polygenic predisposition on MASLD

In the clinical case-control cohort, carriage of *APOB* variants had a greater impact on liver disease severity in patients with otherwise lower polygenic risk. Among subjects with MASLD and PRS-5 <0.495 (n= 200), *APOB* carriers displayed lower platelet levels (148±49 vs. 198±92 10^3/µl, p=0.01), a higher prevalence of advanced fibrosis (91.7% vs. 62.2%, p=0.04) and, in the 78 subjects with biopsy available, higher ballooning score (1[1-2] vs. 1[0-1], p=0.03). In the subgroups with higher PRS-5 (n=124), carriers only differentiated due to lower total cholesterol (125±54 vs. 173±44 mg/dl, p=0.045) and lower triglycerides (62±43 vs. 125±74 mg/dl, p=0.045). Similarly, in patients homozygous for *PNPLA3* p.I148M MASLD severity did not differ based on the carriage of *APOB* variants (not shown). This observation was confirmed in the UKBB, where carriage *APOB* and PRS-5 showed an additive effect, but non-significant multiplicative interactions on the risk of liver outcomes (not shown).

Proteomic analysis

We evaluated a subset of 39,103 unrelated Europeans participating in the UKBB for whom a profile of 2923 plasma proteins were available (Supplementary Table 11). Through multiple linear regression analysis, we evaluated statistically significant differences in plasma protein based on *APOB* variant status and reported those significantly associated with carriage of LoF variants after FDR correction (Supplementary Table 10). Deregulated pathways are shown in Supplementary Figure 3. While variants affecting only ApoB100 were associated with downregulation of lipoprotein metabolism pathways and protein translation, those affecting both ApoB48/100 reduced the inhibition of inflammatory response and extracellular matrix modelling.

SUPPLEMENTARY FIGURES

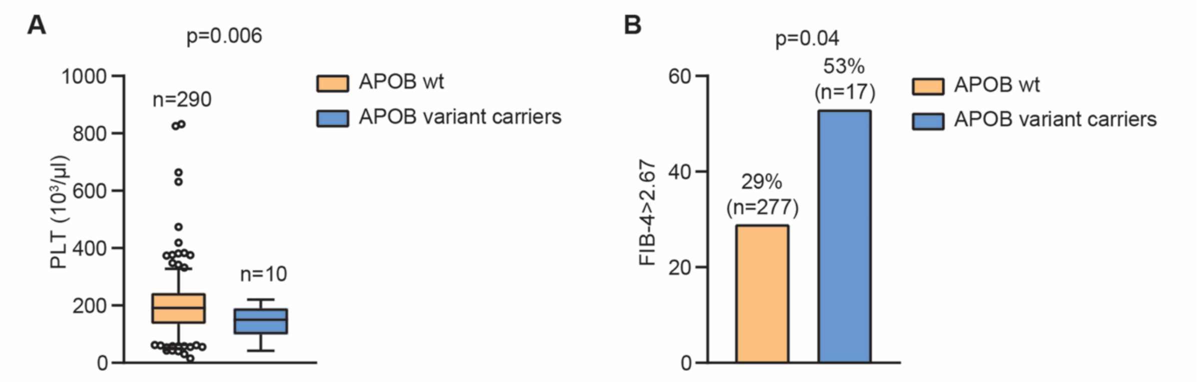

Supplementary Figure 1. Impact of the *APOB* genotype on MASLD outcomes in the clinical cohort (n=510) in which the data was available. A) PLT distribution in 300 individuals, stratified according to the presence of the *APOB* variants. In the box and whisker plots, the line in the middle of the box represents the medians, tops and bottoms of the boxes the 25th and 75th quartiles, respectively, and the whiskers the minimum to maximum value. Symbols represent 5th and 95th percentiles, respectively. p value was determined by Mann-Whitney U test. B) Bar chart showing the percentage of FIB-4 >2.67 between individuals *APOB* wt and variant carriers. p value was obtained through the Kruskal-Wallis test.

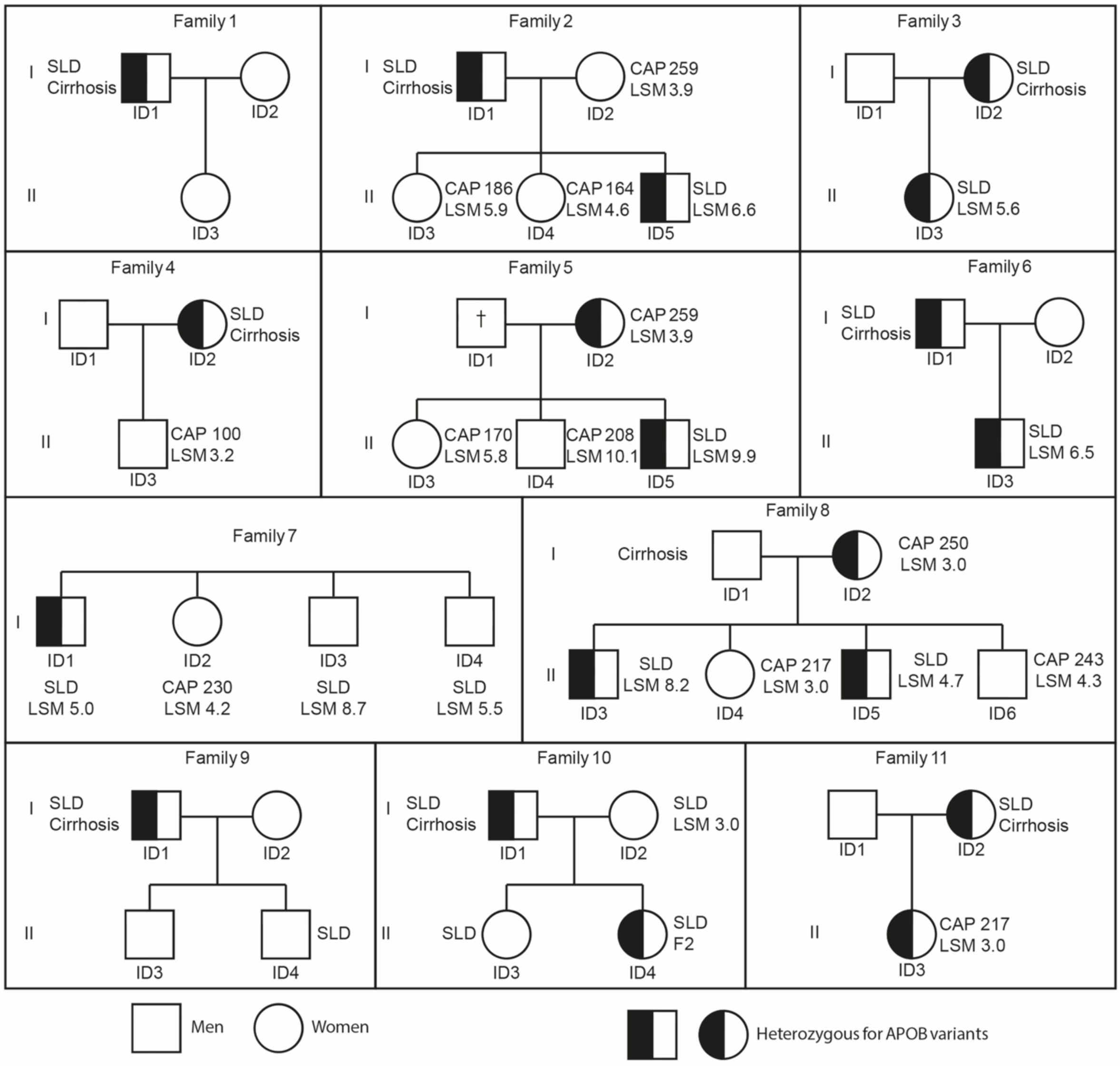

Supplementary Figure 2. **Pedigree charts of the families included in the study.** Circles denote female family members, squares male family members. Half shaded symbols indicate carriers for *APOB* variants. Black arrow indicates probands.

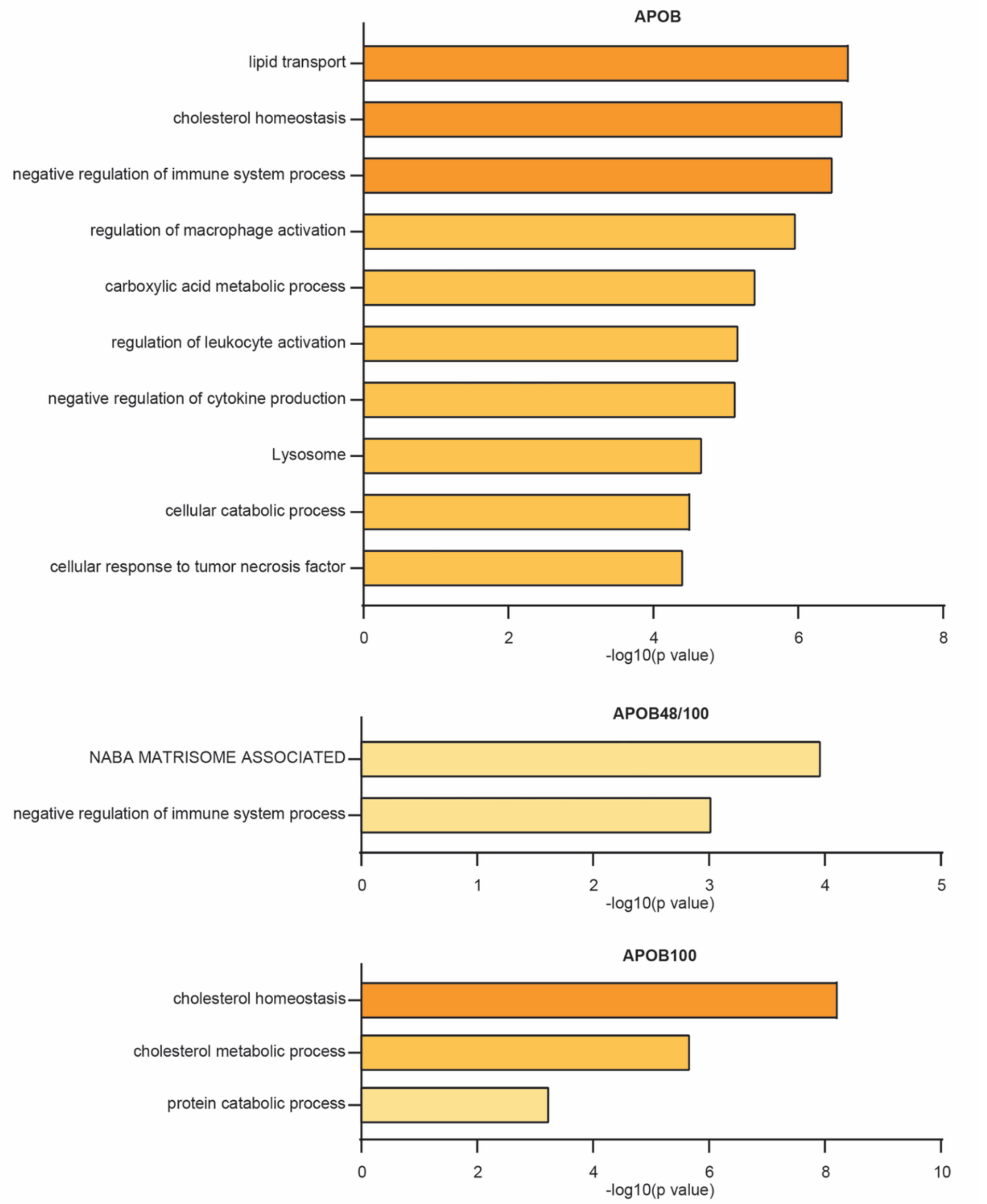

Supplementary Figure 3. Pathway deregulation based on significant *loci* identified by pQTLs analysis. We first identified all statistically enriched terms, accumulative hypergeometric p-values and enrichment factors were calculated and used for filtering. Remaining significant terms were subsequentially hierarchically clustered into a tree based on Kappa-statistical similarities among their gene memberships. Then 0.3 kappa score was applied as the threshold to cast the tree into term clusters.

Supplementary tables

Supplementary Table 1. Clinical features of the severe MASLD case-control cohort.

	**Severe MASLD (n=510)**	**Controls (n=261)**	***p* value***
Age, years	62 [52-70]	41[30-52]	<0.001
Sex, M	341 (67)	157 (60)	0.08
BMI, Kg/m^2	29 [26-33]	23 [22-25]	<0.001
Total cholesterol, mg/dl	171 [130-211]	175 [160-191]	0.21
HDL, mg/dl	48 [38-59]	68 [56-80]	<0.001
Triglycerides, mg/dl	117 [74-160]	63 [49-83]	<0.001
LDL, mg/dl	101 [76-136]	92.8 [78-111]	0.004
Statin use, yes	36 (7)	0	<0.001
T2D, yes	250 (49)	0	<0.001
Hypertension, yes	302 (59)	0	<0.001
ALT, IU/l	39 [27-59]	18 [14-22]	<0.001
GGT, IU/l	56 [31-118]	13 [10-18]	<0.001
PLTs, 10^3/μl	190 [134-240]	232 [196-267]	<0.001
LSM, kPa	11 [6-21]	NA	NA
Cirrhosis, yes	204 (40)	0	<0.001
HCC, yes	168 (33)	0	<0.001
Available liver biopsy	167 (33)	NA	NA
PNPLA3 p.I148M, M/M	147 (29)	14 (5)	<0.001

Data are shown as n (%), median [IQR]. BMI: body mass index; HDL: high-density lipoprotein cholesterol; LDL: low-density lipoprotein cholesterol; ALT: alanine aminotransferases; GGT: gamma-glutamyl transferases; PLTs: platelets; LSM: liver stiffness measurement by Fibroscan; HCC: hepatocellular carcinoma; PNPLA3: patatin-like phospholipase domain containing 3. *At univariate logistic regression models.

Supplementary Table 2. Clinical features of probands carrying rare *APOB* variants and their first-degree relatives included in the family study.

	Age	Sex	A*POB* status	Steatosis	Fibrosis	Cirrhosis	HCC
**Family 1**							
mother	56	F	wt	NA	NA	NA	NA
proband	57	M	Het p.R427*	yes	yes	yes	no
daughter	38	F	wt	no	no	no	no
**Family 2**							
mother	58	F	wt	no	no	no	no
father	65	M	Het p.S2429*	yes	yes	yes	no
sister	32	F	wt	no	no	no	no
sister	28	F	wt	no	no	no	no
proband	21	M	Het p.S2429*	yes	yes	no	no
**Family 3**							
mother	71	F	Het p.Q3176*	yes	yes	yes	yes
father	72	M	wt	NA	NA	NA	NA
proband	49	F	Het p.Q3176*	yes	yes	no	no
**Family 4**							
proband	76	F	het rs142151703^	yes	yes	yes	no
father	77	M	wt	NA	NA	NA	NA
son	46	M	wt	no	no	no	no
**Family 5**							
mother	65	F	Het c.3696+2T>G	yes	no	no	no
father	†	M	wt	NA	NA	NA	NA
sister	35	F	wt	no	no	no	no
brother	29	M	wt	no	yes	no	no
proband	28	M	Het c.3696+2T>G	yes	yes	no	no
**Family 6**							
mother	63	F	wt	no	no	no	no
father	66	M	Het p.L1801P	yes	yes	yes	no
proband	27	M	Het p.L1801P	yes	yes	no	no
**Family 7**							
proband	54	M	Het p.V730I	yes	no	no	No
sister	51	F	wt	no	no	no	no
brother	46	M	wt	yes	yes	no	no
brother	36	M	wt	yes	no	no	no
**Family 8**							
mother	77	F	wt	no	no	no	no
father	88	M	Het p.L367fs	yes	yes	yes	no
proband	52	M	Het p.L367fs	yes	yes	no	no
sister	47	F	wt	no	no	no	no
brother	43	M	Het p.L367fs	yes	no	no	no
brother	40	M	wt	no	no	no	no
**Family 9**							
mother	77	F	wt	no	no	no	no
proband	72	M	Het p.E3969Nfs*38	yes	yes	no	no
son	57	M	wt	no	no	no	no
son	53	M	wt	yes	no	no	no
**Family 10**							
mother	80	F	wt	yes	no	no	no
proband	82	M	Het p.G753E	yes	yes	yes	no
daughter	59	F	wt	yes	no	no	no
daughter	57	F	Het p.G753E	yes	yes	no	no
**Family 11**							
proband	67	F	Het p.I2156T	yes	yes	yes	no
father	69	M	wt	no	no	no	no
daughter	47	F	wt	no	no	no	no

Het: heterozygosity; NA: not available; HCC: hepatocellular carcinoma; †: deceased; ^ (*179C>T) affecting 3'-UTR and classified as VUS.

Supplementary Table 3. Clinical features of patients with severe MASLD (n=510) stratified according to carriage of *APOB* variants (definition A) in the Milan case-control clinical cohort.

	**Carriers (n=23)**	**Non-carriers (n=487)**	***p* value***
Age, years	58 [53-70]	62 [51-70]	0.360
Sex, M	15 (65)	306 (63)	0.990
BMI, Kg/m^2	30 [27-33]	29 [26-33]	0.830
Total cholesterol, mg/dl	160 [111-174]	174 [131-212]	0.180
HDL-cholesterol, mg/dl	53 [39-68]	47 [38-59]	0.044
Triglycerides, mg/dl	66 [58-124]	118 [77-162]	0.016
LDL-cholesterol, mg/dl	66 [44-96]	106 [78-138]	0.0007
Statin use, yes	0	36 (7.4%)	0.69
Type 2 diabetes, yes	10 (43)	214 (44)	0.870
HbA1c, mmol/l	46.4±11.0	46.0±15.6	0.70
Arterial hypertension, yes	14 (61)	242 (50)	0.280
AST, IU/l	38 [27-53]	36 [26-51]	0.920
ALT, IU/l	40 [24-61]	39 [27-59	0.550
GGT, IU/l	74 [17-111]	60 [31-118]	0.680
PLTs, 10^3/µl	150 [99-190]	191 [135-244]	0.001
FIB-4, units^	9/17 (53)	82/277 (29)	0.026
Cirrhosis, yes	9 (39)	180 (37)	0.630
HCC, yes	7 (30)	143 (30)	0.140
LSM, kPa	13 [7-28]	11 [6-21]	0.370
PNPLA3 p.I148M, M/M	8 (35)	135 (28)	0.110
**Liver biopsy**			
n=	10 (43)	157 (32)	
Steatosis, grade	2 [2-3]	2 [1-3]	0.270
Lobular inflammation, grade	1 [1-2]	1 [1-1]	0.830
Ballooning, grade	1 [1-2]	1 [0-1]	0.016
Fibrosis, stage	3 [1-4]	3 [1-4]	0.320

Data are shown as n (%), median [IQR]. BMI: body mass index; HDL: high density lipoprotein; LDL: low density lipoprotein; PLTs: platelets; FIB-4: fibrosis-4 index; LSM: liver stiffness measurement. ^ Available in 294 patients.
* At logistic regression models adjusted for age, sex, BMI, and PRS-5 for liver outcomes.

Supplementary Table 4. Enrichment of *APOB* variants in cases with severe MASLD vs. healthy controls in the clinical cohort.

Selection criteria	*APOB* variants in cases	*APOB* variants in controls	OR [95% C.I.]	p. (GLM)	p. (SKAT)
Definition A	25	2	13.8<br>[2.7 - 70.7]	0.002	0.004
Definition B	13	0	21.5<br>[1.9-236]	0.012	0.007
LoF	8	0	14.2<br>[0.8-247.6]	0.070	0.270

Different criteria to define likely pathogenic *APOB* variants in cases (n=510) and controls (n=261) were tested. OR are obtained through the logistic regression model, correcting for age, sex, and total cholesterol, and by burden test (SKAT test), that was applied correcting for age, sex, total cholesterols and BMI.

LoF: loss of function. OR: Odds Ratio

Supplementary Table 5. Impact of rare *APOB* variants on liver outcomes in the Milan case-control cohort.

	**APOB (definition D), n=18**	**APOB (LoF), n=7**	**ApoB100 (definition D), n=3**	**ApoB100 (LoF), n=2**	**ApoB48/100 (definition D), n=14**	**ApoB48/100 (LoF), n=5**
**Advanced MASLD**	6.24 [1.17-33.27]*	7.89 [0.45-138.62]	3.65 [0.19-70.94]	2.60 [0.12-54.41]	5.14 [0.95-27.77]*	5.76 [0.32-104.57]
**Cirrhosis**	3.42 [1.31-8.94]*	3.85 [0.86-17.29]	12.21 [0.63-237.23]	8.69 [0.42-181.64]	2.57 [0.94-7.06]	2.44 [0.48-12.41]
**HCC**	1.90 [0.72-4.98]	2.83 [0.69-11.54]	25.60 [1.31-498.1]*^	18.16 [0.87-380.24]	1.00 [0.3-3.32]	1.19 [0.19-7.64]

OR and 95% c.i. were calculated using SKAT-O. To uniform selection criteria withing the different cohort used in the study, in this case we considered exclusively LoF variants and missense variants predicted by at least 4 of this in silico prediction score to have high pathogenicity (definition D): REVEL ≥0.5, CADD ≥20, SIFT, PolyPhen, LRT, MutationTaster, M-CAP, AlphaMissense.

*Burden test p value smaller than 0.05; ^ SKATO p value smaller than 0.05.

Supplementary Table 6. Impact of *APOB* variant carriage on liver outcomes in the MVP cohort

	Population	Isoform	MAC case	MAC control	Pvalue	OR	SE
**LoF**							
Cirrhosis	African (949 vs. 22,505)	ApoB	2	19	0.173	4.74	1.14
		ApoB100	1	14	0.619	1.91	1.31
		ApoB48/100	1	5	0.056	89.58	2.35
	European (1971 vs.68,603))	ApoB	7	67	**0.004**	3.83	0.39
		ApoB100	3	41	0.111	4.26	0.91
		ApoB48/100	4	26	**0.005**	5.84	0.53
HCC	African (191 vs. 23,604)	ApoB	2	20	**0.002**	18.33	0.71
		ApoB100	1	15	**0.025**	14.65	0.92
		ApoB48/100	1	5	**0.006**	37.83	1.01
	European (326 vs. 70,704)	ApoB	5	71	**8.1E-05**	15.05	0.50
		ApoB100	2	43	0.081	47.02	2.20
		ApoB48/100	3	28	**3.1E-05**	35.17	0.61
**Deleterious missense+LoF**							
Cirrhosis	African (949 vs. 22,505)	ApoB	5	118	0.931	1.04	0.47
		ApoB100	2	65	0.646	0.75	0.62
		ApoB48/100	3	53	0.510	1.60	0.71
	European (1971 vs.68,603))	ApoB	10	172	**0.038**	2.09	0.32
		ApoB100	5	85	0.105	2.85	0.65
		ApoB48/100	5	87	0.153	2.43	0.62
HCC	African (191 vs. 23,604)	ApoB	3	123	**0.025**	4.56	0.55
		ApoB100	1	68	0.407	3.48	1.50
		ApoB48/100	2	55	**0.017**	7.72	0.67
	European (326 vs. 70,704)	ApoB	5	180	**0.005**	5.60	0.49
		ApoB100	2	89	0.390	3.70	1.52
		ApoB48/100	3	91	**0.002**	9.33	0.56

Risk of carriage of LoF ApoB variants in the MVP with Cirrhosis or HCC(n=70,575 European and 23,454 African Americans). P-values are two-tailed, uncorrected for multiple-testing and were calculated from burden tests using logistic regression with Firth's correction as implemented in REGENIE. The upper panel refers respectively to LoF affecting ApoB, ApoB48/100 or exclusively ApoB100, as specified in the "Isoform" column. The lower panel refers to deleterious missense variants and LoF. Deleterious missense variants are those predicted as harmful by AlphaMissense. OR: odds ratio; MAC: minor allele count.

Supplementary Table 7. Impact of *APOB* variant carriage on liver outcomes in the UK Biobank study.

	Isoform	MAC case	MAC control	Pvalue	OR	SE
**LoF**						
Cirrhosis (414,302 vs. 3,355)	ApoB	19	1094	**0.0014**	2.30	0.26
	ApoB100	8	295	**0.0007**	4.27	0.43
	ApoB48/100	11	799	0.082	1.98	0.39
HCC (417,180 vs. 477)	ApoB	5	1108	**0.007**	4.17	0.53
	ApoB100	0	303	0.596	0.37	1.89
	ApoB48/100	5	805	**0.002**	5.33	0.55
**Deleterious missense+LoF**						
Cirrhosis (414,302 vs. 3,355)	ApoB	25	1853	**0.0097**	1.76	0.21
	ApoB100	10	700	**0.043**	2.02	0.35
	ApoB48/100	15	1154	0.066	1.82	0.33
HCC (417,180 vs. 477)	ApoB	5	1873	0.068	3.38	0.67
	ApoB100	0	710	0.381	0.37	1.14
	ApoB48/100	5	1164	**0.012**	3.68	0.52

Risk of carriage of LoF ApoB variants in 417,657 Europeans from the UK Biobank. P-values are two-tailed, uncorrected for multiple-testing and were calculated from burden tests using logistic regression with Firth's correction as implemented in REGENIE. The upper panel refers to LoF and the lower panel refers to Deleterious variants. The upper panel refers respectively to LoF affecting ApoB, ApoB48/100 or exclusively ApoB100, as specified in the "Isoform" column. Similarly, the lower panel refers to deleterious missense variants and LoF. Deleterious missense variants are those predicted as harmful by AlphaMissense. The analysis was adjusted for age, sex, age×sex, age^2×sex, age^2, smoking, alcohol consumption, BMI, statin use. OR: odds ratio; MAC: minor allele count.

Supplementary Table 8. Impact of rare *APOB* LoF variants on hepatic and cardiometabolic phenotypes in the UKBB (n=417,657).

Phenotype	Isoform	Beta (SE)	Pvalue	n	n (case)	MAC case	MAC control
**LDL**	ApoB	-1.12 (0.03)	**0**	394062			
	ApoB100	-2.13 (0.06)	**0**				
	ApoB48/100	-0.68 (0.05)	**2.45 E-106**				
**HDL**	ApoB	0.17 (0.02)	**7.02 E-13**	361323			
	ApoB100	0.31 (0.05)	**1.54 E-11**				
	ApoB48/100	0.12 (0.03)	**1.84 E-05**				
**Triglycerides**	ApoB	-0.68 (0.03)	**4.73 E-143**	394489			
	ApoB100	-1.34 (0.05)	**4.18 E-149**				
	ApoB48/100	-0.44 (0.03)	**3.98 E-44**				
**Coronary artery disease**	ApoB	-0.30 (0.10)	**0.003**	413643	54965	122	980
	ApoB100	-0.69 (0.21)	**0.001**			24	272
	ApoB48/100	-0.17 (0.11)	0.123			98	708
**Heart failure**	ApoB	-0.14 (0.17)	0.408	413643	14561	34	1068
	ApoB100	-0.11 (0.33)	0.728			9	287
	ApoB48/100	-0.15 (0.19)	0.450			25	781
**ALT**	ApoB	0.21 (0.03)	**1.17 E-14**	394661			
	ApoB100	0.40 (0.05)	**3.27 E-15**				
	ApoB48/100	0.25 (0.10)	**2.44 E-05**				
**PDFF**	ApoB	0.40 (0.09)	**3.98 E-06**	33508			
	ApoB100	0.94 (0.19)	**4.83 E-07**				
	ApoB48/100	0.25 (0.10)	**0.001**				
**Chronic kidney failure**	ApoB	0.10 (0.15)	0.517	413643	16959	50	1052
	ApoB100	0.57 (0.30)	0.060			18	278
	ApoB48/100	-0.07 (0.18)	0.717			32	774
**Glucose**	ApoB	0.06 (0.03)	**0.042**	361062			
	ApoB100	0.11 (0.06)	0.059				
	ApoB48/100	0.04 (0.04)	0.221				
**HbA1c**	ApoB	0.15 (0.03)	**8.06 E-09**	394898			
	ApoB100	0.24 (0.05)	**2.73 E-06**				
	ApoB48/100	0.12 (0.03)	**9.82 E-05**				
**Diabetes**	ApoB	0.40 (0.11)	**0.0004**	413643	38385	117	985
	ApoB100	0.81 (0.21)	**0.00011**			35	261
	ApoB48/100	0.26 (0.14)	0.057			82	724

**C-reactive protein**	ApoB	0.11 (0.03)	**3.44 E-05**	393958			
	ApoB100	0.18 (0.05)	**0.0003**				
	ApoB48/100	0.08 (0.03)	**0.0076**				
**BMI**	ApoB	0.02 (0.03)	0.393	413643			
	ApoB100	0.06 (0.06)	0.275				
	ApoB48/100	0.01 (0.03)	0.736				

P-values are two-tailed, uncorrected for multiple-testing and were calculated from burden tests using either logistic regression with Firth's correction or linear regression as implemented in REGENIE. The analysis was adjusted for age, sex, age×sex, age^2×sex, age^2, smoking, alcohol consumption, BMI, statin use (except for cardiovascular outcomes), with additional adjustments for diabetes and hypertension for coronary artery disease. BMI: body mass index; HBA1C: glycated hemoglobin; HDL-C: high density lipoprotein cholesterol; LDL: low density lipoprotein cholesterol; PDFF: proton density fat fraction.

Supplementary Table 9. Association of APOB variants with statin use in the UKBB cohort.

Isoform	Carriers	NonCarriers	P	OR
*APOB*_LoF	139 (12.48%)	79,203 (18.99%)	8.41E-09	0.608
APOB_LoF/damaging	257 (13.68%)	79,085 (18.99%)	1.37E-09	0.676
ApoB100_LoF	12 (3.96%)	79,330 (18.98%)	1.07E-14	0.176
ApoB100_ LoF/damaging	77 (10.85%)	79,265 (18.98%)	5.53E-09	0.519
ApoB48/100_LoF	127 (15.66%)	79,215 (18.98%)	0.0154	0.792
ApoB48/100_ LoF/damaging	180 (15.38%)	79,162 (18.98%)	0.0015	0.776

P-values are two-tailed, uncorrected for multiple-testing and were calculated from burden tests using either logistic regression with Firth's correction or linear regression as implemented in REGENIE. The analysis was adjusted for age, sex, age×sex, age^2×sex, age^2, smoking, alcohol consumption, BMI.

Supplementary table 10. Impact of carriage of APOB variants on liver outcome in the MVP

	Isoform	A1FREQ	N	p value	OR	SE
**LoF**						
Cirrhosis	ApoB	0.0005	65124	0.009	3.28	0.396
	ApoB100	0.0003	65124	0.207	2.88	0.838
	ApoB48/100	0.0002	65124	0.009	5.22	0.537
HCC	ApoB	0.0005	65598	6.3583E-05	11.68	0.473
	ApoB100	0.0003	65598	0.022	7.26	0.699
	ApoB48/100	0.0002	65598	0.0001	23.56	0.615
**Deleterious missense+LoF**						
Cirrhosis	ApoB	0.0013	65124	0.059	2.26	0.432
	ApoB100	0.0006	65124	0.171	2.32	0.615
	ApoB48/100	0.0007	65124	0.195	2.19	0.607
HCC	ApoB	0.001	65598	0.0021	5.52	0.447
	ApoB100	0.0006	65598	0.059	12.23	1.328
	ApoB48/100	0.0006	65598	0.005	7.734	0.562

Risk of carriage of LoF ApoB variants in the MVP with Cirrhosis or HCC after correction for statin use. P-values are two-tailed, uncorrected for multiple-testing and were calculated from burden tests using logistic regression with Firth's correction as implemented in REGENIE. The upper panel refers respectively to LoF affecting ApoB, ApoB48/100 or exclusively ApoB100, as specified in the "Isoform" column. The lower panel refers to deleterious missense variants and LoF. Deleterious missense variants are those predicted as harmful by AlphaMissense. OR: odds ratio;

Supplementary Table 11. Protein quantitative trait loci (pQTL) summary stat for proteomic analysis of the impact of LoF in *APOB* (upper panel), and after stratification for variants affecting both ApoB48/B100 (middle panel) and specifically ApoB100 (lower panel) among ~3000 plasma proteins in individuals included the UKBB.

	Protein	Beta	SE	P cauchy FDR
ApoB	FGFBP1;Fibroblast growth factor-binding protein 1	-0.609	0.091	1.07E-07
	PCSK9;Proprotein convertase subtilisin/kexin type 9	-0.597	0.091	1.53E-07
	LDLR;Low-density lipoprotein receptor	-0.565	0.088	1.80E-07
	PLA2G10;Group 10 secretory phospholipase A2	0.595	0.093	1.80E-07
	CTSO;Cathepsin O	0.529	0.091	1.888E-06
	CES1;Liver carboxylesterase 1	0.495	0.088	1.88E-06
	KRT18;Keratin, type I cytoskeletal 18	0.454	0.085	6.75E-05
	IGFBPL1;Insulin-like growth factor-binding protein-like 1	0.454	0.089	0.00018
	PON2;Serum paraoxonase/arylesterase 2	0.458	0.094	0.00018
	GUSB;Beta-glucuronidase	0.399	0.085	0.00018
	HSD11B1;Corticosteroid 11-beta-dehydrogenase isozyme 1	0.365	0.091	0.0013
	HNMT;Histamine N-methyltransferase	0.388	0.088	0.0046
	IGSF3;Immunoglobulin superfamily member 3	0.378	0.088	0.0048
	PSAP;Prosaposin	0.422	0.097	0.0049
	CCL3;C-C motif chemokine 3	0.378	0.088	0.0056
	ULBP2;UL16-binding protein 2	0.386	0.094	0.013
	APOA2;Apolipoprotein A-II	0.391	0.097	0.014
	MET;Hepatocyte growth factor receptor	-0.359	0.090	0.021
	FTCD;Formimidoyltransferase-cyclodeaminase	0.360	0.092	0.022
	DLL1;Delta-like protein 1	0.292	0.091	0.023
	CD274;Programmed cell death 1 ligand 1	0.366	0.094	0.024
	TNFRSF10B;Tumor necrosis factor receptor superfamily member 10B	0.293	0.085	0.025
	ADH1B;All-trans-retinol dehydrogenase	0.286	0.094	0.026
	CDH2;Cadherin-2	0.307	0.085	0.028
	FSTL3;Follistatin-related protein 3	0.307	0.083	0.029
	EPHA2;Ephrin type-A receptor 2	0.334	0.090	0.032
	PLA2G15;Phospholipase A2 group XV	0.318	0.093	0.032
	TNFRSF11B;Tumor necrosis factor receptor superfamily member 11B	0.288	0.087	0.034
	RBP5;Retinol-binding protein 5	0.288	0.085	0.035
	NECTIN2;Nectin-2	0.346	0.094	0.037
	CD38;ADP-ribosyl cyclase/cyclic ADP-ribose hydrolase 1	0.252	0.077	0.037
	TNFRSF1A;Tumor necrosis factor receptor superfamily member 1A	0.3017	0.085	0.042
	NFASC;Neurofascin	0.310	0.088	0.042
	ASS1;Argininosuccinate synthase	0.318	0.097	0.043
	LBP;Lipopolysaccharide-binding protein	0.241	0.094	0.045
	OSCAR;Osteoclast-associated immunoglobulin-like receptor	0.329	0.094	0.045
	BPIFA2;BPI fold-containing family A member 2	0.359	0.100	0.049
ApoB48/100	IGFBPL1;Insulin-like growth factor-binding protein-like 1	0.502	0.109	0.00054

	FSTL3;Follistatin-related protein 3	0.420	0.102	0.011
	CTSO;Cathepsin O	0.479	0.112	0.019
	NECTIN2;Nectin-2	0.485	0.115	0.019
	KRT18;Keratin, type I cytoskeletal 18	0.437	0.104	0.023
	GUSB;Beta-glucuronidase	0.379	0.104	0.023
	HNMT;Histamine N-methyltransferase	0.427	0.108	0.035
	PON2;Serum paraoxonase/arylesterase 2	0.346	0.116	0.035
	CCL3;C-C motif chemokine 3	0.421	0.108	0.049
	USP47;Ubiquitin carboxyl-terminal hydrolase 47	0.502	0.129	0.049
	FGFBP1;Fibroblast growth factor-binding protein 1	-0.369	0.112	0.049
ApoB100	PCSK9;Proprotein convertase subtilisin/kexin type 9	-1.188	0.157	2.17E-10
	LDLR;Low-density lipoprotein receptor	-1.092	0.150	1.11E-09
	FGFBP1;Fibroblast growth factor-binding protein 1	-1.094	0.159	1.411E-08
	PLA2G10;Group 10 secretory phospholipase A2	1.051	0.159	7.39E-08
	CES1;Liver carboxylesterase 1	0.987	0.151	8.49E-08
	CTSO;Cathepsin O	0.623	0.155	0.011
	PON2;Serum paraoxonase/arylesterase 2	0.674	0.162	0.025

Only statistically significant differences (P_{Cauchy} FDR <0.05) are included.

ADH1B:All-trans-retinol dehydrogenase; APOA2:Apolipoprotein A-II; ASS1:Argininosuccinate synthase; BPIFA2:BPI fold-containing family A member 2; CCL3: C-C motif chemokine 3

CD274:Programmed cell death 1 ligand 1; CD38:ADP-ribosyl cyclase/cyclic ADP-ribose hydrolase 1; CDH2:Cadherin-2; CES1:Liver carboxylesterase 1; CTSO:Cathepsin O; DLL1:Delta-like protein 1; EPHA2:Ephrin type-A receptor 2; FGFBP1:Fibroblast growth factor-binding protein 1; FSTL3:Follistatin-related protein 3; FTCD:Formimidoyltransferase-cyclodeaminase; GUSB:Beta-glucuronidase; HNMT:Histamine N-methyltransferase; HSD11B1:Corticosteroid 11-beta-dehydrogenase isozyme 1; IGFBPL1:Insulin-like growth factor-binding protein-like 1; IGSF3:Immunoglobulin superfamily member 3; KRT18:Keratin, type I cytoskeletal 18; LBP:Lipopolysaccharide-binding protein; LDLR:Low-density lipoprotein receptor; MET:Hepatocyte growth factor receptor; NECTIN2:Nectin-2; NFASC:Neurofascin; OSCAR:Osteoclast-associated immunoglobulin-like receptor; PCSK9:Proprotein convertase subtilisin/kexin type 9; PLA2G10:Group 10 secretory phospholipase A2; PLA2G15:Phospholipase A2 group XV; PON2:Serum paraoxonase/arylesterase 2; PSAP:Prosaposin; RBP5:Retinol-binding protein 5; TNFRSF10B:Tumor necrosis factor receptor superfamily member 10B; TNFRSF11B:Tumor necrosis factor receptor superfamily member 11B; TNFRSF1A:Tumor necrosis factor receptor superfamily member 1A; LBP2:UL16-binding protein 2; USP47:Ubiquitin carboxyl-terminal hydrolase 47.

Supplementary references

1. Marchetti A, et al. Impact of clonal hematopoiesis of indeterminate potential on hepatocellular carcinoma in individuals with steatotic liver disease. *Hepatology.* 2024;80(4):816–27.
2. Rinella ME, et al. A multisociety Delphi consensus statement on new fatty liver disease nomenclature. *Journal of Hepatology.* 2023;79(6):1542–56.
3. EASL–EORTC Clinical Practice Guidelines: Management of hepatocellular carcinoma. *Journal of Hepatology.* 2012;56(4):908–43.
4. Berzigotti A, et al. EASL Clinical Practice Guidelines on non-invasive tests for evaluation of liver disease severity and prognosis – 2021 update. *Journal of Hepatology.* 2021;75(3):659–89.
5. Pelusi S, et al. Rare Pathogenic Variants Predispose to Hepatocellular Carcinoma in Nonalcoholic Fatty Liver Disease. *Scientific Reports.* 2019;9(1).
6. Baselli GA, et al. Rare ATG7 genetic variants predispose patients to severe fatty liver disease. *Journal of Hepatology.* 2022;77(3):596–606.
7. Van Der Auwera GA, et al. From FastQ Data to High-Confidence Variant Calls: The Genome Analysis Toolkit Best Practices Pipeline. *Current Protocols in Bioinformatics.* 2013;43(1).
8. Li H, Handsaker B,et al. The Sequence Alignment/Map format and SAMtools. *Bioinformatics.* 2009;25(16):2078–9.
9. Li H, and Durbin R. Fast and accurate long-read alignment with Burrows–Wheeler transform. *Bioinformatics.* 2010;26(5):589–95.
10. Pedersen BS, and Quinlan AR. Mosdepth: quick coverage calculation for genomes and exomes. *Bioinformatics.* 2018;34(5):867–8.
11. Quinlan AR, and Hall IM. BEDTools: a flexible suite of utilities for comparing genomic features. *Bioinformatics.* 2010;26(6):841–2.
12. Danecek P, and McCarthy SA. BCFtools/csq: haplotype-aware variant consequences. *Bioinformatics.* 2017;33(13):2037–9.
13. McLaren W, et al. The Ensembl Variant Effect Predictor. *Genome Biology.* 2016;17(1).
14. Pelusi S, et al. Prevalence and Determinants of Liver Disease in Relatives of Italian Patients With Advanced MASLD. *Clinical Gastroenterology and Hepatology.* 2024;22(11):2231–9.e4.
15. Jamialahmadi O, et al. Partitioned polygenic risk scores identify distinct types of metabolic dysfunction-associated steatotic liver disease. *Nature Medicine.* 2024;30(12):3614–23.

16. Julkunen H, et al. Atlas of plasma NMR biomarkers for health and disease in 118,461 individuals from the UK Biobank. *Nature Communications.* 2023;14(1).

17. Sun BB, et al. Plasma proteomic associations with genetics and health in the UK Biobank. *Nature.* 2023;622(7982):329–38.

18. Liu Y, et al.. ACAT: A Fast and Powerful p Value Combination Method for Rare-Variant Analysis in Sequencing Studies. *The American Journal of Human Genetics.* 2019;104(3):410–21.

19. Mbatchou J, et al. Computationally efficient whole-genome regression for quantitative and binary traits. *Nature Genetics.* 2021;53(7):1097–103.

20. Mbatchou J, et al. Computationally efficient whole-genome regression for quantitative and binary traits. *Nat Genet.* 2021;53(7):1097–103.

21. Willer CJ, et al. METAL: fast and efficient meta-analysis of genomewide association scans. *Bioinformatics.* 2010;26(17):2190–1.

22. Crudele L, et al. Low HDL-cholesterol levels predict hepatocellular carcinoma development in individuals with liver fibrosis. *JHEP Reports.* 2023;5(1):100627.

23. Lee H-Y, Net al. Mitochondrial Metabolic Signatures in Hepatocellular Carcinoma. *Cells.* 2021;10(8):1901.

24. Luukkonen PK, et al. The PNPLA3 I148M variant increases ketogenesis and decreases hepatic de novo lipogenesis and mitochondrial function in humans. *Cell Metabolism.* 2023;35(11):1887–96.e5.

25. Valenti L, and Romeo S. Editorial: new insights into the relationship between the intestine and non-alcoholic fatty liver—is “fatty gut” involved in disease progression? *Alimentary Pharmacology & Therapeutics.* 2017;46(3):377–8.
