# Supplementary material for: Carriage of rare APOB variants predisposes to severe steatotic liver disease and hepatocellular carcinoma
Source: J Clin Invest. 2026 Feb 10;136(8):e201762. doi: 10.1172/JCI201762 (PMC13078886; doi:10.1172/JCI201762)
Supplement: ICMJE disclosure forms [file jci-136-201762-s106.pdf]

ICMJE DISCLOSURE FORM

**Date:**	12/10/2025
**Your Name:**	Luca VC Valenti
**Manuscript Title:**	***Carriage of rare APOB variants predisposes to severe steatotic liver disease and hepatocellular carcinoma***
**Manuscript Number (if known):**	201762-JCI-CRPH-1

In the interest of transparency, we ask you to disclose all relationships/activities/interests listed below that are related to the content of your manuscript. "Related" means any relation with for-profit or not-for-profit third parties whose interests may be affected by the content of the manuscript. Disclosure represents a commitment to transparency and does not necessarily indicate a bias. If you are in doubt about whether to list a relationship/activity/interest, it is preferable that you do so.

The author's relationships/activities/interests should be defined broadly. For example, if your manuscript pertains to the epidemiology of hypertension, you should declare all relationships with manufacturers of antihypertensive medication, even if that medication is not mentioned in the manuscript.

In item #1 below, report all support for the work reported in this manuscript without time limit. For all other items, the time frame for disclosure is the past 36 months.

		**Name all entities with whom you have this relationship or indicate none (add rows as needed)**	**Specifications/Comments (e.g., if payments were made to you or to your institution)**
		**Time frame: Since the initial planning of the work**	
**1**	All support for the present manuscript (e.g., funding, provision of study materials, medical writing, article processing charges, etc.) **No time limit for this item.**	☐ **None**	
		NIH since MV received funding via R01DK134575 from the National Institute for Diabetes and Digestive and Kidney Diseases (MV); Italian Ministry of Health (Ministero della Salute), Ricerca Finalizzata 2021 RF-2021-12373889, Italian Ministry of Health, Ricerca Finalizzata PNRR 2022 "RATIONAL" PNRR-MAD-2022-12375656 (LV); Italian Ministry of Health (Ministero della Salute), Fondazione IRCCS Ca' Granda Ospedale Maggiore Policlinico, Ricerca Corrente (DP); The European Union, H2020-ICT-2018-20/H2020-ICT-2020-2 programme "Photonics" under grant agreement No. 101016726 - REVEAL (LV). The European Union, HORIZON-MISS-2021-CANCER-02-03 programme "Genial" under grant agreement "101096312" (LV, SP); Italian Ministry of Research (MUR) PNRR – M4 - C2	Funding provided to institution.

		“National Center for Gene Therapy and Drugs based on RNA Technology” CN3, Spoke 4 “ASSET” (LV); PRIN 2022 MUR: “Disentangling genetic, epigenetic and hormonal regulation of Fe/heme metabolism in the gender-specific nature of NAFLD (DEFENDER)”; Bando Ricerca Corrente and Piano Nazionale Complementare Ecosistema Innovativo della Salute - Hub Life Science-Diagnostica Avanzata (HLS-DA)’ - PNC-E3-2022-23683266 - ‘INNOVA’ (LV); Department of Pathophysiology and Transplantation, University of Milan, is funded by the Italian Ministry of Education and Research (MUR): Dipartimenti di Eccellenza Program 2023 to 2027) (LV); VA Merit Funding BX003362: “Genetics of Cardiometabolic Diseases in the VA Population” from the VA Office of R&D, Veterans Health Administration, USA (KMC, PST); The Swedish Cancerfonden (22 2270 Pj), the Swedish Research Council (Vetenskapsradet (VR), 2023-02079), the Swedish state under the Agreement between the Swedish government and the county councils (the ALF agreement, ALFGBG-965360), the Swedish Heart Lung Foundation (20220334), the Novonordisk Distinguished Investigator Grant - Endocrinology and Metabolism (NNF23OC0082114), the Novonordisk Project grants in Endocrinology and Metabolism (NNF24OC0091535) (SR).	
**Time frame: past 36 months**			
**2**	Grants or contracts from any entity (if not indicated in item #1 above).	X **None**	
**3**	Royalties or licenses	X **None**	

4	Consulting fees	☐ **None**<br><br>Novo Nordisk, Pfizer, Boehringer Ingelheim, Resalis, Almac. — Consulting fees paid to the author.
5	Payment or honoraria for lectures, presentations, speakers bureaus, manuscript writing or educational events	☐ **None**<br><br>Viatris, Novo Nordisk, GSK. — Speaking fees paid to the author.
6	Payment for expert testimony	x **None**
7	Support for attending meetings and/or travel	x **None**
8	Patents planned, issued or pending	x **None**
9	Participation on a Data Safety Monitoring Board or Advisory Board	x **None**
10	Leadership or fiduciary role in other board, society, committee or advocacy group, paid or unpaid	x **None**

**11**	Stock or stock options	x **None**
**12**	Receipt of equipment, materials, drugs, medical writing, gifts or other services	x **None**
**13**	Other financial or non-financial interests	x **None**

Please place an "X" next to the following statement to indicate your agreement:

x I certify that I have answered every question and have not altered the wording of any of the questions on this form.

ICMJE DISCLOSURE FORM

Date: 12/10/2025

Your Name: Matteo Mureddu

Manuscript Title: ***Carriage of rare APOB variants predisposes to severe steatotic liver disease and hepatocellular carcinoma***

Manuscript Number (if known): 201762-JCI-CRPH-1

In the interest of transparency, we ask you to disclose all relationships/activities/interests listed below that are related to the content of your manuscript. "Related" means any relation with for-profit or not-for-profit third parties whose interests may be affected by the content of the manuscript. Disclosure represents a commitment to transparency and does not necessarily indicate a bias. If you are in doubt about whether to list a relationship/activity/interest, it is preferable that you do so.

The author's relationships/activities/interests should be defined broadly. For example, if your manuscript pertains to the epidemiology of hypertension, you should declare all relationships with manufacturers of antihypertensive medication, even if that medication is not mentioned in the manuscript.

In item #1 below, report all support for the work reported in this manuscript without time limit. For all other items, the time frame for disclosure is the past 36 months.

		Name all entities with whom you have this relationship or indicate none (add rows as needed)	Specifications/Comments (e.g., if payments were made to you or to your institution)
		**Time frame: Since the initial planning of the work**	
**1**	All support for the present manuscript (e.g., funding, provision of study materials, medical writing, article processing charges, etc.) **No time limit for this item.**	☐ **None**	
		NIH since MV received funding via R01DK134575 from the National Institute for Diabetes and Digestive and Kidney Diseases (MV); Italian Ministry of Health (Ministero della Salute), Ricerca Finalizzata 2021 RF-2021-12373889, Italian Ministry of Health, Ricerca Finalizzata PNRR 2022 "RATIONAL" PNRR-MAD-2022-12375656 (LV); Italian Ministry of Health (Ministero della Salute), Fondazione IRCCS Ca' Granda Ospedale Maggiore Policlinico, Ricerca Corrente (DP); The European Union, H2020-ICT-2018-20/H2020-ICT-2020-2 programme "Photonics" under grant agreement No. 101016726 - REVEAL (LV). The European Union, HORIZON-MISS-2021-CANCER-02-03 programme "Genial" under grant agreement "101096312" (LV, SP); Italian Ministry of Research (MUR) PNRR – M4 - C2 "National Center for Gene Therapy and Drugs based on RNA Technology" CN3, Spoke 4 "ASSET" (LV); PRIN 2022 MUR: "Disentangling genetic, epigenetic and hormonal regulation of Fe/heme metabolism in the gender-specific nature of NAFLD (DEFENDER)"; Bando Ricerca Corrente and Piano Nazionale Complementare Ecosistema Innovativo della Salute - Hub Life Science-Diagnostica Avanzata (HLS-DA)' - PNC-E3-2022-23683266 - 'INNOVA' (LV); Department of Pathophysiology and Transplantation, University of Milan, is funded by the Italian Ministry of Education and Research (MUR): Dipartimenti di Eccellenza Program 2023 to 2027) (LV); VA Merit Funding BX003362: "Genetics of Cardiometabolic Diseases in the VA Population" from the VA Office of R&D, Veterans Health Administration, USA (KMC, PST); The Swedish Cancerfonden (22 2270 Pj), the Swedish Research Council (Vetenskapsradet (VR), 2023-02079), the Swedish state under the Agreement between the Swedish government and	Funding provided to institution.

		the county councils (the ALF agreement, ALFGBG-965360), the Swedish Heart Lung Foundation (20220334), the Novonordisk Distinguished Investigator Grant - Endocrinology and Metabolism (NNF23OC0082114), the Novonordisk Project grants in Endocrinology and Metabolism (NNF24OC0091535) (SR).	

Time frame: past 36 months

**2**	Grants or contracts from any entity (if not indicated in item #1 above).	X **None**	
**3**	Royalties or licenses	X **None**	
**4**	Consulting fees	X **None**	
**5**	Payment or honoraria for lectures, presentations, speakers bureaus, manuscript writing or educational events	X **None**	
**6**	Payment for expert testimony	x **None**	

**7**	Support for attending meetings and/or travel	x **None**
**8**	Patents planned, issued or pending	x **None**
**9**	Participation on a Data Safety Monitoring Board or Advisory Board	x **None**
**10**	Leadership or fiduciary role in other board, society, committee or advocacy group, paid or unpaid	x **None**
**11**	Stock or stock options	x **None**
**12**	Receipt of equipment, materials, drugs, medical writing, gifts or other services	x **None**
**13**	Other financial or non-financial interests	x **None**

Please place an "X" next to the following statement to indicate your agreement:

x I certify that I have answered every question and have not altered the wording of any of the questions on this form.

ICMJE DISCLOSURE FORM

**Date:**	12/10/2025
**Your Name:**	Serena Pelusi
**Manuscript Title:**	***Carriage of rare APOB variants predisposes to severe steatotic liver disease and hepatocellular carcinoma***
**Manuscript Number (if known):**	201762-JCI-CRPH-1

In the interest of transparency, we ask you to disclose all relationships/activities/interests listed below that are related to the content of your manuscript. "Related" means any relation with for-profit or not-for-profit third parties whose interests may be affected by the content of the manuscript. Disclosure represents a commitment to transparency and does not necessarily indicate a bias. If you are in doubt about whether to list a relationship/activity/interest, it is preferable that you do so.

The author's relationships/activities/interests should be defined broadly. For example, if your manuscript pertains to the epidemiology of hypertension, you should declare all relationships with manufacturers of antihypertensive medication, even if that medication is not mentioned in the manuscript.

In item #1 below, report all support for the work reported in this manuscript without time limit. For all other items, the time frame for disclosure is the past 36 months.

		**Name all entities with whom you have this relationship or indicate none (add rows as needed)**	**Specifications/Comments (e.g., if payments were made to you or to your institution)**
**Time frame: Since the initial planning of the work**			
**1**	All support for the present manuscript (e.g., funding, provision of study materials, medical writing, article processing charges, etc.) **No time limit for this item.**	☐ **None**	
		NIH since MV received funding via R01DK134575 from the National Institute for Diabetes and Digestive and Kidney Diseases (MV); Italian Ministry of Health (Ministero della Salute), Ricerca Finalizzata 2021 RF-2021-12373889, Italian Ministry of Health, Ricerca Finalizzata PNRR 2022 "RATIONAL" PNRR-MAD-2022-12375656 (LV); Italian Ministry of Health (Ministero della Salute), Fondazione IRCCS Ca' Granda Ospedale Maggiore Policlinico, Ricerca Corrente (DP); The European Union, H2020-ICT-2018-20/H2020-ICT-2020-2 programme "Photonics" under grant agreement No. 101016726 - REVEAL (LV). The European Union, HORIZON-MISS-2021-CANCER-02-03 programme "Genial" under grant agreement "101096312" (LV, SP); Italian Ministry of Research (MUR) PNRR – M4 - C2 "National Center for Gene Therapy and	Funding provided to institution.

		Drugs based on RNA Technology" CN3, Spoke 4 "ASSET" (LV); PRIN 2022 MUR: "Disentangling genetic, epigenetic and hormonal regulation of Fe/heme metabolism in the gender-specific nature of NAFLD (DEFENDER)"; Bando Ricerca Corrente and Piano Nazionale Complementare Ecosistema Innovativo della Salute - Hub Life Science-Diagnostica Avanzata (HLS-DA)' - PNC-E3-2022-23683266 - 'INNOVA' (LV); Department of Pathophysiology and Transplantation, University of Milan, is funded by the Italian Ministry of Education and Research (MUR): Dipartimenti di Eccellenza Program 2023 to 2027) (LV); VA Merit Funding BX003362: "Genetics of Cardiometabolic Diseases in the VA Population" from the VA Office of R&D, Veterans Health Administration, USA (KMC, PST); The Swedish Cancerfonden (22 2270 Pj), the Swedish Research Council (Vetenskapsradet (VR), 2023-02079), the Swedish state under the Agreement between the Swedish government and the county councils (the ALF agreement, ALFGBG-965360), the Swedish Heart Lung Foundation (20220334), the Novonordisk Distinguished Investigator Grant - Endocrinology and Metabolism (NNF23OC0082114), the Novonordisk Project grants in Endocrinology and Metabolism (NNF24OC0091535) (SR).	

Time frame: past 36 months

2	Grants or contracts from any entity (if not indicated in item #1 above).	X **None**	
3	Royalties or licenses	X **None**	

**4**	Consulting fees	X **None**
**5**	Payment or honoraria for lectures, presentations, speakers bureaus, manuscript writing or educational events	X **None**
**6**	Payment for expert testimony	x **None**
**7**	Support for attending meetings and/or travel	x **None**
**8**	Patents planned, issued or pending	x **None**
**9**	Participation on a Data Safety Monitoring Board or Advisory Board	x **None**
**10**	Leadership or fiduciary role in other board, society, committee or advocacy group, paid or unpaid	x **None**

11	Stock or stock options	x **None**
12	Receipt of equipment, materials, drugs, medical writing, gifts or other services	x **None**
13	Other financial or non-financial interests	x **None**

Please place an "X" next to the following statement to indicate your agreement:

x I certify that I have answered every question and have not altered the wording of any of the questions on this form.

ICMJE DISCLOSURE FORM

Date: 12/10/2025

Your Name: Stefano Romeo

Manuscript Title: ***Carriage of rare APOB variants predisposes to severe steatotic liver disease and hepatocellular carcinoma***

Manuscript Number (if known): 201762-JCI-CRPH-1

In the interest of transparency, we ask you to disclose all relationships/activities/interests listed below that are related to the content of your manuscript. "Related" means any relation with for-profit or not-for-profit third parties whose interests may be affected by the content of the manuscript. Disclosure represents a commitment to transparency and does not necessarily indicate a bias. If you are in doubt about whether to list a relationship/activity/interest, it is preferable that you do so.

The author's relationships/activities/interests should be defined broadly. For example, if your manuscript pertains to the epidemiology of hypertension, you should declare all relationships with manufacturers of antihypertensive medication, even if that medication is not mentioned in the manuscript.

In item #1 below, report all support for the work reported in this manuscript without time limit. For all other items, the time frame for disclosure is the past 36 months.

		Name all entities with whom you have this relationship or indicate none (add rows as needed)	Specifications/Comments (e.g., if payments were made to you or to your institution)
**Time frame: Since the initial planning of the work**			
**1**	All support for the present manuscript (e.g., funding, provision of study materials, medical writing, article processing charges, etc.) **No time limit for this item.**	☐ **None**	
		NIH since MV received funding via R01DK134575 from the National Institute for Diabetes and Digestive and Kidney Diseases (MV); Italian Ministry of Health (Ministero della Salute), Ricerca Finalizzata 2021 RF-2021-12373889, Italian Ministry of Health, Ricerca Finalizzata PNRR 2022 "RATIONAL" PNRR-MAD-2022-12375656 (LV); Italian Ministry of Health (Ministero della Salute), Fondazione IRCCS Ca' Granda Ospedale Maggiore Policlinico, Ricerca Corrente (DP); The European Union, H2020-ICT-2018-20/H2020-ICT-2020-2 programme "Photonics" under grant agreement No. 101016726 - REVEAL (LV). The European Union, HORIZON-MISS-2021-CANCER-02-03 programme "Genial" under grant agreement "101096312" (LV, SP); Italian Ministry of Research (MUR) PNRR – M4 - C2 "National Center for Gene Therapy and Drugs based on RNA Technology" CN3, Spoke 4 "ASSET" (LV); PRIN 2022 MUR: "Disentangling genetic, epigenetic and hormonal regulation of Fe/heme metabolism in the gender-specific nature of NAFLD (DEFENDER)"; Bando Ricerca Corrente and Piano Nazionale Complementare Ecosistema Innovativo della Salute - Hub Life Science-Diagnostica Avanzata (HLS-DA)' - PNC-E3-2022-23683266 - 'INNOVA' (LV); Department of Pathophysiology and Transplantation, University of Milan, is funded by the Italian Ministry of Education and Research (MUR): Dipartimenti di Eccellenza Program 2023 to 2027) (LV); VA Merit Funding BX003362: "Genetics of Cardiometabolic Diseases in the VA Population" from the VA Office of R&D, Veterans Health Administration, USA (KMC, PST); The Swedish Cancerfonden (22 2270 Pj), the Swedish Research Council (Vetenskapsradet (VR), 2023-02079), the Swedish state under the Agreement between the Swedish government and the county councils (the ALF agreement,	Funding provided to institution.

		ALFGBG-965360), the Swedish Heart Lung Foundation (20220334), the Novonordisk Distinguished Investigator Grant - Endocrinology and Metabolism (NNF23OC0082114), the Novonordisk Project grants in Endocrinology and Metabolism (NNF24OC0091535) (SR).	
**Time frame: past 36 months**			
**2**	Grants or contracts from any entity (if not indicated in item #1 above).	X **None**	
**3**	Royalties or licenses	X **None**	
**4**	Consulting fees	X **None**	
**5**	Payment or honoraria for lectures, presentations, speakers bureaus, manuscript writing or educational events	X **None**	
**6**	Payment for expert testimony	x **None**	
**7**	Support for attending meetings and/or travel	x **None**	

8	Patents planned, issued or pending	x **None**
9	Participation on a Data Safety Monitoring Board or Advisory Board	x **None**
10	Leadership or fiduciary role in other board, society, committee or advocacy group, paid or unpaid	x **None**
11	Stock or stock options	x **None**
12	Receipt of equipment, materials, drugs, medical writing, gifts or other services	x **None**
13	Other financial or non-financial interests	x **None**

Please place an "X" next to the following statement to indicate your agreement:

x I certify that I have answered every question and have not altered the wording of any of the questions on this form.

ICMJE DISCLOSURE FORM

Date: 12/10/2025

Your Name: Vittoria Moretti

Manuscript Title: ***Carriage of rare APOB variants predisposes to severe steatotic liver disease and hepatocellular carcinoma***

**Manuscript Number (if known):**	201762-JCI-CRPH-1

In the interest of transparency, we ask you to disclose all relationships/activities/interests listed below that are related to the content of your manuscript. "Related" means any relation with for-profit or not-for-profit third parties whose interests may be affected by the content of the manuscript. Disclosure represents a commitment to transparency and does not necessarily indicate a bias. If you are in doubt about whether to list a relationship/activity/interest, it is preferable that you do so.

The author's relationships/activities/interests should be defined broadly. For example, if your manuscript pertains to the epidemiology of hypertension, you should declare all relationships with manufacturers of antihypertensive medication, even if that medication is not mentioned in the manuscript.

In item #1 below, report all support for the work reported in this manuscript without time limit. For all other items, the time frame for disclosure is the past 36 months.

		Name all entities with whom you have this relationship or indicate none (add rows as needed)	Specifications/Comments (e.g., if payments were made to you or to your institution)
**Time frame: Since the initial planning of the work**			
**1**	All support for the present manuscript (e.g., funding, provision of study materials, medical writing, article processing charges, etc.) **No time limit for this item.**	☐ **None**	
		NIH since MV received funding via R01DK134575 from the National Institute for Diabetes and Digestive and Kidney Diseases (MV); Italian Ministry of Health (Ministero della Salute), Ricerca Finalizzata 2021 RF-2021-12373889, Italian Ministry of Health, Ricerca Finalizzata PNRR 2022 "RATIONAL" PNRR-MAD-2022-12375656 (LV); Italian Ministry of Health (Ministero della Salute), Fondazione IRCCS Ca' Granda Ospedale Maggiore Policlinico, Ricerca Corrente (DP); The European Union, H2020-ICT-2018-20/H2020-ICT-2020-2 programme "Photonics" under grant agreement No. 101016726 - REVEAL (LV). The European Union, HORIZON-MISS-2021-CANCER-02-03 programme "Genial" under grant agreement "101096312" (LV, SP); Italian Ministry of Research (MUR) PNRR – M4 - C2 "National Center for Gene Therapy and Drugs based on RNA Technology" CN3, Spoke 4 "ASSET" (LV); PRIN 2022 MUR: "Disentangling genetic, epigenetic and hormonal regulation of Fe/heme metabolism in the gender-specific nature of NAFLD (DEFENDER)"; Bando Ricerca Corrente and Piano Nazionale Complementare Ecosistema Innovativo della Salute - Hub Life Science-Diagnostica Avanzata (HLS-DA)' - PNC-E3-2022-23683266 - 'INNOVA' (LV); Department of Pathophysiology and	Funding provided to institution.

		Name all entities with whom you have this relationship or indicate none (add rows as needed)	Specifications/Comments (e.g., if payments were made to you or to your institution)
		Transplantation, University of Milan, is funded by the Italian Ministry of Education and Research (MUR): Dipartimenti di Eccellenza Program 2023 to 2027) (LV); VA Merit Funding BX003362: "Genetics of Cardiometabolic Diseases in the VA Population" from the VA Office of R&D, Veterans Health Administration, USA (KMC, PST); The Swedish Cancerfonden (22 2270 Pj), the Swedish Research Council (Vetenskapsradet (VR), 2023-02079), the Swedish state under the Agreement between the Swedish government and the county councils (the ALF agreement, ALFGBG-965360), the Swedish Heart Lung Foundation (20220334), the Novonordisk Distinguished Investigator Grant - Endocrinology and Metabolism (NNF23OC0082114), the Novonordisk Project grants in Endocrinology and Metabolism (NNF24OC0091535) (SR).	
**Time frame: past 36 months**			
**2**	Grants or contracts from any entity (if not indicated in item #1 above).	X **None**	
**3**	Royalties or licenses	X **None**	
**4**	Consulting fees	X **None**	
**5**	Payment or honoraria for lectures, presentations, speakers bureaus,	X **None**	

		Name all entities with whom you have this relationship or indicate none (add rows as needed)	Specifications/Comments (e.g., if payments were made to you or to your institution)
	manuscript writing or educational events		
6	Payment for expert testimony	x None	
7	Support for attending meetings and/or travel	x None	
8	Patents planned, issued or pending	x None	
9	Participation on a Data Safety Monitoring Board or Advisory Board	x None	
10	Leadership or fiduciary role in other board, society, committee or advocacy group, paid or unpaid	x None	
11	Stock or stock options	x None	
12	Receipt of equipment, materials, drugs, medical writing, gifts or other services	x None	

		Name all entities with whom you have this relationship or indicate none (add rows as needed)	Specifications/Comments (e.g., if payments were made to you or to your institution)
13	Other financial or non-financial interests	x None	

Please place an "X" next to the following statement to indicate your agreement:

x I certify that I have answered every question and have not altered the wording of any of the questions on this form.

ICMJE DISCLOSURE FORM

Date: 12/10/2025

Your Name: Vincenzo La Mura

Manuscript Title: ***Carriage of rare APOB variants predisposes to severe steatotic liver disease and hepatocellular carcinoma***

Manuscript Number (if known): 201762-JCI-CRPH-1

In the interest of transparency, we ask you to disclose all relationships/activities/interests listed below that are related to the content of your manuscript. "Related" means any relation with for-profit or not-for-profit third parties whose interests may be affected by the content of the manuscript. Disclosure represents a commitment to transparency and does not necessarily indicate a bias. If you are in doubt about whether to list a relationship/activity/interest, it is preferable that you do so.

The author's relationships/activities/interests should be defined broadly. For example, if your manuscript pertains to the epidemiology of hypertension, you should declare all relationships with manufacturers of antihypertensive medication, even if that medication is not mentioned in the manuscript.

In item #1 below, report all support for the work reported in this manuscript without time limit. For all other items, the time frame for disclosure is the past 36 months.

		Name all entities with whom you have this relationship or indicate none (add rows as needed)	Specifications/Comments (e.g., if payments were made to you or to your institution)
**Time frame: Since the initial planning of the work**			
1	All support for the present manuscript (e.g., funding, provision of study materials, medical writing, article processing charges, etc.)	☐ None	
		NIH since MV received funding via R01DK134575 from the National Institute for Diabetes and Digestive and Kidney Diseases (MV); Italian Ministry of Health (Ministero della Salute), Ricerca Finalizzata 2021 RF-2021-12373889, Italian Ministry of Health, Ricerca	Funding provided to institution.

		Name all entities with whom you have this relationship or indicate none (add rows as needed)	Specifications/Comments (e.g., if payments were made to you or to your institution)
	**No time limit for this item.**	Finalizzata PNRR 2022 “RATIONAL” PNRR-MAD-2022-12375656 (LV); Italian Ministry of Health (Ministero della Salute), Fondazione IRCCS Ca’ Granda Ospedale Maggiore Policlinico, Ricerca Corrente (DP); The European Union, H2020-ICT-2018-20/H2020-ICT-2020-2 programme “Photonics” under grant agreement No. 101016726 - REVEAL (LV). The European Union, HORIZON-MISS-2021-CANCER-02-03 programme “Genial” under grant agreement “101096312” (LV, SP); Italian Ministry of Research (MUR) PNRR – M4 - C2 “National Center for Gene Therapy and Drugs based on RNA Technology” CN3, Spoke 4 “ASSET” (LV); PRIN 2022 MUR: “Disentangling genetic, epigenetic and hormonal regulation of Fe/heme metabolism in the gender-specific nature of NAFLD (DEFENDER)”; Bando Ricerca Corrente and Piano Nazionale Complementare Ecosistema Innovativo della Salute - Hub Life Science-Diagnostica Avanzata (HLS-DA)’ - PNC-E3-2022-23683266 - ‘INNOVA’ (LV); Department of Pathophysiology and Transplantation, University of Milan, is funded by the Italian Ministry of Education and Research (MUR): Dipartimenti di Eccellenza Program 2023 to 2027) (LV); VA Merit Funding BX003362: “Genetics of Cardiometabolic Diseases in the VA Population” from the VA Office of R&D, Veterans Health Administration, USA (KMC, PST); The Swedish Cancerfonden (22 2270 Pj), the Swedish Research Council (Vetenskapsradet (VR), 2023-02079), the Swedish state under the Agreement between the Swedish government and the county councils (the ALF agreement, ALFGBG-965360), the Swedish Heart Lung Foundation (20220334), the Novonordisk Distinguished Investigator Grant - Endocrinology and Metabolism (NNF23OC0082114), the Novonordisk Project grants in Endocrinology and Metabolism (NNF24OC0091535) (SR).	

Time frame: past 36 months

		Name all entities with whom you have this relationship or indicate none (add rows as needed)	Specifications/Comments (e.g., if payments were made to you or to your institution)
2	Grants or contracts from any entity (if not indicated in item #1 above).	X None	
3	Royalties or licenses	X None	
4	Consulting fees	X None	
5	Payment or honoraria for lectures, presentations, speakers bureaus, manuscript writing or educational events	X None	
6	Payment for expert testimony	x None	
7	Support for attending meetings and/or travel	x None	
8	Patents planned, issued or pending	x None	
9	Participation on a Data Safety	x None	

		Name all entities with whom you have this relationship or indicate none (add rows as needed)	Specifications/Comments (e.g., if payments were made to you or to your institution)
	Monitoring Board or Advisory Board		
10	Leadership or fiduciary role in other board, society, committee or advocacy group, paid or unpaid	x **None**	
11	Stock or stock options	x **None**	
12	Receipt of equipment, materials, drugs, medical writing, gifts or other services	x **None**	
13	Other financial or non-financial interests	x **None**	

Please place an "X" next to the following statement to indicate your agreement:

x I certify that I have answered every question and have not altered the wording of any of the questions on this form.

ICMJE DISCLOSURE FORM

**Date:**	12/10/2025
**Your Name:**	Umberto Vespasiani-Gentilucci
**Manuscript Title:**	***Carriage of rare APOB variants predisposes to severe steatotic liver disease and hepatocellular carcinoma***
**Manuscript Number (if known):**	201762-JCI-CRPH-1

In the interest of transparency, we ask you to disclose all relationships/activities/interests listed below that are related to the content of your manuscript. "Related" means any relation with for-profit or not-for-profit third parties whose interests may be affected by

the content of the manuscript. Disclosure represents a commitment to transparency and does not necessarily indicate a bias. If you are in doubt about whether to list a relationship/activity/interest, it is preferable that you do so.

The author's relationships/activities/interests should be defined broadly. For example, if your manuscript pertains to the epidemiology of hypertension, you should declare all relationships with manufacturers of antihypertensive medication, even if that medication is not mentioned in the manuscript.

In item #1 below, report all support for the work reported in this manuscript without time limit. For all other items, the time frame for disclosure is the past 36 months.

		Name all entities with whom you have this relationship or indicate none (add rows as needed)	Specifications/Comments (e.g., if payments were made to you or to your institution)
**Time frame: Since the initial planning of the work**			
**1**	All support for the present manuscript (e.g., funding, provision of study materials, medical writing, article processing charges, etc.) **No time limit for this item.**	☐ **None**	
		NIH since MV received funding via R01DK134575 from the National Institute for Diabetes and Digestive and Kidney Diseases (MV); Italian Ministry of Health (Ministero della Salute), Ricerca Finalizzata 2021 RF-2021-12373889, Italian Ministry of Health, Ricerca Finalizzata PNRR 2022 "RATIONAL" PNRR-MAD-2022-12375656 (LV); Italian Ministry of Health (Ministero della Salute), Fondazione IRCCS Ca' Granda Ospedale Maggiore Policlinico, Ricerca Corrente (DP); The European Union, H2020-ICT-2018-20/H2020-ICT-2020-2 programme "Photonics" under grant agreement No. 101016726 - REVEAL (LV). The European Union, HORIZON-MISS-2021-CANCER-02-03 programme "Genial" under grant agreement "101096312" (LV, SP); Italian Ministry of Research (MUR) PNRR – M4 - C2 "National Center for Gene Therapy and Drugs based on RNA Technology" CN3, Spoke 4 "ASSET" (LV); PRIN 2022 MUR: "Disentangling genetic, epigenetic and hormonal regulation of Fe/heme metabolism in the gender-specific nature of NAFLD (DEFENDER)"; Bando Ricerca Corrente and Piano Nazionale Complementare Ecosistema Innovativo della Salute - Hub Life Science-Diagnostica Avanzata (HLS-DA)' - PNC-E3-2022-23683266 - 'INNOVA' (LV); Department of Pathophysiology and Transplantation, University of Milan, is funded by the Italian Ministry of Education and Research (MUR): Dipartimenti di Eccellenza Program 2023	Funding provided to institution.

		Name all entities with whom you have this relationship or indicate none (add rows as needed)	Specifications/Comments (e.g., if payments were made to you or to your institution)
		to 2027) (LV); VA Merit Funding BX003362: "Genetics of Cardiometabolic Diseases in the VA Population" from the VA Office of R&D, Veterans Health Administration, USA (KMC, PST); The Swedish Cancerfonden (22 2270 Pj), the Swedish Research Council (Vetenskapsradet (VR), 2023-02079), the Swedish state under the Agreement between the Swedish government and the county councils (the ALF agreement, ALFGBG-965360), the Swedish Heart Lung Foundation (20220334), the Novonordisk Distinguished Investigator Grant - Endocrinology and Metabolism (NNF23OC0082114), the Novonordisk Project grants in Endocrinology and Metabolism (NNF24OC0091535) (SR).	
**Time frame: past 36 months**			
**2**	Grants or contracts from any entity (if not indicated in item #1 above).	X **None**	
**3**	Royalties or licenses	X **None**	
**4**	Consulting fees	X **None**	
**5**	Payment or honoraria for lectures, presentations, speakers bureaus, manuscript writing or educational events	X **None**	

		Name all entities with whom you have this relationship or indicate none (add rows as needed)	Specifications/Comments (e.g., if payments were made to you or to your institution)
6	Payment for expert testimony	x **None**	
7	Support for attending meetings and/or travel	x **None**	
8	Patents planned, issued or pending	x **None**	
9	Participation on a Data Safety Monitoring Board or Advisory Board	x **None**	
10	Leadership or fiduciary role in other board, society, committee or advocacy group, paid or unpaid	x **None**	
11	Stock or stock options	x **None**	
12	Receipt of equipment, materials, drugs, medical writing, gifts or other services	x **None**	
13	Other financial or non-financial interests	x **None**	

	Name all entities with whom you have this relationship or indicate none (add rows as needed)	Specifications/Comments (e.g., if payments were made to you or to your institution)

Please place an "X" next to the following statement to indicate your agreement:

X I certify that I have answered every question and have not altered the wording of any of the questions on this form.

ICMJE DISCLOSURE FORM

**Date:**	12/10/2025
**Your Name:**	Salvatore Petta
**Manuscript Title:**	***Carriage of rare APOB variants predisposes to severe steatotic liver disease and hepatocellular carcinoma***
**Manuscript Number (if known):**	201762-JCI-CRPH-1

In the interest of transparency, we ask you to disclose all relationships/activities/interests listed below that are related to the content of your manuscript. "Related" means any relation with for-profit or not-for-profit third parties whose interests may be affected by the content of the manuscript. Disclosure represents a commitment to transparency and does not necessarily indicate a bias. If you are in doubt about whether to list a relationship/activity/interest, it is preferable that you do so.

The author's relationships/activities/interests should be defined broadly. For example, if your manuscript pertains to the epidemiology of hypertension, you should declare all relationships with manufacturers of antihypertensive medication, even if that medication is not mentioned in the manuscript.

In item #1 below, report all support for the work reported in this manuscript without time limit. For all other items, the time frame for disclosure is the past 36 months.

		Name all entities with whom you have this relationship or indicate none (add rows as needed)	Specifications/Comments (e.g., if payments were made to you or to your institution)
**Time frame: Since the initial planning of the work**			
**1**	All support for the present manuscript (e.g., funding, provision of study materials, medical writing, article processing charges, etc.) **No time limit for this item.**	☐ **None**	
		NIH since MV received funding via R01DK134575 from the National Institute for Diabetes and Digestive and Kidney Diseases (MV); Italian Ministry of Health (Ministero della Salute), Ricerca Finalizzata 2021 RF-2021-12373889, Italian Ministry of Health, Ricerca Finalizzata PNRR 2022 "RATIONAL" PNRR-MAD-2022-12375656 (LV); Italian Ministry of Health (Ministero della Salute), Fondazione IRCCS Ca' Granda Ospedale Maggiore Policlinico, Ricerca Corrente (DP); The European Union, H2020-ICT-2018-20/H2020-ICT-2020-2	Funding provided to institution.

		Name all entities with whom you have this relationship or indicate none (add rows as needed)	Specifications/Comments (e.g., if payments were made to you or to your institution)
		programme "Photonics" under grant agreement No. 101016726 - REVEAL (LV). The European Union, HORIZON-MISS-2021-CANCER-02-03 programme "Genial" under grant agreement "101096312" (LV, SP); Italian Ministry of Research (MUR) PNRR – M4 - C2 "National Center for Gene Therapy and Drugs based on RNA Technology" CN3, Spoke 4 "ASSET" (LV); PRIN 2022 MUR: "Disentangling genetic, epigenetic and hormonal regulation of Fe/heme metabolism in the gender-specific nature of NAFLD (DEFENDER)"; Bando Ricerca Corrente and Piano Nazionale Complementare Ecosistema Innovativo della Salute - Hub Life Science-Diagnostica Avanzata (HLS-DA)' - PNC-E3-2022-23683266 - 'INNOVA' (LV); Department of Pathophysiology and Transplantation, University of Milan, is funded by the Italian Ministry of Education and Research (MUR): Dipartimenti di Eccellenza Program 2023 to 2027) (LV); VA Merit Funding BX003362: "Genetics of Cardiometabolic Diseases in the VA Population" from the VA Office of R&D, Veterans Health Administration, USA (KMC, PST); The Swedish Cancerfonden (22 2270 Pj), the Swedish Research Council (Vetenskapsradet (VR), 2023-02079), the Swedish state under the Agreement between the Swedish government and the county councils (the ALF agreement, ALFGBG-965360), the Swedish Heart Lung Foundation (20220334), the Novonordisk Distinguished Investigator Grant - Endocrinology and Metabolism (NNF23OC0082114), the Novonordisk Project grants in Endocrinology and Metabolism (NNF24OC0091535) (SR).	

Time frame: past 36 months

**2**	Grants or contracts from any entity (if not indicated in item #1 above).	X **None**	

		Name all entities with whom you have this relationship or indicate none (add rows as needed)	Specifications/Comments (e.g., if payments were made to you or to your institution)
**3**	Royalties or licenses	X **None**	
**4**	Consulting fees	X **None**	
**5**	Payment or honoraria for lectures, presentations, speakers bureaus, manuscript writing or educational events	X **None**	
**6**	Payment for expert testimony	x **None**	
**7**	Support for attending meetings and/or travel	x **None**	
**8**	Patents planned, issued or pending	x **None**	
**9**	Participation on a Data Safety Monitoring Board or Advisory Board	x **None**	
**10**	Leadership or fiduciary role in other board,	x **None**	

		Name all entities with whom you have this relationship or indicate none (add rows as needed)	Specifications/Comments (e.g., if payments were made to you or to your institution)
	society, committee or advocacy group, paid or unpaid		
11	Stock or stock options	x **None**	
12	Receipt of equipment, materials, drugs, medical writing, gifts or other services	x **None**	
13	Other financial or non-financial interests	x **None**	

Please place an "X" next to the following statement to indicate your agreement:

x I certify that I have answered every question and have not altered the wording of any of the questions on this form.

ICMJE DISCLOSURE FORM

**Date:**	12/10/2025
**Your Name:**	Saleh Alqahtani
**Manuscript Title:**	***Carriage of rare APOB variants predisposes to severe steatotic liver disease and hepatocellular carcinoma***
**Manuscript Number (if known):**	201762-JCI-CRPH-1

In the interest of transparency, we ask you to disclose all relationships/activities/interests listed below that are related to the content of your manuscript. "Related" means any relation with for-profit or not-for-profit third parties whose interests may be affected by the content of the manuscript. Disclosure represents a commitment to transparency and does not necessarily indicate a bias. If you are in doubt about whether to list a relationship/activity/interest, it is preferable that you do so.

The author's relationships/activities/interests should be defined broadly. For example, if your manuscript pertains to the epidemiology of hypertension, you should declare all relationships with manufacturers of antihypertensive medication, even if that medication is not mentioned in the manuscript.

In item #1 below, report all support for the work reported in this manuscript without time limit. For all other items, the time frame for disclosure is the past 36 months.

		Name all entities with whom you have this relationship or indicate none (add rows as needed)	Specifications/Comments (e.g., if payments were made to you or to your institution)
**Time frame: Since the initial planning of the work**			
1	All support for the present manuscript (e.g., funding, provision of study materials, medical writing, article processing charges, etc.) **No time limit for this item.**	☐ **None**	
		NIH since MV received funding via R01DK134575 from the National Institute for Diabetes and Digestive and Kidney Diseases (MV); Italian Ministry of Health (Ministero della Salute), Ricerca Finalizzata 2021 RF-2021-12373889, Italian Ministry of Health, Ricerca Finalizzata PNRR 2022 "RATIONAL" PNRR-MAD-2022-12375656 (LV); Italian Ministry of Health (Ministero della Salute), Fondazione IRCCS Ca' Granda Ospedale Maggiore Policlinico, Ricerca Corrente (DP); The European Union, H2020-ICT-2018-20/H2020-ICT-2020-2 programme "Photonics" under grant agreement No. 101016726 - REVEAL (LV). The European Union, HORIZON-MISS-2021-CANCER-02-03 programme "Genial" under grant agreement "101096312" (LV, SP); Italian Ministry of Research (MUR) PNRR – M4 - C2 "National Center for Gene Therapy and Drugs based on RNA Technology" CN3, Spoke 4 "ASSET" (LV); PRIN 2022 MUR: "Disentangling genetic, epigenetic and hormonal regulation of Fe/heme metabolism in the gender-specific nature of NAFLD (DEFENDER)"; Bando Ricerca Corrente and Piano Nazionale Complementare Ecosistema Innovativo della Salute - Hub Life Science-Diagnostica Avanzata (HLS-DA)' - PNC-E3-2022-23683266 - 'INNOVA' (LV); Department of Pathophysiology and Transplantation, University of Milan, is funded by the Italian Ministry of Education and Research (MUR): Dipartimenti di Eccellenza Program 2023 to 2027) (LV); VA Merit Funding BX003362: "Genetics of Cardiometabolic Diseases in the VA Population" from the VA Office of R&D, Veterans Health Administration, USA (KMC, PST); The Swedish Cancerfonden (22 2270 Pj), the Swedish Research Council	Funding provided to institution.

		Name all entities with whom you have this relationship or indicate none (add rows as needed)	Specifications/Comments (e.g., if payments were made to you or to your institution)
		(Vetenskapsradet (VR), 2023-02079), the Swedish state under the Agreement between the Swedish government and the county councils (the ALF agreement, ALFGBG-965360), the Swedish Heart Lung Foundation (20220334), the Novonordisk Distinguished Investigator Grant - Endocrinology and Metabolism (NNF23OC0082114), the Novonordisk Project grants in Endocrinology and Metabolism (NNF24OC0091535) (SR).	
**Time frame: past 36 months**			
**2**	Grants or contracts from any entity (if not indicated in item #1 above).	X **None**	
**3**	Royalties or licenses	X **None**	
**4**	Consulting fees	X **None**	
**5**	Payment or honoraria for lectures, presentations, speakers bureaus, manuscript writing or educational events	X **None**	
**6**	Payment for expert testimony	x **None**	

		Name all entities with whom you have this relationship or indicate none (add rows as needed)	Specifications/Comments (e.g., if payments were made to you or to your institution)
7	Support for attending meetings and/or travel	x **None**	
8	Patents planned, issued or pending	x **None**	
9	Participation on a Data Safety Monitoring Board or Advisory Board	x **None**	
10	Leadership or fiduciary role in other board, society, committee or advocacy group, paid or unpaid	x **None**	
11	Stock or stock options	x **None**	
12	Receipt of equipment, materials, drugs, medical writing, gifts or other services	x **None**	
13	Other financial or non-financial interests	x **None**	

Please place an "X" next to the following statement to indicate your agreement:

x I certify that I have answered every question and have not altered the wording of any of the questions on this form.

ICMJE DISCLOSURE FORM

**Date:**	12/10/2025
**Your Name:**	Rosellina M Mancina
**Manuscript Title:**	***Carriage of rare APOB variants predisposes to severe steatotic liver disease and hepatocellular carcinoma***
**Manuscript Number (if known):**	201762-JCI-CRPH-1

In the interest of transparency, we ask you to disclose all relationships/activities/interests listed below that are related to the content of your manuscript. "Related" means any relation with for-profit or not-for-profit third parties whose interests may be affected by the content of the manuscript. Disclosure represents a commitment to transparency and does not necessarily indicate a bias. If you are in doubt about whether to list a relationship/activity/interest, it is preferable that you do so.

The author's relationships/activities/interests should be defined broadly. For example, if your manuscript pertains to the epidemiology of hypertension, you should declare all relationships with manufacturers of antihypertensive medication, even if that medication is not mentioned in the manuscript.

In item #1 below, report all support for the work reported in this manuscript without time limit. For all other items, the time frame for disclosure is the past 36 months.

		**Name all entities with whom you have this relationship or indicate none (add rows as needed)**	**Specifications/Comments (e.g., if payments were made to you or to your institution)**
		**Time frame: Since the initial planning of the work**	
**1**	All support for the present manuscript (e.g., funding, provision of study materials, medical writing, article processing charges, etc.) **No time limit for this item.**	☐ **None**	
		NIH since MV received funding via R01DK134575 from the National Institute for Diabetes and Digestive and Kidney Diseases (MV); Italian Ministry of Health (Ministero della Salute), Ricerca Finalizzata 2021 RF-2021-12373889, Italian Ministry of Health, Ricerca Finalizzata PNRR 2022 "RATIONAL" PNRR-MAD-2022-12375656 (LV); Italian Ministry of Health (Ministero della Salute), Fondazione IRCCS Ca' Granda Ospedale Maggiore Policlinico, Ricerca Corrente (DP); The European Union, H2020-ICT-2018-20/H2020-ICT-2020-2 programme "Photonics" under grant agreement No. 101016726 - REVEAL (LV). The European Union, HORIZON-MISS-2021-CANCER-02-03 programme "Genial" under grant agreement "101096312" (LV, SP); Italian Ministry of Research (MUR) PNRR – M4 - C2 "National Center for Gene Therapy and Drugs based on RNA Technology" CN3, Spoke 4 "ASSET" (LV); PRIN 2022 MUR:	Funding provided to institution.

		Name all entities with whom you have this relationship or indicate none (add rows as needed)	Specifications/Comments (e.g., if payments were made to you or to your institution)
		"Disentangling genetic, epigenetic and hormonal regulation of Fe/heme metabolism in the gender-specific nature of NAFLD (DEFENDER)"; Bando Ricerca Corrente and Piano Nazionale Complementare Ecosistema Innovativo della Salute - Hub Life Science-Diagnostica Avanzata (HLS-DA)' - PNC-E3-2022-23683266 - 'INNOVA' (LV); Department of Pathophysiology and Transplantation, University of Milan, is funded by the Italian Ministry of Education and Research (MUR): Dipartimenti di Eccellenza Program 2023 to 2027) (LV); VA Merit Funding BX003362: "Genetics of Cardiometabolic Diseases in the VA Population" from the VA Office of R&D, Veterans Health Administration, USA (KMC, PST); The Swedish Cancerfonden (22 2270 Pj), the Swedish Research Council (Vetenskapsradet (VR), 2023-02079), the Swedish state under the Agreement between the Swedish government and the county councils (the ALF agreement, ALFGBG-965360), the Swedish Heart Lung Foundation (20220334), the Novonordisk Distinguished Investigator Grant - Endocrinology and Metabolism (NNF23OC0082114), the Novonordisk Project grants in Endocrinology and Metabolism (NNF24OC0091535) (SR).	
**Time frame: past 36 months**			
**2**	Grants or contracts from any entity (if not indicated in item #1 above).	X **None**	
**3**	Royalties or licenses	X **None**	

		Name all entities with whom you have this relationship or indicate none (add rows as needed)	Specifications/Comments (e.g., if payments were made to you or to your institution)
4	Consulting fees	X **None**	
5	Payment or honoraria for lectures, presentations, speakers bureaus, manuscript writing or educational events	X **None**	
6	Payment for expert testimony	x **None**	
7	Support for attending meetings and/or travel	x **None**	
8	Patents planned, issued or pending	x **None**	
9	Participation on a Data Safety Monitoring Board or Advisory Board	x **None**	
10	Leadership or fiduciary role in other board, society, committee or advocacy group, paid or unpaid	x **None**	

		Name all entities with whom you have this relationship or indicate none (add rows as needed)	Specifications/Comments (e.g., if payments were made to you or to your institution)
**11**	Stock or stock options	x **None**	
**12**	Receipt of equipment, materials, drugs, medical writing, gifts or other services	x **None**	
**13**	Other financial or non-financial interests	x **None**	

Please place an "X" next to the following statement to indicate your agreement:

x I certify that I have answered every question and have not altered the wording of any of the questions on this form.

ICMJE DISCLOSURE FORM

**Date:**	12/10/2025
**Your Name:**	Robertino Dilena
**Manuscript Title:**	***Carriage of rare APOB variants predisposes to severe steatotic liver disease and hepatocellular carcinoma***
**Manuscript Number (if known):**	201762-JCI-CRPH-1

In the interest of transparency, we ask you to disclose all relationships/activities/interests listed below that are related to the content of your manuscript. "Related" means any relation with for-profit or not-for-profit third parties whose interests may be affected by the content of the manuscript. Disclosure represents a commitment to transparency and does not necessarily indicate a bias. If you are in doubt about whether to list a relationship/activity/interest, it is preferable that you do so.

The author's relationships/activities/interests should be defined broadly. For example, if your manuscript pertains to the epidemiology of hypertension, you should declare all relationships with manufacturers of antihypertensive medication, even if that medication is not mentioned in the manuscript.

In item #1 below, report all support for the work reported in this manuscript without time limit. For all other items, the time frame for disclosure is the past 36 months.

		Name all entities with whom you have this relationship or indicate none (add rows as needed)	Specifications/Comments (e.g., if payments were made to you or to your institution)
**Time frame: Since the initial planning of the work**			
**1**	All support for the present manuscript (e.g., funding, provision of study materials, medical writing, article processing charges, etc.) **No time limit for this item.**	☐ **None**	
		NIH since MV received funding via R01DK134575 from the National Institute for Diabetes and Digestive and Kidney Diseases (MV); Italian Ministry of Health (Ministero della Salute), Ricerca Finalizzata 2021 RF-2021-12373889, Italian Ministry of Health, Ricerca Finalizzata PNRR 2022 "RATIONAL" PNRR-MAD-2022-12375656 (LV); Italian Ministry of Health (Ministero della Salute), Fondazione IRCCS Ca' Granda Ospedale Maggiore Policlinico, Ricerca Corrente (DP); The European Union, H2020-ICT-2018-20/H2020-ICT-2020-2 programme "Photonics" under grant agreement No. 101016726 - REVEAL (LV). The European Union, HORIZON-MISS-2021-CANCER-02-03 programme "Genial" under grant agreement "101096312" (LV, SP); Italian Ministry of Research (MUR) PNRR – M4 - C2 "National Center for Gene Therapy and Drugs based on RNA Technology" CN3, Spoke 4 "ASSET" (LV); PRIN 2022 MUR: "Disentangling genetic, epigenetic and hormonal regulation of Fe/heme metabolism in the gender-specific nature of NAFLD (DEFENDER)"; Bando Ricerca Corrente and Piano Nazionale Complementare Ecosistema Innovativo della Salute - Hub Life Science-Diagnostica Avanzata (HLS-DA)' - PNC-E3-2022-23683266 - 'INNOVA' (LV); Department of Pathophysiology and Transplantation, University of Milan, is funded by the Italian Ministry of Education and Research (MUR): Dipartimenti di Eccellenza Program 2023 to 2027) (LV); VA Merit Funding BX003362: "Genetics of Cardiometabolic Diseases in the VA Population" from the VA Office of R&D, Veterans Health Administration, USA (KMC, PST); The Swedish Cancerfonden (22 2270 Pj), the Swedish Research Council (Vetenskapsradet (VR), 2023-02079), the Swedish state under the Agreement between the Swedish government and the county councils (the ALF agreement, ALFGBG-965360), the Swedish Heart Lung	Funding provided to institution.

		Name all entities with whom you have this relationship or indicate none (add rows as needed)	Specifications/Comments (e.g., if payments were made to you or to your institution)
		Foundation (20220334), the Novonordisk Distinguished Investigator Grant - Endocrinology and Metabolism (NNF23OC0082114), the Novonordisk Project grants in Endocrinology and Metabolism (NNF24OC0091535) (SR).	
**Time frame: past 36 months**			
**2**	Grants or contracts from any entity (if not indicated in item #1 above).	X **None**	
**3**	Royalties or licenses	X **None**	
**4**	Consulting fees	X **None**	
**5**	Payment or honoraria for lectures, presentations, speakers bureaus, manuscript writing or educational events	X **None**	
**6**	Payment for expert testimony	x **None**	

		Name all entities with whom you have this relationship or indicate none (add rows as needed)	Specifications/Comments (e.g., if payments were made to you or to your institution)
**7**	Support for attending meetings and/or travel	x **None**	
**8**	Patents planned, issued or pending	x **None**	
**9**	Participation on a Data Safety Monitoring Board or Advisory Board	x **None**	
**10**	Leadership or fiduciary role in other board, society, committee or advocacy group, paid or unpaid	x **None**	
**11**	Stock or stock options	x **None**	
**12**	Receipt of equipment, materials, drugs, medical writing, gifts or other services	x **None**	
**13**	Other financial or non-financial interests	x **None**	

Please place an "X" next to the following statement to indicate your agreement:

x I certify that I have answered every question and have not altered the wording of any of the questions on this form.

ICMJE DISCLOSURE FORM

**Date:**	12/10/2025
**Your Name:**	Roberta D'Ambrosio
**Manuscript Title:**	***Carriage of rare APOB variants predisposes to severe steatotic liver disease and hepatocellular carcinoma***
**Manuscript Number (if known):**	201762-JCI-CRPH-1

In the interest of transparency, we ask you to disclose all relationships/activities/interests listed below that are related to the content of your manuscript. "Related" means any relation with for-profit or not-for-profit third parties whose interests may be affected by the content of the manuscript. Disclosure represents a commitment to transparency and does not necessarily indicate a bias. If you are in doubt about whether to list a relationship/activity/interest, it is preferable that you do so.

The author's relationships/activities/interests should be defined broadly. For example, if your manuscript pertains to the epidemiology of hypertension, you should declare all relationships with manufacturers of antihypertensive medication, even if that medication is not mentioned in the manuscript.

In item #1 below, report all support for the work reported in this manuscript without time limit. For all other items, the time frame for disclosure is the past 36 months.

		Name all entities with whom you have this relationship or indicate none (add rows as needed)	Specifications/Comments (e.g., if payments were made to you or to your institution)
		**Time frame: Since the initial planning of the work**	
**1**	All support for the present manuscript (e.g., funding, provision of study materials, medical writing, article processing charges, etc.) **No time limit for this item.**	☐ **None**	
		NIH since MV received funding via R01DK134575 from the National Institute for Diabetes and Digestive and Kidney Diseases (MV); Italian Ministry of Health (Ministero della Salute), Ricerca Finalizzata 2021 RF-2021-12373889, Italian Ministry of Health, Ricerca Finalizzata PNRR 2022 "RATIONAL" PNRR-MAD-2022-12375656 (LV); Italian Ministry of Health (Ministero della Salute), Fondazione IRCCS Ca' Granda Ospedale Maggiore Policlinico, Ricerca Corrente (DP); The European Union, H2020-ICT-2018-20/H2020-ICT-2020-2 programme "Photonics" under grant agreement No. 101016726 - REVEAL (LV). The European Union, HORIZON-MISS-2021-CANCER-02-03 programme "Genial" under grant agreement "101096312" (LV, SP); Italian Ministry of Research (MUR) PNRR – M4 - C2 "National Center for Gene Therapy and Drugs based on RNA Technology" CN3, Spoke 4 "ASSET" (LV); PRIN 2022 MUR:	Funding provided to institution.

		Name all entities with whom you have this relationship or indicate none (add rows as needed)	Specifications/Comments (e.g., if payments were made to you or to your institution)
		"Disentangling genetic, epigenetic and hormonal regulation of Fe/heme metabolism in the gender-specific nature of NAFLD (DEFENDER)"; Bando Ricerca Corrente and Piano Nazionale Complementare Ecosistema Innovativo della Salute - Hub Life Science-Diagnostica Avanzata (HLS-DA)' - PNC-E3-2022-23683266 - 'INNOVA' (LV); Department of Pathophysiology and Transplantation, University of Milan, is funded by the Italian Ministry of Education and Research (MUR): Dipartimenti di Eccellenza Program 2023 to 2027) (LV); VA Merit Funding BX003362: "Genetics of Cardiometabolic Diseases in the VA Population" from the VA Office of R&D, Veterans Health Administration, USA (KMC, PST); The Swedish Cancerfonden (22 2270 Pj), the Swedish Research Council (Vetenskapsradet (VR), 2023-02079), the Swedish state under the Agreement between the Swedish government and the county councils (the ALF agreement, ALFGBG-965360), the Swedish Heart Lung Foundation (20220334), the Novonordisk Distinguished Investigator Grant - Endocrinology and Metabolism (NNF23OC0082114), the Novonordisk Project grants in Endocrinology and Metabolism (NNF24OC0091535) (SR).	

Time frame: past 36 months

**2**	Grants or contracts from any entity (if not indicated in item #1 above).	X **None**	
**3**	Royalties or licenses	X **None**	

		Name all entities with whom you have this relationship or indicate none (add rows as needed)	Specifications/Comments (e.g., if payments were made to you or to your institution)
4	Consulting fees	X None	
5	Payment or honoraria for lectures, presentations, speakers bureaus, manuscript writing or educational events	X None	
6	Payment for expert testimony	x None	
7	Support for attending meetings and/or travel	x None	
8	Patents planned, issued or pending	x None	
9	Participation on a Data Safety Monitoring Board or Advisory Board	x None	
10	Leadership or fiduciary role in other board, society, committee or advocacy group, paid or unpaid	x None	

		Name all entities with whom you have this relationship or indicate none (add rows as needed)	Specifications/Comments (e.g., if payments were made to you or to your institution)
11	Stock or stock options	x None	
12	Receipt of equipment, materials, drugs, medical writing, gifts or other services	x None	
13	Other financial or non-financial interests	x None	

Please place an "X" next to the following statement to indicate your agreement:

x I certify that I have answered every question and have not altered the wording of any of the questions on this form.

ICMJE DISCLOSURE FORM

Date: 12/10/2025

Your Name: Philip S Tsao

Manuscript Title: ***Carriage of rare APOB variants predisposes to severe steatotic liver disease and hepatocellular carcinoma***

Manuscript Number (if known): 201762-JCI-CRPH-1

In the interest of transparency, we ask you to disclose all relationships/activities/interests listed below that are related to the content of your manuscript. "Related" means any relation with for-profit or not-for-profit third parties whose interests may be affected by the content of the manuscript. Disclosure represents a commitment to transparency and does not necessarily indicate a bias. If you are in doubt about whether to list a relationship/activity/interest, it is preferable that you do so.

The author's relationships/activities/interests should be defined broadly. For example, if your manuscript pertains to the epidemiology of hypertension, you should declare all relationships with manufacturers of antihypertensive medication, even if that medication is not mentioned in the manuscript.

In item #1 below, report all support for the work reported in this manuscript without time limit. For all other items, the time frame for disclosure is the past 36 months.

		Name all entities with whom you have this relationship or indicate none (add rows as needed)	Specifications/Comments (e.g., if payments were made to you or to your institution)
**Time frame: Since the initial planning of the work**			
**1**	All support for the present manuscript (e.g., funding, provision of study materials, medical writing, article processing charges, etc.) **No time limit for this item.**	☐ **None**	
		NIH since MV received funding via R01DK134575 from the National Institute for Diabetes and Digestive and Kidney Diseases (MV); Italian Ministry of Health (Ministero della Salute), Ricerca Finalizzata 2021 RF-2021-12373889, Italian Ministry of Health, Ricerca Finalizzata PNRR 2022 "RATIONAL" PNRR-MAD-2022-12375656 (LV); Italian Ministry of Health (Ministero della Salute), Fondazione IRCCS Ca' Granda Ospedale Maggiore Policlinico, Ricerca Corrente (DP); The European Union, H2020-ICT-2018-20/H2020-ICT-2020-2 programme "Photonics" under grant agreement No. 101016726 - REVEAL (LV). The European Union, HORIZON-MISS-2021-CANCER-02-03 programme "Genial" under grant agreement "101096312" (LV, SP); Italian Ministry of Research (MUR) PNRR – M4 - C2 "National Center for Gene Therapy and Drugs based on RNA Technology" CN3, Spoke 4 "ASSET" (LV); PRIN 2022 MUR: "Disentangling genetic, epigenetic and hormonal regulation of Fe/heme metabolism in the gender-specific nature of NAFLD (DEFENDER)"; Bando Ricerca Corrente and Piano Nazionale Complementare Ecosistema Innovativo della Salute - Hub Life Science-Diagnostica Avanzata (HLS-DA)' - PNC-E3-2022-23683266 - 'INNOVA' (LV); Department of Pathophysiology and Transplantation, University of Milan, is funded by the Italian Ministry of Education and Research (MUR): Dipartimenti di Eccellenza Program 2023 to 2027) (LV); VA Merit Funding BX003362: "Genetics of Cardiometabolic Diseases in the VA Population" from the VA Office of R&D, Veterans Health Administration, USA (KMC, PST); The Swedish Cancerfonden (22 2270 Pj), the Swedish Research Council (Vetenskapsradet (VR), 2023-02079), the Swedish state under the Agreement between the Swedish government and the county councils (the ALF agreement, ALFGBG-965360), the Swedish Heart Lung	Funding provided to institution.

		Name all entities with whom you have this relationship or indicate none (add rows as needed)	Specifications/Comments (e.g., if payments were made to you or to your institution)
		Foundation (20220334), the Novonordisk Distinguished Investigator Grant - Endocrinology and Metabolism (NNF23OC0082114), the Novonordisk Project grants in Endocrinology and Metabolism (NNF24OC0091535) (SR).	
**Time frame: past 36 months**			
**2**	Grants or contracts from any entity (if not indicated in item #1 above).	X **None**	
**3**	Royalties or licenses	X **None**	
**4**	Consulting fees	X **None**	
**5**	Payment or honoraria for lectures, presentations, speakers bureaus, manuscript writing or educational events	X **None**	
**6**	Payment for expert testimony	x **None**	

		Name all entities with whom you have this relationship or indicate none (add rows as needed)	Specifications/Comments (e.g., if payments were made to you or to your institution)
7	Support for attending meetings and/or travel	x **None**	
8	Patents planned, issued or pending	x **None**	
9	Participation on a Data Safety Monitoring Board or Advisory Board	x **None**	
10	Leadership or fiduciary role in other board, society, committee or advocacy group, paid or unpaid	x **None**	
11	Stock or stock options	x **None**	
12	Receipt of equipment, materials, drugs, medical writing, gifts or other services	x **None**	
13	Other financial or non-financial interests	x **None**	

Please place an "X" next to the following statement to indicate your agreement:

x I certify that I have answered every question and have not altered the wording of any of the questions on this form.

ICMJE DISCLOSURE FORM

**Date:**	12/10/2025
**Your Name:**	Paolo Parini
**Manuscript Title:**	***Carriage of rare APOB variants predisposes to severe steatotic liver disease and hepatocellular carcinoma***
**Manuscript Number (if known):**	201762-JCI-CRPH-1

In the interest of transparency, we ask you to disclose all relationships/activities/interests listed below that are related to the content of your manuscript. "Related" means any relation with for-profit or not-for-profit third parties whose interests may be affected by the content of the manuscript. Disclosure represents a commitment to transparency and does not necessarily indicate a bias. If you are in doubt about whether to list a relationship/activity/interest, it is preferable that you do so.

The author's relationships/activities/interests should be defined broadly. For example, if your manuscript pertains to the epidemiology of hypertension, you should declare all relationships with manufacturers of antihypertensive medication, even if that medication is not mentioned in the manuscript.

In item #1 below, report all support for the work reported in this manuscript without time limit. For all other items, the time frame for disclosure is the past 36 months.

		Name all entities with whom you have this relationship or indicate none (add rows as needed)	Specifications/Comments (e.g., if payments were made to you or to your institution)
		**Time frame: Since the initial planning of the work**	
**1**	All support for the present manuscript (e.g., funding, provision of study materials, medical writing, article processing charges, etc.)<br>**No time limit for this item.**	☐ **None**	
		NIH since MV received funding via R01DK134575 from the National Institute for Diabetes and Digestive and Kidney Diseases (MV); Italian Ministry of Health (Ministero della Salute), Ricerca Finalizzata 2021 RF-2021-12373889, Italian Ministry of Health, Ricerca Finalizzata PNRR 2022 "RATIONAL" PNRR-MAD-2022-12375656 (LV); Italian Ministry of Health (Ministero della Salute), Fondazione IRCCS Ca' Granda Ospedale Maggiore Policlinico, Ricerca Corrente (DP); The European Union, H2020-ICT-2018-20/H2020-ICT-2020-2 programme "Photonics" under grant agreement No. 101016726 - REVEAL (LV). The European Union, HORIZON-MISS-2021-CANCER-02-03 programme "Genial" under grant agreement "101096312" (LV, SP); Italian Ministry of Research (MUR) PNRR – M4 - C2 "National Center for Gene Therapy and Drugs based on RNA Technology" CN3, Spoke 4 "ASSET" (LV); PRIN 2022 MUR:	Funding provided to institution.

		Name all entities with whom you have this relationship or indicate none (add rows as needed)	Specifications/Comments (e.g., if payments were made to you or to your institution)
		"Disentangling genetic, epigenetic and hormonal regulation of Fe/heme metabolism in the gender-specific nature of NAFLD (DEFENDER)"; Bando Ricerca Corrente and Piano Nazionale Complementare Ecosistema Innovativo della Salute - Hub Life Science-Diagnostica Avanzata (HLS-DA)' - PNC-E3-2022-23683266 - 'INNOVA' (LV); Department of Pathophysiology and Transplantation, University of Milan, is funded by the Italian Ministry of Education and Research (MUR): Dipartimenti di Eccellenza Program 2023 to 2027) (LV); VA Merit Funding BX003362: "Genetics of Cardiometabolic Diseases in the VA Population" from the VA Office of R&D, Veterans Health Administration, USA (KMC, PST); The Swedish Cancerfonden (22 2270 Pj), the Swedish Research Council (Vetenskapsradet (VR), 2023-02079), the Swedish state under the Agreement between the Swedish government and the county councils (the ALF agreement, ALFGBG-965360), the Swedish Heart Lung Foundation (20220334), the Novonordisk Distinguished Investigator Grant - Endocrinology and Metabolism (NNF23OC0082114), the Novonordisk Project grants in Endocrinology and Metabolism (NNF24OC0091535) (SR).	
**Time frame: past 36 months**			
**2**	Grants or contracts from any entity (if not indicated in item #1 above).	X **None**	
**3**	Royalties or licenses	X **None**	

		Name all entities with whom you have this relationship or indicate none (add rows as needed)	Specifications/Comments (e.g., if payments were made to you or to your institution)
4	Consulting fees	X **None**	
5	Payment or honoraria for lectures, presentations, speakers bureaus, manuscript writing or educational events	X **None**	
6	Payment for expert testimony	x **None**	
7	Support for attending meetings and/or travel	x **None**	
8	Patents planned, issued or pending	x **None**	
9	Participation on a Data Safety Monitoring Board or Advisory Board	x **None**	
10	Leadership or fiduciary role in other board, society, committee or advocacy group, paid or unpaid	x **None**	

		Name all entities with whom you have this relationship or indicate none (add rows as needed)	Specifications/Comments (e.g., if payments were made to you or to your institution)
11	Stock or stock options	x **None**	
12	Receipt of equipment, materials, drugs, medical writing, gifts or other services	x **None**	
13	Other financial or non-financial interests	x **None**	

Please place an "X" next to the following statement to indicate your agreement:

x I certify that I have answered every question and have not altered the wording of any of the questions on this form.

ICMJE DISCLOSURE FORM

Date: 12/10/2025

Your Name: Oveis Jamialahmadi

Manuscript Title: ***Carriage of rare APOB variants predisposes to severe steatotic liver disease and hepatocellular carcinoma***

Manuscript Number (if known): 201762-JCI-CRPH-1

In the interest of transparency, we ask you to disclose all relationships/activities/interests listed below that are related to the content of your manuscript. "Related" means any relation with for-profit or not-for-profit third parties whose interests may be affected by the content of the manuscript. Disclosure represents a commitment to transparency and does not necessarily indicate a bias. If you are in doubt about whether to list a relationship/activity/interest, it is preferable that you do so.

The author's relationships/activities/interests should be defined broadly. For example, if your manuscript pertains to the epidemiology of hypertension, you should declare all relationships with manufacturers of antihypertensive medication, even if that medication is not mentioned in the manuscript.

In item #1 below, report all support for the work reported in this manuscript without time limit. For all other items, the time frame for disclosure is the past 36 months.

		Name all entities with whom you have this relationship or indicate none (add rows as needed)	Specifications/Comments (e.g., if payments were made to you or to your institution)
**Time frame: Since the initial planning of the work**			
**1**	All support for the present manuscript (e.g., funding, provision of study materials, medical writing, article processing charges, etc.)<br>**No time limit for this item.**	☐ **None**	
		NIH since MV received funding via R01DK134575 from the National Institute for Diabetes and Digestive and Kidney Diseases (MV); Italian Ministry of Health (Ministero della Salute), Ricerca Finalizzata 2021 RF-2021-12373889, Italian Ministry of Health, Ricerca Finalizzata PNRR 2022 "RATIONAL" PNRR-MAD-2022-12375656 (LV); Italian Ministry of Health (Ministero della Salute), Fondazione IRCCS Ca' Granda Ospedale Maggiore Policlinico, Ricerca Corrente (DP); The European Union, H2020-ICT-2018-20/H2020-ICT-2020-2 programme "Photonics" under grant agreement No. 101016726 - REVEAL (LV). The European Union, HORIZON-MISS-2021-CANCER-02-03 programme "Genial" under grant agreement "101096312" (LV, SP); Italian Ministry of Research (MUR) PNRR – M4 - C2 "National Center for Gene Therapy and Drugs based on RNA Technology" CN3, Spoke 4 "ASSET" (LV); PRIN 2022 MUR: "Disentangling genetic, epigenetic and hormonal regulation of Fe/heme metabolism in the gender-specific nature of NAFLD (DEFENDER)"; Bando Ricerca Corrente and Piano Nazionale Complementare Ecosistema Innovativo della Salute - Hub Life Science-Diagnostica Avanzata (HLS-DA)' - PNC-E3-2022-23683266 - 'INNOVA' (LV); Department of Pathophysiology and Transplantation, University of Milan, is funded by the Italian Ministry of Education and Research (MUR): Dipartimenti di Eccellenza Program 2023 to 2027) (LV); VA Merit Funding BX003362: "Genetics of Cardiometabolic Diseases in the VA Population" from the VA Office of R&D, Veterans Health Administration, USA (KMC, PST); The Swedish Cancerfonden (22 2270 Pj), the Swedish Research Council (Vetenskapsradet (VR), 2023-02079), the Swedish state under the Agreement between the Swedish government and the county councils (the ALF agreement, ALFGBG-965360), the Swedish Heart Lung	Funding provided to institution.

		Name all entities with whom you have this relationship or indicate none (add rows as needed)	Specifications/Comments (e.g., if payments were made to you or to your institution)
		Foundation (20220334), the Novonordisk Distinguished Investigator Grant - Endocrinology and Metabolism (NNF23OC0082114), the Novonordisk Project grants in Endocrinology and Metabolism (NNF24OC0091535) (SR).	
**Time frame: past 36 months**			
**2**	Grants or contracts from any entity (if not indicated in item #1 above).	X **None**	
**3**	Royalties or licenses	X **None**	
**4**	Consulting fees	X **None**	
**5**	Payment or honoraria for lectures, presentations, speakers bureaus, manuscript writing or educational events	X **None**	
**6**	Payment for expert testimony	x **None**	

		Name all entities with whom you have this relationship or indicate none (add rows as needed)	Specifications/Comments (e.g., if payments were made to you or to your institution)
7	Support for attending meetings and/or travel	x **None**	
8	Patents planned, issued or pending	x **None**	
9	Participation on a Data Safety Monitoring Board or Advisory Board	x **None**	
10	Leadership or fiduciary role in other board, society, committee or advocacy group, paid or unpaid	x **None**	
11	Stock or stock options	x **None**	
12	Receipt of equipment, materials, drugs, medical writing, gifts or other services	x **None**	
13	Other financial or non-financial interests	x **None**	

Please place an "X" next to the following statement to indicate your agreement:

x I certify that I have answered every question and have not altered the wording of any of the questions on this form.

ICMJE DISCLOSURE FORM

**Date:**	12/10/2025
**Your Name:**	Mirella Fraquelli
**Manuscript Title:**	***Carriage of rare APOB variants predisposes to severe steatotic liver disease and hepatocellular carcinoma***
**Manuscript Number (if known):**	201762-JCI-CRPH-1

In the interest of transparency, we ask you to disclose all relationships/activities/interests listed below that are related to the content of your manuscript. "Related" means any relation with for-profit or not-for-profit third parties whose interests may be affected by the content of the manuscript. Disclosure represents a commitment to transparency and does not necessarily indicate a bias. If you are in doubt about whether to list a relationship/activity/interest, it is preferable that you do so.

The author's relationships/activities/interests should be defined broadly. For example, if your manuscript pertains to the epidemiology of hypertension, you should declare all relationships with manufacturers of antihypertensive medication, even if that medication is not mentioned in the manuscript.

In item #1 below, report all support for the work reported in this manuscript without time limit. For all other items, the time frame for disclosure is the past 36 months.

		Name all entities with whom you have this relationship or indicate none (add rows as needed)	Specifications/Comments (e.g., if payments were made to you or to your institution)
**Time frame: Since the initial planning of the work**			
**1**	All support for the present manuscript (e.g., funding, provision of study materials, medical writing, article processing charges, etc.)<br>**No time limit for this item.**	☐ **None**	
		NIH since MV received funding via R01DK134575 from the National Institute for Diabetes and Digestive and Kidney Diseases (MV); Italian Ministry of Health (Ministero della Salute), Ricerca Finalizzata 2021 RF-2021-12373889, Italian Ministry of Health, Ricerca Finalizzata PNRR 2022 "RATIONAL" PNRR-MAD-2022-12375656 (LV); Italian Ministry of Health (Ministero della Salute), Fondazione IRCCS Ca' Granda Ospedale Maggiore Policlinico, Ricerca Corrente (DP); The European Union, H2020-ICT-2018-20/H2020-ICT-2020-2 programme "Photonics" under grant agreement No. 101016726 - REVEAL (LV). The European Union, HORIZON-MISS-2021-CANCER-02-03 programme "Genial" under grant agreement "101096312" (LV, SP); Italian Ministry of Research (MUR) PNRR – M4 - C2 "National Center for Gene Therapy and Drugs based on RNA Technology" CN3, Spoke 4 "ASSET" (LV); PRIN 2022 MUR:	Funding provided to institution.

		Name all entities with whom you have this relationship or indicate none (add rows as needed)	Specifications/Comments (e.g., if payments were made to you or to your institution)
		"Disentangling genetic, epigenetic and hormonal regulation of Fe/heme metabolism in the gender-specific nature of NAFLD (DEFENDER)"; Bando Ricerca Corrente and Piano Nazionale Complementare Ecosistema Innovativo della Salute - Hub Life Science-Diagnostica Avanzata (HLS-DA)' - PNC-E3-2022-23683266 - 'INNOVA' (LV); Department of Pathophysiology and Transplantation, University of Milan, is funded by the Italian Ministry of Education and Research (MUR): Dipartimenti di Eccellenza Program 2023 to 2027) (LV); VA Merit Funding BX003362: "Genetics of Cardiometabolic Diseases in the VA Population" from the VA Office of R&D, Veterans Health Administration, USA (KMC, PST); The Swedish Cancerfonden (22 2270 Pj), the Swedish Research Council (Vetenskapsradet (VR), 2023-02079), the Swedish state under the Agreement between the Swedish government and the county councils (the ALF agreement, ALFGBG-965360), the Swedish Heart Lung Foundation (20220334), the Novonordisk Distinguished Investigator Grant - Endocrinology and Metabolism (NNF23OC0082114), the Novonordisk Project grants in Endocrinology and Metabolism (NNF24OC0091535) (SR).	

Time frame: past 36 months

**2**	Grants or contracts from any entity (if not indicated in item #1 above).	X **None**	
**3**	Royalties or licenses	X **None**	

		Name all entities with whom you have this relationship or indicate none (add rows as needed)	Specifications/Comments (e.g., if payments were made to you or to your institution)
**4**	Consulting fees	X **None**	
**5**	Payment or honoraria for lectures, presentations, speakers bureaus, manuscript writing or educational events	X **None**	
**6**	Payment for expert testimony	x **None**	
**7**	Support for attending meetings and/or travel	x **None**	
**8**	Patents planned, issued or pending	x **None**	
**9**	Participation on a Data Safety Monitoring Board or Advisory Board	x **None**	
**10**	Leadership or fiduciary role in other board, society, committee or advocacy group, paid or unpaid	x **None**	

		Name all entities with whom you have this relationship or indicate none (add rows as needed)	Specifications/Comments (e.g., if payments were made to you or to your institution)
11	Stock or stock options	x **None**	
12	Receipt of equipment, materials, drugs, medical writing, gifts or other services	x **None**	
13	Other financial or non-financial interests	x **None**	

Please place an "X" next to the following statement to indicate your agreement:

x I certify that I have answered every question and have not altered the wording of any of the questions on this form.

ICMJE DISCLOSURE FORM

**Date:**	12/10/2025
**Your Name:**	Josephine P Johnson
**Manuscript Title:**	***Carriage of rare APOB variants predisposes to severe steatotic liver disease and hepatocellular carcinoma***
**Manuscript Number (if known):**	201762-JCI-CRPH-1

In the interest of transparency, we ask you to disclose all relationships/activities/interests listed below that are related to the content of your manuscript. "Related" means any relation with for-profit or not-for-profit third parties whose interests may be affected by the content of the manuscript. Disclosure represents a commitment to transparency and does not necessarily indicate a bias. If you are in doubt about whether to list a relationship/activity/interest, it is preferable that you do so.

The author's relationships/activities/interests should be defined broadly. For example, if your manuscript pertains to the epidemiology of hypertension, you should declare all relationships with manufacturers of antihypertensive medication, even if that medication is not mentioned in the manuscript.

In item #1 below, report all support for the work reported in this manuscript without time limit. For all other items, the time frame for disclosure is the past 36 months.

		Name all entities with whom you have this relationship or indicate none (add rows as needed)	Specifications/Comments (e.g., if payments were made to you or to your institution)
**Time frame: Since the initial planning of the work**			
**1**	All support for the present manuscript (e.g., funding, provision of study materials, medical writing, article processing charges, etc.)<br>**No time limit for this item.**	☐ **None**	
		NIH since MV received funding via R01DK134575 from the National Institute for Diabetes and Digestive and Kidney Diseases (MV); Italian Ministry of Health (Ministero della Salute), Ricerca Finalizzata 2021 RF-2021-12373889, Italian Ministry of Health, Ricerca Finalizzata PNRR 2022 "RATIONAL" PNRR-MAD-2022-12375656 (LV); Italian Ministry of Health (Ministero della Salute), Fondazione IRCCS Ca' Granda Ospedale Maggiore Policlinico, Ricerca Corrente (DP); The European Union, H2020-ICT-2018-20/H2020-ICT-2020-2 programme "Photonics" under grant agreement No. 101016726 - REVEAL (LV). The European Union, HORIZON-MISS-2021-CANCER-02-03 programme "Genial" under grant agreement "101096312" (LV, SP); Italian Ministry of Research (MUR) PNRR – M4 - C2 "National Center for Gene Therapy and Drugs based on RNA Technology" CN3, Spoke 4 "ASSET" (LV); PRIN 2022 MUR: "Disentangling genetic, epigenetic and hormonal regulation of Fe/heme metabolism in the gender-specific nature of NAFLD (DEFENDER)"; Bando Ricerca Corrente and Piano Nazionale Complementare Ecosistema Innovativo della Salute - Hub Life Science-Diagnostica Avanzata (HLS-DA)' - PNC-E3-2022-23683266 - 'INNOVA' (LV); Department of Pathophysiology and Transplantation, University of Milan, is funded by the Italian Ministry of Education and Research (MUR): Dipartimenti di Eccellenza Program 2023 to 2027) (LV); VA Merit Funding BX003362: "Genetics of Cardiometabolic Diseases in the VA Population" from the VA Office of R&D, Veterans Health Administration, USA (KMC, PST); The Swedish Cancerfonden (22 2270 Pj), the Swedish Research Council (Vetenskapsradet (VR), 2023-02079), the Swedish state under the Agreement between the Swedish government and the county councils (the ALF agreement, ALFGBG-965360), the Swedish Heart Lung	Funding provided to institution.

		Name all entities with whom you have this relationship or indicate none (add rows as needed)	Specifications/Comments (e.g., if payments were made to you or to your institution)
		Foundation (20220334), the Novonordisk Distinguished Investigator Grant - Endocrinology and Metabolism (NNF23OC0082114), the Novonordisk Project grants in Endocrinology and Metabolism (NNF24OC0091535) (SR).	
**Time frame: past 36 months**			
**2**	Grants or contracts from any entity (if not indicated in item #1 above).	X **None**	
**3**	Royalties or licenses	X **None**	
**4**	Consulting fees	X **None**	
**5**	Payment or honoraria for lectures, presentations, speakers bureaus, manuscript writing or educational events	X **None**	
**6**	Payment for expert testimony	x **None**	

		Name all entities with whom you have this relationship or indicate none (add rows as needed)	Specifications/Comments (e.g., if payments were made to you or to your institution)
7	Support for attending meetings and/or travel	x **None**	
8	Patents planned, issued or pending	x **None**	
9	Participation on a Data Safety Monitoring Board or Advisory Board	x **None**	
10	Leadership or fiduciary role in other board, society, committee or advocacy group, paid or unpaid	x **None**	
11	Stock or stock options	x **None**	
12	Receipt of equipment, materials, drugs, medical writing, gifts or other services	x **None**	
13	Other financial or non-financial interests	x **None**	

Please place an "X" next to the following statement to indicate your agreement:

x I certify that I have answered every question and have not altered the wording of any of the questions on this form.

ICMJE DISCLOSURE FORM

**Date:**	12/10/2025
**Your Name:**	Marijana Vujkovic
**Manuscript Title:**	***Carriage of rare APOB variants predisposes to severe steatotic liver disease and hepatocellular carcinoma***
**Manuscript Number (if known):**	201762-JCI-CRPH-1

In the interest of transparency, we ask you to disclose all relationships/activities/interests listed below that are related to the content of your manuscript. "Related" means any relation with for-profit or not-for-profit third parties whose interests may be affected by the content of the manuscript. Disclosure represents a commitment to transparency and does not necessarily indicate a bias. If you are in doubt about whether to list a relationship/activity/interest, it is preferable that you do so.

The author's relationships/activities/interests should be defined broadly. For example, if your manuscript pertains to the epidemiology of hypertension, you should declare all relationships with manufacturers of antihypertensive medication, even if that medication is not mentioned in the manuscript.

In item #1 below, report all support for the work reported in this manuscript without time limit. For all other items, the time frame for disclosure is the past 36 months.

		**Name all entities with whom you have this relationship or indicate none (add rows as needed)**	**Specifications/Comments (e.g., if payments were made to you or to your institution)**
		**Time frame: Since the initial planning of the work**	
**1**	All support for the present manuscript (e.g., funding, provision of study materials, medical writing, article processing charges, etc.) **No time limit for this item.**	☐ **None**	
		NIH since MV received funding via R01DK134575 from the National Institute for Diabetes and Digestive and Kidney Diseases (MV); Italian Ministry of Health (Ministero della Salute), Ricerca Finalizzata 2021 RF-2021-12373889, Italian Ministry of Health, Ricerca Finalizzata PNRR 2022 "RATIONAL" PNRR-MAD-2022-12375656 (LV); Italian Ministry of Health (Ministero della Salute), Fondazione IRCCS Ca' Granda Ospedale Maggiore Policlinico, Ricerca Corrente (DP); The European Union, H2020-ICT-2018-20/H2020-ICT-2020-2 programme "Photonics" under grant agreement No. 101016726 - REVEAL (LV). The European Union, HORIZON-MISS-2021-CANCER-02-03 programme "Genial" under grant agreement "101096312" (LV, SP); Italian Ministry of Research (MUR) PNRR – M4 - C2 "National Center for Gene Therapy and Drugs based on RNA Technology" CN3, Spoke 4 "ASSET" (LV); PRIN 2022 MUR:	Funding provided to institution.

		Name all entities with whom you have this relationship or indicate none (add rows as needed)	Specifications/Comments (e.g., if payments were made to you or to your institution)
		"Disentangling genetic, epigenetic and hormonal regulation of Fe/heme metabolism in the gender-specific nature of NAFLD (DEFENDER)"; Bando Ricerca Corrente and Piano Nazionale Complementare Ecosistema Innovativo della Salute - Hub Life Science-Diagnostica Avanzata (HLS-DA)' - PNC-E3-2022-23683266 - 'INNOVA' (LV); Department of Pathophysiology and Transplantation, University of Milan, is funded by the Italian Ministry of Education and Research (MUR): Dipartimenti di Eccellenza Program 2023 to 2027) (LV); VA Merit Funding BX003362: "Genetics of Cardiometabolic Diseases in the VA Population" from the VA Office of R&D, Veterans Health Administration, USA (KMC, PST); The Swedish Cancerfonden (22 2270 Pj), the Swedish Research Council (Vetenskapsradet (VR), 2023-02079), the Swedish state under the Agreement between the Swedish government and the county councils (the ALF agreement, ALFGBG-965360), the Swedish Heart Lung Foundation (20220334), the Novonordisk Distinguished Investigator Grant - Endocrinology and Metabolism (NNF23OC0082114), the Novonordisk Project grants in Endocrinology and Metabolism (NNF24OC0091535) (SR).	
**Time frame: past 36 months**			
**2**	Grants or contracts from any entity (if not indicated in item #1 above).	X **None**	
**3**	Royalties or licenses	X **None**	

		Name all entities with whom you have this relationship or indicate none (add rows as needed)	Specifications/Comments (e.g., if payments were made to you or to your institution)
4	Consulting fees	X None	
5	Payment or honoraria for lectures, presentations, speakers bureaus, manuscript writing or educational events	X None	
6	Payment for expert testimony	x None	
7	Support for attending meetings and/or travel	x None	
8	Patents planned, issued or pending	x None	
9	Participation on a Data Safety Monitoring Board or Advisory Board	x None	
10	Leadership or fiduciary role in other board, society, committee or advocacy group, paid or unpaid	x None	

		Name all entities with whom you have this relationship or indicate none (add rows as needed)	Specifications/Comments (e.g., if payments were made to you or to your institution)
11	Stock or stock options	x None	
12	Receipt of equipment, materials, drugs, medical writing, gifts or other services	x None	
13	Other financial or non-financial interests	x None	

Please place an "X" next to the following statement to indicate your agreement:

x I certify that I have answered every question and have not altered the wording of any of the questions on this form.

ICMJE DISCLOSURE FORM

**Date:**	12/10/2025
**Your Name:**	Hadi Eidgah Torghabehei
**Manuscript Title:**	***Carriage of rare APOB variants predisposes to severe steatotic liver disease and hepatocellular carcinoma***
**Manuscript Number (if known):**	201762-JCI-CRPH-1

In the interest of transparency, we ask you to disclose all relationships/activities/interests listed below that are related to the content of your manuscript. "Related" means any relation with for-profit or not-for-profit third parties whose interests may be affected by the content of the manuscript. Disclosure represents a commitment to transparency and does not necessarily indicate a bias. If you are in doubt about whether to list a relationship/activity/interest, it is preferable that you do so.

The author's relationships/activities/interests should be defined broadly. For example, if your manuscript pertains to the epidemiology of hypertension, you should declare all relationships with manufacturers of antihypertensive medication, even if that medication is not mentioned in the manuscript.

In item #1 below, report all support for the work reported in this manuscript without time limit. For all other items, the time frame for disclosure is the past 36 months.

		Name all entities with whom you have this relationship or indicate none (add rows as needed)	Specifications/Comments (e.g., if payments were made to you or to your institution)
**Time frame: Since the initial planning of the work**			
**1**	All support for the present manuscript (e.g., funding, provision of study materials, medical writing, article processing charges, etc.) **No time limit for this item.**	☐ **None**	
		NIH since MV received funding via R01DK134575 from the National Institute for Diabetes and Digestive and Kidney Diseases (MV); Italian Ministry of Health (Ministero della Salute), Ricerca Finalizzata 2021 RF-2021-12373889, Italian Ministry of Health, Ricerca Finalizzata PNRR 2022 "RATIONAL" PNRR-MAD-2022-12375656 (LV); Italian Ministry of Health (Ministero della Salute), Fondazione IRCCS Ca' Granda Ospedale Maggiore Policlinico, Ricerca Corrente (DP); The European Union, H2020-ICT-2018-20/H2020-ICT-2020-2 programme "Photonics" under grant agreement No. 101016726 - REVEAL (LV). The European Union, HORIZON-MISS-2021-CANCER-02-03 programme "Genial" under grant agreement "101096312" (LV, SP); Italian Ministry of Research (MUR) PNRR – M4 - C2 "National Center for Gene Therapy and Drugs based on RNA Technology" CN3, Spoke 4 "ASSET" (LV); PRIN 2022 MUR: "Disentangling genetic, epigenetic and hormonal regulation of Fe/heme metabolism in the gender-specific nature of NAFLD (DEFENDER)"; Bando Ricerca Corrente and Piano Nazionale Complementare Ecosistema Innovativo della Salute - Hub Life Science-Diagnostica Avanzata (HLS-DA)' - PNC-E3-2022-23683266 - 'INNOVA' (LV); Department of Pathophysiology and Transplantation, University of Milan, is funded by the Italian Ministry of Education and Research (MUR): Dipartimenti di Eccellenza Program 2023 to 2027) (LV); VA Merit Funding BX003362: "Genetics of Cardiometabolic Diseases in the VA Population" from the VA Office of R&D, Veterans Health Administration, USA (KMC, PST); The Swedish Cancerfonden (22 2270 Pj), the Swedish Research Council (Vetenskapsradet (VR), 2023-02079), the Swedish state under the Agreement between the Swedish government and the county councils (the ALF agreement, ALFGBG-965360), the Swedish Heart Lung	Funding provided to institution.

		Name all entities with whom you have this relationship or indicate none (add rows as needed)	Specifications/Comments (e.g., if payments were made to you or to your institution)
		Foundation (20220334), the Novonordisk Distinguished Investigator Grant - Endocrinology and Metabolism (NNF23OC0082114), the Novonordisk Project grants in Endocrinology and Metabolism (NNF24OC0091535) (SR).	
**Time frame: past 36 months**			
**2**	Grants or contracts from any entity (if not indicated in item #1 above).	X **None**	
**3**	Royalties or licenses	X **None**	
**4**	Consulting fees	X **None**	
**5**	Payment or honoraria for lectures, presentations, speakers bureaus, manuscript writing or educational events	X **None**	
**6**	Payment for expert testimony	x **None**	

		Name all entities with whom you have this relationship or indicate none (add rows as needed)	Specifications/Comments (e.g., if payments were made to you or to your institution)
**7**	Support for attending meetings and/or travel	x **None**	
**8**	Patents planned, issued or pending	x **None**	
**9**	Participation on a Data Safety Monitoring Board or Advisory Board	x **None**	
**10**	Leadership or fiduciary role in other board, society, committee or advocacy group, paid or unpaid	x **None**	
**11**	Stock or stock options	x **None**	
**12**	Receipt of equipment, materials, drugs, medical writing, gifts or other services	x **None**	
**13**	Other financial or non-financial interests	x **None**	

Please place an "X" next to the following statement to indicate your agreement:

x I certify that I have answered every question and have not altered the wording of any of the questions on this form.

ICMJE DISCLOSURE FORM

**Date:**	12/10/2025
**Your Name:**	Giulia Periti
**Manuscript Title:**	***Carriage of rare APOB variants predisposes to severe steatotic liver disease and hepatocellular carcinoma***
**Manuscript Number (if known):**	201762-JCI-CRPH-1

In the interest of transparency, we ask you to disclose all relationships/activities/interests listed below that are related to the content of your manuscript. "Related" means any relation with for-profit or not-for-profit third parties whose interests may be affected by the content of the manuscript. Disclosure represents a commitment to transparency and does not necessarily indicate a bias. If you are in doubt about whether to list a relationship/activity/interest, it is preferable that you do so.

The author's relationships/activities/interests should be defined broadly. For example, if your manuscript pertains to the epidemiology of hypertension, you should declare all relationships with manufacturers of antihypertensive medication, even if that medication is not mentioned in the manuscript.

In item #1 below, report all support for the work reported in this manuscript without time limit. For all other items, the time frame for disclosure is the past 36 months.

		Name all entities with whom you have this relationship or indicate none (add rows as needed)	Specifications/Comments (e.g., if payments were made to you or to your institution)
**Time frame: Since the initial planning of the work**			
**1**	All support for the present manuscript (e.g., funding, provision of study materials, medical writing, article processing charges, etc.) **No time limit for this item.**	☐ **None**	
		NIH since MV received funding via R01DK134575 from the National Institute for Diabetes and Digestive and Kidney Diseases (MV); Italian Ministry of Health (Ministero della Salute), Ricerca Finalizzata 2021 RF-2021-12373889, Italian Ministry of Health, Ricerca Finalizzata PNRR 2022 "RATIONAL" PNRR-MAD-2022-12375656 (LV); Italian Ministry of Health (Ministero della Salute), Fondazione IRCCS Ca' Granda Ospedale Maggiore Policlinico, Ricerca Corrente (DP); The European Union, H2020-ICT-2018-20/H2020-ICT-2020-2 programme "Photonics" under grant agreement No. 101016726 - REVEAL (LV). The European Union, HORIZON-MISS-2021-CANCER-02-03 programme "Genial" under grant agreement "101096312" (LV, SP); Italian Ministry of Research (MUR) PNRR – M4 - C2 "National Center for Gene Therapy and Drugs based on RNA Technology" CN3, Spoke 4 "ASSET" (LV); PRIN 2022 MUR:	Funding provided to institution.

		Name all entities with whom you have this relationship or indicate none (add rows as needed)	Specifications/Comments (e.g., if payments were made to you or to your institution)
		"Disentangling genetic, epigenetic and hormonal regulation of Fe/heme metabolism in the gender-specific nature of NAFLD (DEFENDER)"; Bando Ricerca Corrente and Piano Nazionale Complementare Ecosistema Innovativo della Salute - Hub Life Science-Diagnostica Avanzata (HLS-DA)' - PNC-E3-2022-23683266 - 'INNOVA' (LV); Department of Pathophysiology and Transplantation, University of Milan, is funded by the Italian Ministry of Education and Research (MUR): Dipartimenti di Eccellenza Program 2023 to 2027) (LV); VA Merit Funding BX003362: "Genetics of Cardiometabolic Diseases in the VA Population" from the VA Office of R&D, Veterans Health Administration, USA (KMC, PST); The Swedish Cancerfonden (22 2270 Pj), the Swedish Research Council (Vetenskapsradet (VR), 2023-02079), the Swedish state under the Agreement between the Swedish government and the county councils (the ALF agreement, ALFGBG-965360), the Swedish Heart Lung Foundation (20220334), the Novonordisk Distinguished Investigator Grant - Endocrinology and Metabolism (NNF23OC0082114), the Novonordisk Project grants in Endocrinology and Metabolism (NNF24OC0091535) (SR).	

Time frame: past 36 months

**2**	Grants or contracts from any entity (if not indicated in item #1 above).	X **None**	
**3**	Royalties or licenses	X **None**	

		Name all entities with whom you have this relationship or indicate none (add rows as needed)	Specifications/Comments (e.g., if payments were made to you or to your institution)
4	Consulting fees	X **None**	
5	Payment or honoraria for lectures, presentations, speakers bureaus, manuscript writing or educational events	X **None**	
6	Payment for expert testimony	x **None**	
7	Support for attending meetings and/or travel	x **None**	
8	Patents planned, issued or pending	x **None**	
9	Participation on a Data Safety Monitoring Board or Advisory Board	x **None**	
10	Leadership or fiduciary role in other board, society, committee or advocacy group, paid or unpaid	x **None**	

		Name all entities with whom you have this relationship or indicate none (add rows as needed)	Specifications/Comments (e.g., if payments were made to you or to your institution)
11	Stock or stock options	x **None**	
12	Receipt of equipment, materials, drugs, medical writing, gifts or other services	x **None**	
13	Other financial or non-financial interests	x **None**	

Please place an "X" next to the following statement to indicate your agreement:

x I certify that I have answered every question and have not altered the wording of any of the questions on this form.

ICMJE DISCLOSURE FORM

Date: 12/10/2025

Your Name: Marco Saracino

Manuscript Title: ***Carriage of rare APOB variants predisposes to severe steatotic liver disease and hepatocellular carcinoma***

Manuscript Number (if known): 201762-JCI-CRPH-1

In the interest of transparency, we ask you to disclose all relationships/activities/interests listed below that are related to the content of your manuscript. "Related" means any relation with for-profit or not-for-profit third parties whose interests may be affected by the content of the manuscript. Disclosure represents a commitment to transparency and does not necessarily indicate a bias. If you are in doubt about whether to list a relationship/activity/interest, it is preferable that you do so.

The author's relationships/activities/interests should be defined broadly. For example, if your manuscript pertains to the epidemiology of hypertension, you should declare all relationships with manufacturers of antihypertensive medication, even if that medication is not mentioned in the manuscript.

In item #1 below, report all support for the work reported in this manuscript without time limit. For all other items, the time frame for disclosure is the past 36 months.

		Name all entities with whom you have this relationship or indicate none (add rows as needed)	Specifications/Comments (e.g., if payments were made to you or to your institution)
**Time frame: Since the initial planning of the work**			
**1**	All support for the present manuscript (e.g., funding, provision of study materials, medical writing, article processing charges, etc.)<br>**No time limit for this item.**	☐ **None**	
		NIH since MV received funding via R01DK134575 from the National Institute for Diabetes and Digestive and Kidney Diseases (MV); Italian Ministry of Health (Ministero della Salute), Ricerca Finalizzata 2021 RF-2021-12373889, Italian Ministry of Health, Ricerca Finalizzata PNRR 2022 "RATIONAL" PNRR-MAD-2022-12375656 (LV); Italian Ministry of Health (Ministero della Salute), Fondazione IRCCS Ca' Granda Ospedale Maggiore Policlinico, Ricerca Corrente (DP); The European Union, H2020-ICT-2018-20/H2020-ICT-2020-2 programme "Photonics" under grant agreement No. 101016726 - REVEAL (LV). The European Union, HORIZON-MISS-2021-CANCER-02-03 programme "Genial" under grant agreement "101096312" (LV, SP); Italian Ministry of Research (MUR) PNRR – M4 - C2 "National Center for Gene Therapy and Drugs based on RNA Technology" CN3, Spoke 4 "ASSET" (LV); PRIN 2022 MUR: "Disentangling genetic, epigenetic and hormonal regulation of Fe/heme metabolism in the gender-specific nature of NAFLD (DEFENDER)"; Bando Ricerca Corrente and Piano Nazionale Complementare Ecosistema Innovativo della Salute - Hub Life Science-Diagnostica Avanzata (HLS-DA)' - PNC-E3-2022-23683266 - 'INNOVA' (LV); Department of Pathophysiology and Transplantation, University of Milan, is funded by the Italian Ministry of Education and Research (MUR): Dipartimenti di Eccellenza Program 2023 to 2027) (LV); VA Merit Funding BX003362: "Genetics of Cardiometabolic Diseases in the VA Population" from the VA Office of R&D, Veterans Health Administration, USA (KMC, PST); The Swedish Cancerfonden (22 2270 Pj), the Swedish Research Council (Vetenskapsradet (VR), 2023-02079), the Swedish state under the Agreement between the Swedish government and the county councils (the ALF agreement, ALFGBG-965360), the Swedish Heart Lung	Funding provided to institution.

		Name all entities with whom you have this relationship or indicate none (add rows as needed)	Specifications/Comments (e.g., if payments were made to you or to your institution)
		Foundation (20220334), the Novonordisk Distinguished Investigator Grant - Endocrinology and Metabolism (NNF23OC0082114), the Novonordisk Project grants in Endocrinology and Metabolism (NNF24OC0091535) (SR).	
**Time frame: past 36 months**			
**2**	Grants or contracts from any entity (if not indicated in item #1 above).	X **None**	
**3**	Royalties or licenses	X **None**	
**4**	Consulting fees	X **None**	
**5**	Payment or honoraria for lectures, presentations, speakers bureaus, manuscript writing or educational events	X **None**	
**6**	Payment for expert testimony	x **None**	

		Name all entities with whom you have this relationship or indicate none (add rows as needed)	Specifications/Comments (e.g., if payments were made to you or to your institution)
7	Support for attending meetings and/or travel	x **None**	
8	Patents planned, issued or pending	x **None**	
9	Participation on a Data Safety Monitoring Board or Advisory Board	x **None**	
10	Leadership or fiduciary role in other board, society, committee or advocacy group, paid or unpaid	x **None**	
11	Stock or stock options	x **None**	
12	Receipt of equipment, materials, drugs, medical writing, gifts or other services	x **None**	
13	Other financial or non-financial interests	x **None**	

Please place an "X" next to the following statement to indicate your agreement:

x I certify that I have answered every question and have not altered the wording of any of the questions on this form.

ICMJE DISCLOSURE FORM

**Date:**	12/10/2025
**Your Name:**	Luisa Ronzoni
**Manuscript Title:**	***Carriage of rare APOB variants predisposes to severe steatotic liver disease and hepatocellular carcinoma***
**Manuscript Number (if known):**	201762-JCI-CRPH-1

In the interest of transparency, we ask you to disclose all relationships/activities/interests listed below that are related to the content of your manuscript. "Related" means any relation with for-profit or not-for-profit third parties whose interests may be affected by the content of the manuscript. Disclosure represents a commitment to transparency and does not necessarily indicate a bias. If you are in doubt about whether to list a relationship/activity/interest, it is preferable that you do so.

The author's relationships/activities/interests should be defined broadly. For example, if your manuscript pertains to the epidemiology of hypertension, you should declare all relationships with manufacturers of antihypertensive medication, even if that medication is not mentioned in the manuscript.

In item #1 below, report all support for the work reported in this manuscript without time limit. For all other items, the time frame for disclosure is the past 36 months.

		**Name all entities with whom you have this relationship or indicate none (add rows as needed)**	**Specifications/Comments (e.g., if payments were made to you or to your institution)**
**Time frame: Since the initial planning of the work**			
**1**	All support for the present manuscript (e.g., funding, provision of study materials, medical writing, article processing charges, etc.) **No time limit for this item.**	☐ **None**	
		NIH since MV received funding via R01DK134575 from the National Institute for Diabetes and Digestive and Kidney Diseases (MV); Italian Ministry of Health (Ministero della Salute), Ricerca Finalizzata 2021 RF-2021-12373889, Italian Ministry of Health, Ricerca Finalizzata PNRR 2022 "RATIONAL" PNRR-MAD-2022-12375656 (LV); Italian Ministry of Health (Ministero della Salute), Fondazione IRCCS Ca' Granda Ospedale Maggiore Policlinico, Ricerca Corrente (DP); The European Union, H2020-ICT-2018-20/H2020-ICT-2020-2 programme "Photonics" under grant agreement No. 101016726 - REVEAL (LV). The European Union, HORIZON-MISS-2021-CANCER-02-03 programme "Genial" under grant agreement "101096312" (LV, SP); Italian Ministry of Research (MUR) PNRR – M4 - C2 "National Center for Gene Therapy and Drugs based on RNA Technology" CN3, Spoke 4 "ASSET" (LV); PRIN 2022 MUR:	Funding provided to institution.

		Name all entities with whom you have this relationship or indicate none (add rows as needed)	Specifications/Comments (e.g., if payments were made to you or to your institution)
		"Disentangling genetic, epigenetic and hormonal regulation of Fe/heme metabolism in the gender-specific nature of NAFLD (DEFENDER)"; Bando Ricerca Corrente and Piano Nazionale Complementare Ecosistema Innovativo della Salute - Hub Life Science-Diagnostica Avanzata (HLS-DA)' - PNC-E3-2022-23683266 - 'INNOVA' (LV); Department of Pathophysiology and Transplantation, University of Milan, is funded by the Italian Ministry of Education and Research (MUR): Dipartimenti di Eccellenza Program 2023 to 2027) (LV); VA Merit Funding BX003362: "Genetics of Cardiometabolic Diseases in the VA Population" from the VA Office of R&D, Veterans Health Administration, USA (KMC, PST); The Swedish Cancerfonden (22 2270 Pj), the Swedish Research Council (Vetenskapsradet (VR), 2023-02079), the Swedish state under the Agreement between the Swedish government and the county councils (the ALF agreement, ALFGBG-965360), the Swedish Heart Lung Foundation (20220334), the Novonordisk Distinguished Investigator Grant - Endocrinology and Metabolism (NNF23OC0082114), the Novonordisk Project grants in Endocrinology and Metabolism (NNF24OC0091535) (SR).	

Time frame: past 36 months

**2**	Grants or contracts from any entity (if not indicated in item #1 above).	X **None**	
**3**	Royalties or licenses	X **None**	

		Name all entities with whom you have this relationship or indicate none (add rows as needed)	Specifications/Comments (e.g., if payments were made to you or to your institution)
4	Consulting fees	X None	
5	Payment or honoraria for lectures, presentations, speakers bureaus, manuscript writing or educational events	X None	
6	Payment for expert testimony	x None	
7	Support for attending meetings and/or travel	x None	
8	Patents planned, issued or pending	x None	
9	Participation on a Data Safety Monitoring Board or Advisory Board	x None	
10	Leadership or fiduciary role in other board, society, committee or advocacy group, paid or unpaid	x None	

		Name all entities with whom you have this relationship or indicate none (add rows as needed)	Specifications/Comments (e.g., if payments were made to you or to your institution)
11	Stock or stock options	x **None**	
12	Receipt of equipment, materials, drugs, medical writing, gifts or other services	x **None**	
13	Other financial or non-financial interests	x **None**	

Please place an "X" next to the following statement to indicate your agreement:

x I certify that I have answered every question and have not altered the wording of any of the questions on this form.

ICMJE DISCLOSURE FORM

Date: 12/10/2025

Your Name: Francesco Paolo Russo

Manuscript Title: ***Carriage of rare APOB variants predisposes to severe steatotic liver disease and hepatocellular carcinoma***

Manuscript Number (if known): 201762-JCI-CRPH-1

In the interest of transparency, we ask you to disclose all relationships/activities/interests listed below that are related to the content of your manuscript. "Related" means any relation with for-profit or not-for-profit third parties whose interests may be affected by the content of the manuscript. Disclosure represents a commitment to transparency and does not necessarily indicate a bias. If you are in doubt about whether to list a relationship/activity/interest, it is preferable that you do so.

The author's relationships/activities/interests should be defined broadly. For example, if your manuscript pertains to the epidemiology of hypertension, you should declare all relationships with manufacturers of antihypertensive medication, even if that medication is not mentioned in the manuscript.

In item #1 below, report all support for the work reported in this manuscript without time limit. For all other items, the time frame for disclosure is the past 36 months.

		Name all entities with whom you have this relationship or indicate none (add rows as needed)	Specifications/Comments (e.g., if payments were made to you or to your institution)
**Time frame: Since the initial planning of the work**			
**1**	All support for the present manuscript (e.g., funding, provision of study materials, medical writing, article processing charges, etc.) **No time limit for this item.**	☐ **None**	
		NIH since MV received funding via R01DK134575 from the National Institute for Diabetes and Digestive and Kidney Diseases (MV); Italian Ministry of Health (Ministero della Salute), Ricerca Finalizzata 2021 RF-2021-12373889, Italian Ministry of Health, Ricerca Finalizzata PNRR 2022 "RATIONAL" PNRR-MAD-2022-12375656 (LV); Italian Ministry of Health (Ministero della Salute), Fondazione IRCCS Ca' Granda Ospedale Maggiore Policlinico, Ricerca Corrente (DP); The European Union, H2020-ICT-2018-20/H2020-ICT-2020-2 programme "Photonics" under grant agreement No. 101016726 - REVEAL (LV). The European Union, HORIZON-MISS-2021-CANCER-02-03 programme "Genial" under grant agreement "101096312" (LV, SP); Italian Ministry of Research (MUR) PNRR – M4 - C2 "National Center for Gene Therapy and Drugs based on RNA Technology" CN3, Spoke 4 "ASSET" (LV); PRIN 2022 MUR: "Disentangling genetic, epigenetic and hormonal regulation of Fe/heme metabolism in the gender-specific nature of NAFLD (DEFENDER)"; Bando Ricerca Corrente and Piano Nazionale Complementare Ecosistema Innovativo della Salute - Hub Life Science-Diagnostica Avanzata (HLS-DA)' - PNC-E3-2022-23683266 - 'INNOVA' (LV); Department of Pathophysiology and Transplantation, University of Milan, is funded by the Italian Ministry of Education and Research (MUR): Dipartimenti di Eccellenza Program 2023 to 2027) (LV); VA Merit Funding BX003362: "Genetics of Cardiometabolic Diseases in the VA Population" from the VA Office of R&D, Veterans Health Administration, USA (KMC, PST); The Swedish Cancerfonden (22 2270 Pj), the Swedish Research Council (Vetenskapsradet (VR), 2023-02079), the Swedish state under the Agreement between the Swedish government and the county councils (the ALF agreement, ALFGBG-965360), the Swedish Heart Lung	Funding provided to institution.

		Name all entities with whom you have this relationship or indicate none (add rows as needed)	Specifications/Comments (e.g., if payments were made to you or to your institution)
		Foundation (20220334), the Novonordisk Distinguished Investigator Grant - Endocrinology and Metabolism (NNF23OC0082114), the Novonordisk Project grants in Endocrinology and Metabolism (NNF24OC0091535) (SR).	
**Time frame: past 36 months**			
**2**	Grants or contracts from any entity (if not indicated in item #1 above).	X **None**	
**3**	Royalties or licenses	X **None**	
**4**	Consulting fees	X **None**	
**5**	Payment or honoraria for lectures, presentations, speakers bureaus, manuscript writing or educational events	X **None**	
**6**	Payment for expert testimony	x **None**	

		Name all entities with whom you have this relationship or indicate none (add rows as needed)	Specifications/Comments (e.g., if payments were made to you or to your institution)
7	Support for attending meetings and/or travel	x **None**	
8	Patents planned, issued or pending	x **None**	
9	Participation on a Data Safety Monitoring Board or Advisory Board	x **None**	
10	Leadership or fiduciary role in other board, society, committee or advocacy group, paid or unpaid	x **None**	
11	Stock or stock options	x **None**	
12	Receipt of equipment, materials, drugs, medical writing, gifts or other services	x **None**	
13	Other financial or non-financial interests	x **None**	

Please place an "X" next to the following statement to indicate your agreement:

x I certify that I have answered every question and have not altered the wording of any of the questions on this form.

ICMJE DISCLOSURE FORM

**Date:**	12/10/2025
**Your Name:**	Francesco Malvestiti
**Manuscript Title:**	***Carriage of rare APOB variants predisposes to severe steatotic liver disease and hepatocellular carcinoma***
**Manuscript Number (if known):**	201762-JCI-CRPH-1

In the interest of transparency, we ask you to disclose all relationships/activities/interests listed below that are related to the content of your manuscript. "Related" means any relation with for-profit or not-for-profit third parties whose interests may be affected by the content of the manuscript. Disclosure represents a commitment to transparency and does not necessarily indicate a bias. If you are in doubt about whether to list a relationship/activity/interest, it is preferable that you do so.

The author's relationships/activities/interests should be defined broadly. For example, if your manuscript pertains to the epidemiology of hypertension, you should declare all relationships with manufacturers of antihypertensive medication, even if that medication is not mentioned in the manuscript.

In item #1 below, report all support for the work reported in this manuscript without time limit. For all other items, the time frame for disclosure is the past 36 months.

		**Name all entities with whom you have this relationship or indicate none (add rows as needed)**	**Specifications/Comments (e.g., if payments were made to you or to your institution)**
**Time frame: Since the initial planning of the work**			
**1**	All support for the present manuscript (e.g., funding, provision of study materials, medical writing, article processing charges, etc.) **No time limit for this item.**	☐ **None**	
		NIH since MV received funding via R01DK134575 from the National Institute for Diabetes and Digestive and Kidney Diseases (MV); Italian Ministry of Health (Ministero della Salute), Ricerca Finalizzata 2021 RF-2021-12373889, Italian Ministry of Health, Ricerca Finalizzata PNRR 2022 "RATIONAL" PNRR-MAD-2022-12375656 (LV); Italian Ministry of Health (Ministero della Salute), Fondazione IRCCS Ca' Granda Ospedale Maggiore Policlinico, Ricerca Corrente (DP); The European Union, H2020-ICT-2018-20/H2020-ICT-2020-2 programme "Photonics" under grant agreement No. 101016726 - REVEAL (LV). The European Union, HORIZON-MISS-2021-CANCER-02-03 programme "Genial" under grant agreement "101096312" (LV, SP); Italian Ministry of Research (MUR) PNRR – M4 - C2 "National Center for Gene Therapy and Drugs based on RNA Technology" CN3, Spoke 4 "ASSET" (LV); PRIN 2022 MUR:	Funding provided to institution.

		Name all entities with whom you have this relationship or indicate none (add rows as needed)	Specifications/Comments (e.g., if payments were made to you or to your institution)
		"Disentangling genetic, epigenetic and hormonal regulation of Fe/heme metabolism in the gender-specific nature of NAFLD (DEFENDER)"; Bando Ricerca Corrente and Piano Nazionale Complementare Ecosistema Innovativo della Salute - Hub Life Science-Diagnostica Avanzata (HLS-DA)' - PNC-E3-2022-23683266 - 'INNOVA' (LV); Department of Pathophysiology and Transplantation, University of Milan, is funded by the Italian Ministry of Education and Research (MUR): Dipartimenti di Eccellenza Program 2023 to 2027) (LV); VA Merit Funding BX003362: "Genetics of Cardiometabolic Diseases in the VA Population" from the VA Office of R&D, Veterans Health Administration, USA (KMC, PST); The Swedish Cancerfonden (22 2270 Pj), the Swedish Research Council (Vetenskapsradet (VR), 2023-02079), the Swedish state under the Agreement between the Swedish government and the county councils (the ALF agreement, ALFGBG-965360), the Swedish Heart Lung Foundation (20220334), the Novonordisk Distinguished Investigator Grant - Endocrinology and Metabolism (NNF23OC0082114), the Novonordisk Project grants in Endocrinology and Metabolism (NNF24OC0091535) (SR).	
**Time frame: past 36 months**			
**2**	Grants or contracts from any entity (if not indicated in item #1 above).	X **None**	
**3**	Royalties or licenses	X **None**	

		Name all entities with whom you have this relationship or indicate none (add rows as needed)	Specifications/Comments (e.g., if payments were made to you or to your institution)
4	Consulting fees	X **None**	
5	Payment or honoraria for lectures, presentations, speakers bureaus, manuscript writing or educational events	X **None**	
6	Payment for expert testimony	x **None**	
7	Support for attending meetings and/or travel	x **None**	
8	Patents planned, issued or pending	x **None**	
9	Participation on a Data Safety Monitoring Board or Advisory Board	x **None**	
10	Leadership or fiduciary role in other board, society, committee or advocacy group, paid or unpaid	x **None**	

		Name all entities with whom you have this relationship or indicate none (add rows as needed)	Specifications/Comments (e.g., if payments were made to you or to your institution)
11	Stock or stock options	x **None**	
12	Receipt of equipment, materials, drugs, medical writing, gifts or other services	x **None**	
13	Other financial or non-financial interests	x **None**	

Please place an "X" next to the following statement to indicate your agreement:

x I certify that I have answered every question and have not altered the wording of any of the questions on this form.

ICMJE DISCLOSURE FORM

Date: 12/10/2025

Your Name: Luca Miele

Manuscript Title: ***Carriage of rare APOB variants predisposes to severe steatotic liver disease and hepatocellular carcinoma***

Manuscript Number (if known): 201762-JCI-CRPH-1

In the interest of transparency, we ask you to disclose all relationships/activities/interests listed below that are related to the content of your manuscript. "Related" means any relation with for-profit or not-for-profit third parties whose interests may be affected by the content of the manuscript. Disclosure represents a commitment to transparency and does not necessarily indicate a bias. If you are in doubt about whether to list a relationship/activity/interest, it is preferable that you do so.

The author's relationships/activities/interests should be defined broadly. For example, if your manuscript pertains to the epidemiology of hypertension, you should declare all relationships with manufacturers of antihypertensive medication, even if that medication is not mentioned in the manuscript.

In item #1 below, report all support for the work reported in this manuscript without time limit. For all other items, the time frame for disclosure is the past 36 months.

		Name all entities with whom you have this relationship or indicate none (add rows as needed)	Specifications/Comments (e.g., if payments were made to you or to your institution)
**Time frame: Since the initial planning of the work**			
**1**	All support for the present manuscript (e.g., funding, provision of study materials, medical writing, article processing charges, etc.) **No time limit for this item.**	☐ **None**	
		NIH since MV received funding via R01DK134575 from the National Institute for Diabetes and Digestive and Kidney Diseases (MV); Italian Ministry of Health (Ministero della Salute), Ricerca Finalizzata 2021 RF-2021-12373889, Italian Ministry of Health, Ricerca Finalizzata PNRR 2022 "RATIONAL" PNRR-MAD-2022-12375656 (LV); Italian Ministry of Health (Ministero della Salute), Fondazione IRCCS Ca' Granda Ospedale Maggiore Policlinico, Ricerca Corrente (DP); The European Union, H2020-ICT-2018-20/H2020-ICT-2020-2 programme "Photonics" under grant agreement No. 101016726 - REVEAL (LV). The European Union, HORIZON-MISS-2021-CANCER-02-03 programme "Genial" under grant agreement "101096312" (LV, SP); Italian Ministry of Research (MUR) PNRR – M4 - C2 "National Center for Gene Therapy and Drugs based on RNA Technology" CN3, Spoke 4 "ASSET" (LV); PRIN 2022 MUR: "Disentangling genetic, epigenetic and hormonal regulation of Fe/heme metabolism in the gender-specific nature of NAFLD (DEFENDER)"; Bando Ricerca Corrente and Piano Nazionale Complementare Ecosistema Innovativo della Salute - Hub Life Science-Diagnostica Avanzata (HLS-DA)' - PNC-E3-2022-23683266 - 'INNOVA' (LV); Department of Pathophysiology and Transplantation, University of Milan, is funded by the Italian Ministry of Education and Research (MUR): Dipartimenti di Eccellenza Program 2023 to 2027) (LV); VA Merit Funding BX003362: "Genetics of Cardiometabolic Diseases in the VA Population" from the VA Office of R&D, Veterans Health Administration, USA (KMC, PST); The Swedish Cancerfonden (22 2270 Pj), the Swedish Research Council (Vetenskapsradet (VR), 2023-02079), the Swedish state under the Agreement between the Swedish government and the county councils (the ALF agreement, ALFGBG-965360), the Swedish Heart Lung	Funding provided to institution.

		Name all entities with whom you have this relationship or indicate none (add rows as needed)	Specifications/Comments (e.g., if payments were made to you or to your institution)
		Foundation (20220334), the Novonordisk Distinguished Investigator Grant - Endocrinology and Metabolism (NNF23OC0082114), the Novonordisk Project grants in Endocrinology and Metabolism (NNF24OC0091535) (SR).	
**Time frame: past 36 months**			
**2**	Grants or contracts from any entity (if not indicated in item #1 above).	X **None**	
**3**	Royalties or licenses	X **None**	
**4**	Consulting fees	X **None**	
**5**	Payment or honoraria for lectures, presentations, speakers bureaus, manuscript writing or educational events	X **None**	
**6**	Payment for expert testimony	x **None**	

		Name all entities with whom you have this relationship or indicate none (add rows as needed)	Specifications/Comments (e.g., if payments were made to you or to your institution)
7	Support for attending meetings and/or travel	x **None**	
8	Patents planned, issued or pending	x **None**	
9	Participation on a Data Safety Monitoring Board or Advisory Board	x **None**	
10	Leadership or fiduciary role in other board, society, committee or advocacy group, paid or unpaid	x **None**	
11	Stock or stock options	x **None**	
12	Receipt of equipment, materials, drugs, medical writing, gifts or other services	x **None**	
13	Other financial or non-financial interests	x **None**	

Please place an "X" next to the following statement to indicate your agreement:

x I certify that I have answered every question and have not altered the wording of any of the questions on this form.

ICMJE DISCLOSURE FORM

**Date:**	12/10/2025
**Your Name:**	Lorenzo Miano
**Manuscript Title:**	***Carriage of rare APOB variants predisposes to severe steatotic liver disease and hepatocellular carcinoma***
**Manuscript Number (if known):**	201762-JCI-CRPH-1

In the interest of transparency, we ask you to disclose all relationships/activities/interests listed below that are related to the content of your manuscript. "Related" means any relation with for-profit or not-for-profit third parties whose interests may be affected by the content of the manuscript. Disclosure represents a commitment to transparency and does not necessarily indicate a bias. If you are in doubt about whether to list a relationship/activity/interest, it is preferable that you do so.

The author's relationships/activities/interests should be defined broadly. For example, if your manuscript pertains to the epidemiology of hypertension, you should declare all relationships with manufacturers of antihypertensive medication, even if that medication is not mentioned in the manuscript.

In item #1 below, report all support for the work reported in this manuscript without time limit. For all other items, the time frame for disclosure is the past 36 months.

		**Name all entities with whom you have this relationship or indicate none (add rows as needed)**	**Specifications/Comments (e.g., if payments were made to you or to your institution)**
		**Time frame: Since the initial planning of the work**	
**1**	All support for the present manuscript (e.g., funding, provision of study materials, medical writing, article processing charges, etc.) **No time limit for this item.**	☐ **None**	
		NIH since MV received funding via R01DK134575 from the National Institute for Diabetes and Digestive and Kidney Diseases (MV); Italian Ministry of Health (Ministero della Salute), Ricerca Finalizzata 2021 RF-2021-12373889, Italian Ministry of Health, Ricerca Finalizzata PNRR 2022 "RATIONAL" PNRR-MAD-2022-12375656 (LV); Italian Ministry of Health (Ministero della Salute), Fondazione IRCCS Ca' Granda Ospedale Maggiore Policlinico, Ricerca Corrente (DP); The European Union, H2020-ICT-2018-20/H2020-ICT-2020-2 programme "Photonics" under grant agreement No. 101016726 - REVEAL (LV). The European Union, HORIZON-MISS-2021-CANCER-02-03 programme "Genial" under grant agreement "101096312" (LV, SP); Italian Ministry of Research (MUR) PNRR – M4 - C2 "National Center for Gene Therapy and Drugs based on RNA Technology" CN3, Spoke 4 "ASSET" (LV); PRIN 2022 MUR:	Funding provided to institution.

		Name all entities with whom you have this relationship or indicate none (add rows as needed)	Specifications/Comments (e.g., if payments were made to you or to your institution)
		"Disentangling genetic, epigenetic and hormonal regulation of Fe/heme metabolism in the gender-specific nature of NAFLD (DEFENDER)"; Bando Ricerca Corrente and Piano Nazionale Complementare Ecosistema Innovativo della Salute - Hub Life Science-Diagnostica Avanzata (HLS-DA)' - PNC-E3-2022-23683266 - 'INNOVA' (LV); Department of Pathophysiology and Transplantation, University of Milan, is funded by the Italian Ministry of Education and Research (MUR): Dipartimenti di Eccellenza Program 2023 to 2027) (LV); VA Merit Funding BX003362: "Genetics of Cardiometabolic Diseases in the VA Population" from the VA Office of R&D, Veterans Health Administration, USA (KMC, PST); The Swedish Cancerfonden (22 2270 Pj), the Swedish Research Council (Vetenskapsradet (VR), 2023-02079), the Swedish state under the Agreement between the Swedish government and the county councils (the ALF agreement, ALFGBG-965360), the Swedish Heart Lung Foundation (20220334), the Novonordisk Distinguished Investigator Grant - Endocrinology and Metabolism (NNF23OC0082114), the Novonordisk Project grants in Endocrinology and Metabolism (NNF24OC0091535) (SR).	

Time frame: past 36 months

2	Grants or contracts from any entity (if not indicated in item #1 above).	X **None**	
3	Royalties or licenses	X **None**	

		Name all entities with whom you have this relationship or indicate none (add rows as needed)	Specifications/Comments (e.g., if payments were made to you or to your institution)
4	Consulting fees	X None	
5	Payment or honoraria for lectures, presentations, speakers bureaus, manuscript writing or educational events	X None	
6	Payment for expert testimony	x None	
7	Support for attending meetings and/or travel	x None	
8	Patents planned, issued or pending	x None	
9	Participation on a Data Safety Monitoring Board or Advisory Board	x None	
10	Leadership or fiduciary role in other board, society, committee or advocacy group, paid or unpaid	x None	

		Name all entities with whom you have this relationship or indicate none (add rows as needed)	Specifications/Comments (e.g., if payments were made to you or to your institution)
11	Stock or stock options	x **None**	
12	Receipt of equipment, materials, drugs, medical writing, gifts or other services	x **None**	
13	Other financial or non-financial interests	x **None**	

Please place an "X" next to the following statement to indicate your agreement:

x I certify that I have answered every question and have not altered the wording of any of the questions on this form.

ICMJE DISCLOSURE FORM

Date: 12/10/2025

Your Name: Julie A Lynch

Manuscript Title: ***Carriage of rare APOB variants predisposes to severe steatotic liver disease and hepatocellular carcinoma***

Manuscript Number (if known): 201762-JCI-CRPH-1

In the interest of transparency, we ask you to disclose all relationships/activities/interests listed below that are related to the content of your manuscript. "Related" means any relation with for-profit or not-for-profit third parties whose interests may be affected by the content of the manuscript. Disclosure represents a commitment to transparency and does not necessarily indicate a bias. If you are in doubt about whether to list a relationship/activity/interest, it is preferable that you do so.

The author's relationships/activities/interests should be defined broadly. For example, if your manuscript pertains to the epidemiology of hypertension, you should declare all relationships with manufacturers of antihypertensive medication, even if that medication is not mentioned in the manuscript.

In item #1 below, report all support for the work reported in this manuscript without time limit. For all other items, the time frame for disclosure is the past 36 months.

		Name all entities with whom you have this relationship or indicate none (add rows as needed)	Specifications/Comments (e.g., if payments were made to you or to your institution)
**Time frame: Since the initial planning of the work**			
**1**	All support for the present manuscript (e.g., funding, provision of study materials, medical writing, article processing charges, etc.) **No time limit for this item.**	☐ **None**	
		NIH since MV received funding via R01DK134575 from the National Institute for Diabetes and Digestive and Kidney Diseases (MV); Italian Ministry of Health (Ministero della Salute), Ricerca Finalizzata 2021 RF-2021-12373889, Italian Ministry of Health, Ricerca Finalizzata PNRR 2022 "RATIONAL" PNRR-MAD-2022-12375656 (LV); Italian Ministry of Health (Ministero della Salute), Fondazione IRCCS Ca' Granda Ospedale Maggiore Policlinico, Ricerca Corrente (DP); The European Union, H2020-ICT-2018-20/H2020-ICT-2020-2 programme "Photonics" under grant agreement No. 101016726 - REVEAL (LV). The European Union, HORIZON-MISS-2021-CANCER-02-03 programme "Genial" under grant agreement "101096312" (LV, SP); Italian Ministry of Research (MUR) PNRR – M4 - C2 "National Center for Gene Therapy and Drugs based on RNA Technology" CN3, Spoke 4 "ASSET" (LV); PRIN 2022 MUR: "Disentangling genetic, epigenetic and hormonal regulation of Fe/heme metabolism in the gender-specific nature of NAFLD (DEFENDER)"; Bando Ricerca Corrente and Piano Nazionale Complementare Ecosistema Innovativo della Salute - Hub Life Science-Diagnostica Avanzata (HLS-DA)' - PNC-E3-2022-23683266 - 'INNOVA' (LV); Department of Pathophysiology and Transplantation, University of Milan, is funded by the Italian Ministry of Education and Research (MUR): Dipartimenti di Eccellenza Program 2023 to 2027) (LV); VA Merit Funding BX003362: "Genetics of Cardiometabolic Diseases in the VA Population" from the VA Office of R&D, Veterans Health Administration, USA (KMC, PST); The Swedish Cancerfonden (22 2270 Pj), the Swedish Research Council (Vetenskapsradet (VR), 2023-02079), the Swedish state under the Agreement between the Swedish government and the county councils (the ALF agreement, ALFGBG-965360), the Swedish Heart Lung	Funding provided to institution.

		Name all entities with whom you have this relationship or indicate none (add rows as needed)	Specifications/Comments (e.g., if payments were made to you or to your institution)
		Foundation (20220334), the Novonordisk Distinguished Investigator Grant - Endocrinology and Metabolism (NNF23OC0082114), the Novonordisk Project grants in Endocrinology and Metabolism (NNF24OC0091535) (SR).	
**Time frame: past 36 months**			
**2**	Grants or contracts from any entity (if not indicated in item #1 above).	X **None**	
**3**	Royalties or licenses	X **None**	
**4**	Consulting fees	X **None**	
**5**	Payment or honoraria for lectures, presentations, speakers bureaus, manuscript writing or educational events	X **None**	
**6**	Payment for expert testimony	x **None**	

		Name all entities with whom you have this relationship or indicate none (add rows as needed)	Specifications/Comments (e.g., if payments were made to you or to your institution)
**7**	Support for attending meetings and/or travel	x **None**	
**8**	Patents planned, issued or pending	x **None**	
**9**	Participation on a Data Safety Monitoring Board or Advisory Board	x **None**	
**10**	Leadership or fiduciary role in other board, society, committee or advocacy group, paid or unpaid	x **None**	
**11**	Stock or stock options	x **None**	
**12**	Receipt of equipment, materials, drugs, medical writing, gifts or other services	x **None**	
**13**	Other financial or non-financial interests	x **None**	

Please place an "X" next to the following statement to indicate your agreement:

x I certify that I have answered every question and have not altered the wording of any of the questions on this form.

ICMJE DISCLOSURE FORM

**Date:**	12/10/2025
**Your Name:**	Elisabetta Bugianesi
**Manuscript Title:**	***Carriage of rare APOB variants predisposes to severe steatotic liver disease and hepatocellular carcinoma***
**Manuscript Number (if known):**	201762-JCI-CRPH-1

In the interest of transparency, we ask you to disclose all relationships/activities/interests listed below that are related to the content of your manuscript. "Related" means any relation with for-profit or not-for-profit third parties whose interests may be affected by the content of the manuscript. Disclosure represents a commitment to transparency and does not necessarily indicate a bias. If you are in doubt about whether to list a relationship/activity/interest, it is preferable that you do so.

The author's relationships/activities/interests should be defined broadly. For example, if your manuscript pertains to the epidemiology of hypertension, you should declare all relationships with manufacturers of antihypertensive medication, even if that medication is not mentioned in the manuscript.

In item #1 below, report all support for the work reported in this manuscript without time limit. For all other items, the time frame for disclosure is the past 36 months.

		**Name all entities with whom you have this relationship or indicate none (add rows as needed)**	**Specifications/Comments (e.g., if payments were made to you or to your institution)**
**Time frame: Since the initial planning of the work**			
**1**	All support for the present manuscript (e.g., funding, provision of study materials, medical writing, article processing charges, etc.) **No time limit for this item.**	☐ **None**	
		NIH since MV received funding via R01DK134575 from the National Institute for Diabetes and Digestive and Kidney Diseases (MV); Italian Ministry of Health (Ministero della Salute), Ricerca Finalizzata 2021 RF-2021-12373889, Italian Ministry of Health, Ricerca Finalizzata PNRR 2022 "RATIONAL" PNRR-MAD-2022-12375656 (LV); Italian Ministry of Health (Ministero della Salute), Fondazione IRCCS Ca' Granda Ospedale Maggiore Policlinico, Ricerca Corrente (DP); The European Union, H2020-ICT-2018-20/H2020-ICT-2020-2 programme "Photonics" under grant agreement No. 101016726 - REVEAL (LV). The European Union, HORIZON-MISS-2021-CANCER-02-03 programme "Genial" under grant agreement "101096312" (LV, SP); Italian Ministry of Research (MUR) PNRR – M4 - C2 "National Center for Gene Therapy and Drugs based on RNA Technology" CN3, Spoke 4 "ASSET" (LV); PRIN 2022 MUR:	Funding provided to institution.

		Name all entities with whom you have this relationship or indicate none (add rows as needed)	Specifications/Comments (e.g., if payments were made to you or to your institution)
		"Disentangling genetic, epigenetic and hormonal regulation of Fe/heme metabolism in the gender-specific nature of NAFLD (DEFENDER)"; Bando Ricerca Corrente and Piano Nazionale Complementare Ecosistema Innovativo della Salute - Hub Life Science- Diagnostica Avanzata (HLS-DA)' - PNC-E3-2022-23683266 - 'INNOVA' (LV); Department of Pathophysiology and Transplantation, University of Milan, is funded by the Italian Ministry of Education and Research (MUR): Dipartimenti di Eccellenza Program 2023 to 2027) (LV); VA Merit Funding BX003362: "Genetics of Cardiometabolic Diseases in the VA Population" from the VA Office of R&D, Veterans Health Administration, USA (KMC, PST); The Swedish Cancerfonden (22 2270 Pj), the Swedish Research Council (Vetenskapsradet (VR), 2023-02079), the Swedish state under the Agreement between the Swedish government and the county councils (the ALF agreement, ALFGBG-965360), the Swedish Heart Lung Foundation (20220334), the Novonordisk Distinguished Investigator Grant - Endocrinology and Metabolism (NNF23OC0082114), the Novonordisk Project grants in Endocrinology and Metabolism (NNF24OC0091535) (SR).	

Time frame: past 36 months

**2**	Grants or contracts from any entity (if not indicated in item #1 above).	X **None**	
**3**	Royalties or licenses	X **None**	

		Name all entities with whom you have this relationship or indicate none (add rows as needed)	Specifications/Comments (e.g., if payments were made to you or to your institution)
**4**	Consulting fees	X **None**	
**5**	Payment or honoraria for lectures, presentations, speakers bureaus, manuscript writing or educational events	X **None**	
**6**	Payment for expert testimony	x **None**	
**7**	Support for attending meetings and/or travel	x **None**	
**8**	Patents planned, issued or pending	x **None**	
**9**	Participation on a Data Safety Monitoring Board or Advisory Board	x **None**	
**10**	Leadership or fiduciary role in other board, society, committee or advocacy group, paid or unpaid	x **None**	

		Name all entities with whom you have this relationship or indicate none (add rows as needed)	Specifications/Comments (e.g., if payments were made to you or to your institution)
11	Stock or stock options	x **None**	
12	Receipt of equipment, materials, drugs, medical writing, gifts or other services	x **None**	
13	Other financial or non-financial interests	x **None**	

Please place an "X" next to the following statement to indicate your agreement:

x I certify that I have answered every question and have not altered the wording of any of the questions on this form.

ICMJE DISCLOSURE FORM

Date: 12/10/2025

Your Name: Daniele Prati

Manuscript Title: ***Carriage of rare APOB variants predisposes to severe steatotic liver disease and hepatocellular carcinoma***

Manuscript Number (if known): 201762-JCI-CRPH-1

In the interest of transparency, we ask you to disclose all relationships/activities/interests listed below that are related to the content of your manuscript. "Related" means any relation with for-profit or not-for-profit third parties whose interests may be affected by the content of the manuscript. Disclosure represents a commitment to transparency and does not necessarily indicate a bias. If you are in doubt about whether to list a relationship/activity/interest, it is preferable that you do so.

The author's relationships/activities/interests should be defined broadly. For example, if your manuscript pertains to the epidemiology of hypertension, you should declare all relationships with manufacturers of antihypertensive medication, even if that medication is not mentioned in the manuscript.

In item #1 below, report all support for the work reported in this manuscript without time limit. For all other items, the time frame for disclosure is the past 36 months.

		Name all entities with whom you have this relationship or indicate none (add rows as needed)	Specifications/Comments (e.g., if payments were made to you or to your institution)
**Time frame: Since the initial planning of the work**			
**1**	All support for the present manuscript (e.g., funding, provision of study materials, medical writing, article processing charges, etc.) **No time limit for this item.**	☐ **None**	
		NIH since MV received funding via R01DK134575 from the National Institute for Diabetes and Digestive and Kidney Diseases (MV); Italian Ministry of Health (Ministero della Salute), Ricerca Finalizzata 2021 RF-2021-12373889, Italian Ministry of Health, Ricerca Finalizzata PNRR 2022 "RATIONAL" PNRR-MAD-2022-12375656 (LV); Italian Ministry of Health (Ministero della Salute), Fondazione IRCCS Ca' Granda Ospedale Maggiore Policlinico, Ricerca Corrente (DP); The European Union, H2020-ICT-2018-20/H2020-ICT-2020-2 programme "Photonics" under grant agreement No. 101016726 - REVEAL (LV). The European Union, HORIZON-MISS-2021-CANCER-02-03 programme "Genial" under grant agreement "101096312" (LV, SP); Italian Ministry of Research (MUR) PNRR – M4 - C2 "National Center for Gene Therapy and Drugs based on RNA Technology" CN3, Spoke 4 "ASSET" (LV); PRIN 2022 MUR: "Disentangling genetic, epigenetic and hormonal regulation of Fe/heme metabolism in the gender-specific nature of NAFLD (DEFENDER)"; Bando Ricerca Corrente and Piano Nazionale Complementare Ecosistema Innovativo della Salute - Hub Life Science-Diagnostica Avanzata (HLS-DA)' - PNC-E3-2022-23683266 - 'INNOVA' (LV); Department of Pathophysiology and Transplantation, University of Milan, is funded by the Italian Ministry of Education and Research (MUR): Dipartimenti di Eccellenza Program 2023 to 2027) (LV); VA Merit Funding BX003362: "Genetics of Cardiometabolic Diseases in the VA Population" from the VA Office of R&D, Veterans Health Administration, USA (KMC, PST); The Swedish Cancerfonden (22 2270 Pj), the Swedish Research Council (Vetenskapsradet (VR), 2023-02079), the Swedish state under the Agreement between the Swedish government and the county councils (the ALF agreement, ALFGBG-965360), the Swedish Heart Lung	Funding provided to institution.

		Name all entities with whom you have this relationship or indicate none (add rows as needed)	Specifications/Comments (e.g., if payments were made to you or to your institution)
		Foundation (20220334), the Novonordisk Distinguished Investigator Grant - Endocrinology and Metabolism (NNF23OC0082114), the Novonordisk Project grants in Endocrinology and Metabolism (NNF24OC0091535) (SR).	
**Time frame: past 36 months**			
**2**	Grants or contracts from any entity (if not indicated in item #1 above).	X **None**	
**3**	Royalties or licenses	X **None**	
**4**	Consulting fees	X **None**	
**5**	Payment or honoraria for lectures, presentations, speakers bureaus, manuscript writing or educational events	X **None**	
**6**	Payment for expert testimony	x **None**	

		Name all entities with whom you have this relationship or indicate none (add rows as needed)	Specifications/Comments (e.g., if payments were made to you or to your institution)
7	Support for attending meetings and/or travel	x **None**	
8	Patents planned, issued or pending	x **None**	
9	Participation on a Data Safety Monitoring Board or Advisory Board	x **None**	
10	Leadership or fiduciary role in other board, society, committee or advocacy group, paid or unpaid	x **None**	
11	Stock or stock options	x **None**	
12	Receipt of equipment, materials, drugs, medical writing, gifts or other services	x **None**	
13	Other financial or non-financial interests	x **None**	

Please place an "X" next to the following statement to indicate your agreement:

x I certify that I have answered every question and have not altered the wording of any of the questions on this form.

ICMJE DISCLOSURE FORM

**Date:**	12/10/2025
**Your Name:**	Craig Teerlink
**Manuscript Title:**	***Carriage of rare APOB variants predisposes to severe steatotic liver disease and hepatocellular carcinoma***
**Manuscript Number (if known):**	201762-JCI-CRPH-1

In the interest of transparency, we ask you to disclose all relationships/activities/interests listed below that are related to the content of your manuscript. "Related" means any relation with for-profit or not-for-profit third parties whose interests may be affected by the content of the manuscript. Disclosure represents a commitment to transparency and does not necessarily indicate a bias. If you are in doubt about whether to list a relationship/activity/interest, it is preferable that you do so.

The author's relationships/activities/interests should be defined broadly. For example, if your manuscript pertains to the epidemiology of hypertension, you should declare all relationships with manufacturers of antihypertensive medication, even if that medication is not mentioned in the manuscript.

In item #1 below, report all support for the work reported in this manuscript without time limit. For all other items, the time frame for disclosure is the past 36 months.

		**Name all entities with whom you have this relationship or indicate none (add rows as needed)**	**Specifications/Comments (e.g., if payments were made to you or to your institution)**
		**Time frame: Since the initial planning of the work**	
**1**	All support for the present manuscript (e.g., funding, provision of study materials, medical writing, article processing charges, etc.) **No time limit for this item.**	☐ **None**	
		NIH since MV received funding via R01DK134575 from the National Institute for Diabetes and Digestive and Kidney Diseases (MV); Italian Ministry of Health (Ministero della Salute), Ricerca Finalizzata 2021 RF-2021-12373889, Italian Ministry of Health, Ricerca Finalizzata PNRR 2022 "RATIONAL" PNRR-MAD-2022-12375656 (LV); Italian Ministry of Health (Ministero della Salute), Fondazione IRCCS Ca' Granda Ospedale Maggiore Policlinico, Ricerca Corrente (DP); The European Union, H2020-ICT-2018-20/H2020-ICT-2020-2 programme "Photonics" under grant agreement No. 101016726 - REVEAL (LV). The European Union, HORIZON-MISS-2021-CANCER-02-03 programme "Genial" under grant agreement "101096312" (LV, SP); Italian Ministry of Research (MUR) PNRR – M4 - C2 "National Center for Gene Therapy and	Funding provided to institution.

		Name all entities with whom you have this relationship or indicate none (add rows as needed)	Specifications/Comments (e.g., if payments were made to you or to your institution)
		Drugs based on RNA Technology" CN3, Spoke 4 "ASSET" (LV); PRIN 2022 MUR: "Disentangling genetic, epigenetic and hormonal regulation of Fe/heme metabolism in the gender-specific nature of NAFLD (DEFENDER)"; Bando Ricerca Corrente and Piano Nazionale Complementare Ecosistema Innovativo della Salute - Hub Life Science-Diagnostica Avanzata (HLS-DA)' - PNC-E3-2022-23683266 - 'INNOVA' (LV); Department of Pathophysiology and Transplantation, University of Milan, is funded by the Italian Ministry of Education and Research (MUR): Dipartimenti di Eccellenza Program 2023 to 2027) (LV); VA Merit Funding BX003362: "Genetics of Cardiometabolic Diseases in the VA Population" from the VA Office of R&D, Veterans Health Administration, USA (KMC, PST); The Swedish Cancerfonden (22 2270 Pj), the Swedish Research Council (Vetenskapsradet (VR), 2023-02079), the Swedish state under the Agreement between the Swedish government and the county councils (the ALF agreement, ALFGBG-965360), the Swedish Heart Lung Foundation (20220334), the Novonordisk Distinguished Investigator Grant - Endocrinology and Metabolism (NNF23OC0082114), the Novonordisk Project grants in Endocrinology and Metabolism (NNF24OC0091535) (SR).	
**Time frame: past 36 months**			
**2**	Grants or contracts from any entity (if not indicated in item #1 above).	X **None**	
**3**	Royalties or licenses	X **None**	

		Name all entities with whom you have this relationship or indicate none (add rows as needed)	Specifications/Comments (e.g., if payments were made to you or to your institution)
**4**	Consulting fees	X **None**	
**5**	Payment or honoraria for lectures, presentations, speakers bureaus, manuscript writing or educational events	X **None**	
**6**	Payment for expert testimony	x **None**	
**7**	Support for attending meetings and/or travel	x **None**	
**8**	Patents planned, issued or pending	x **None**	
**9**	Participation on a Data Safety Monitoring Board or Advisory Board	x **None**	
**10**	Leadership or fiduciary role in other board, society, committee or advocacy group, paid or unpaid	x **None**	

		Name all entities with whom you have this relationship or indicate none (add rows as needed)	Specifications/Comments (e.g., if payments were made to you or to your institution)
11	Stock or stock options	x **None**	
12	Receipt of equipment, materials, drugs, medical writing, gifts or other services	x **None**	
13	Other financial or non-financial interests	x **None**	

Please place an "X" next to the following statement to indicate your agreement:

x I certify that I have answered every question and have not altered the wording of any of the questions on this form.

ICMJE DISCLOSURE FORM

**Date:**	12/10/2025
**Your Name:**	Carolin V Schneider
**Manuscript Title:**	***Carriage of rare APOB variants predisposes to severe steatotic liver disease and hepatocellular carcinoma***
**Manuscript Number (if known):**	201762-JCI-CRPH-1

In the interest of transparency, we ask you to disclose all relationships/activities/interests listed below that are related to the content of your manuscript. "Related" means any relation with for-profit or not-for-profit third parties whose interests may be affected by the content of the manuscript. Disclosure represents a commitment to transparency and does not necessarily indicate a bias. If you are in doubt about whether to list a relationship/activity/interest, it is preferable that you do so.

The author's relationships/activities/interests should be defined broadly. For example, if your manuscript pertains to the epidemiology of hypertension, you should declare all relationships with manufacturers of antihypertensive medication, even if that medication is not mentioned in the manuscript.

In item #1 below, report all support for the work reported in this manuscript without time limit. For all other items, the time frame for disclosure is the past 36 months.

		Name all entities with whom you have this relationship or indicate none (add rows as needed)	Specifications/Comments (e.g., if payments were made to you or to your institution)
**Time frame: Since the initial planning of the work**			
**1**	All support for the present manuscript (e.g., funding, provision of study materials, medical writing, article processing charges, etc.)<br>**No time limit for this item.**	☐ **None**<br><br>NIH since MV received funding via R01DK134575 from the National Institute for Diabetes and Digestive and Kidney Diseases (MV); Italian Ministry of Health (Ministero della Salute), Ricerca Finalizzata 2021 RF-2021-12373889, Italian Ministry of Health, Ricerca Finalizzata PNRR 2022 "RATIONAL" PNRR-MAD-2022-12375656 (LV); Italian Ministry of Health (Ministero della Salute), Fondazione IRCCS Ca' Granda Ospedale Maggiore Policlinico, Ricerca Corrente (DP); The European Union, H2020-ICT-2018-20/H2020-ICT-2020-2 programme "Photonics" under grant agreement No. 101016726 - REVEAL (LV). The European Union, HORIZON-MISS-2021-CANCER-02-03 programme "Genial" under grant agreement "101096312" (LV, SP); Italian Ministry of Research (MUR) PNRR – M4 - C2 "National Center for Gene Therapy and Drugs based on RNA Technology" CN3, Spoke 4 "ASSET" (LV); PRIN 2022 MUR: "Disentangling genetic, epigenetic and hormonal regulation of Fe/heme metabolism in the gender-specific nature of NAFLD (DEFENDER)"; Bando Ricerca Corrente and Piano Nazionale Complementare Ecosistema Innovativo della Salute - Hub Life Science-Diagnostica Avanzata (HLS-DA)' - PNC-E3-2022-23683266 - 'INNOVA' (LV); Department of Pathophysiology and Transplantation, University of Milan, is funded by the Italian Ministry of Education and Research (MUR): Dipartimenti di Eccellenza Program 2023 to 2027) (LV); VA Merit Funding BX003362: "Genetics of Cardiometabolic Diseases in the VA Population" from the VA Office of R&D, Veterans Health Administration, USA (KMC, PST); The Swedish Cancerfonden (22 2270 Pj), the Swedish Research Council (Vetenskapsradet (VR), 2023-02079), the Swedish state under the Agreement between the Swedish government and the county councils (the ALF agreement, ALFGBG-965360), the Swedish Heart Lung	Funding provided to institution.

		Name all entities with whom you have this relationship or indicate none (add rows as needed)	Specifications/Comments (e.g., if payments were made to you or to your institution)
		Foundation (20220334), the Novonordisk Distinguished Investigator Grant - Endocrinology and Metabolism (NNF23OC0082114), the Novonordisk Project grants in Endocrinology and Metabolism (NNF24OC0091535) (SR).	
**Time frame: past 36 months**			
**2**	Grants or contracts from any entity (if not indicated in item #1 above).	X **None**	
**3**	Royalties or licenses	X **None**	
**4**	Consulting fees	X **None**	
**5**	Payment or honoraria for lectures, presentations, speakers bureaus, manuscript writing or educational events	X **None**	
**6**	Payment for expert testimony	x **None**	

		Name all entities with whom you have this relationship or indicate none (add rows as needed)	Specifications/Comments (e.g., if payments were made to you or to your institution)
7	Support for attending meetings and/or travel	x **None**	
8	Patents planned, issued or pending	x **None**	
9	Participation on a Data Safety Monitoring Board or Advisory Board	x **None**	
10	Leadership or fiduciary role in other board, society, committee or advocacy group, paid or unpaid	x **None**	
11	Stock or stock options	x **None**	
12	Receipt of equipment, materials, drugs, medical writing, gifts or other services	x **None**	
13	Other financial or non-financial interests	x **None**	

Please place an "X" next to the following statement to indicate your agreement:

x I certify that I have answered every question and have not altered the wording of any of the questions on this form.

ICMJE DISCLOSURE FORM

**Date:**	12/10/2025
**Your Name:**	Alessandro Cherubini
**Manuscript Title:**	***Carriage of rare APOB variants predisposes to severe steatotic liver disease and hepatocellular carcinoma***
**Manuscript Number (if known):**	201762-JCI-CRPH-1

In the interest of transparency, we ask you to disclose all relationships/activities/interests listed below that are related to the content of your manuscript. "Related" means any relation with for-profit or not-for-profit third parties whose interests may be affected by the content of the manuscript. Disclosure represents a commitment to transparency and does not necessarily indicate a bias. If you are in doubt about whether to list a relationship/activity/interest, it is preferable that you do so.

The author's relationships/activities/interests should be defined broadly. For example, if your manuscript pertains to the epidemiology of hypertension, you should declare all relationships with manufacturers of antihypertensive medication, even if that medication is not mentioned in the manuscript.

In item #1 below, report all support for the work reported in this manuscript without time limit. For all other items, the time frame for disclosure is the past 36 months.

		**Name all entities with whom you have this relationship or indicate none (add rows as needed)**	**Specifications/Comments (e.g., if payments were made to you or to your institution)**
**Time frame: Since the initial planning of the work**			
**1**	All support for the present manuscript (e.g., funding, provision of study materials, medical writing, article processing charges, etc.) **No time limit for this item.**	☐ **None**	
		NIH since MV received funding via R01DK134575 from the National Institute for Diabetes and Digestive and Kidney Diseases (MV); Italian Ministry of Health (Ministero della Salute), Ricerca Finalizzata 2021 RF-2021-12373889, Italian Ministry of Health, Ricerca Finalizzata PNRR 2022 "RATIONAL" PNRR-MAD-2022-12375656 (LV); Italian Ministry of Health (Ministero della Salute), Fondazione IRCCS Ca' Granda Ospedale Maggiore Policlinico, Ricerca Corrente (DP); The European Union, H2020-ICT-2018-20/H2020-ICT-2020-2 programme "Photonics" under grant agreement No. 101016726 - REVEAL (LV). The European Union, HORIZON-MISS-2021-CANCER-02-03 programme "Genial" under grant agreement "101096312" (LV, SP); Italian Ministry of Research (MUR) PNRR – M4 - C2 "National Center for Gene Therapy and	Funding provided to institution.

		Name all entities with whom you have this relationship or indicate none (add rows as needed)	Specifications/Comments (e.g., if payments were made to you or to your institution)
		Drugs based on RNA Technology” CN3, Spoke 4 “ASSET” (LV); PRIN 2022 MUR: “Disentangling genetic, epigenetic and hormonal regulation of Fe/heme metabolism in the gender-specific nature of NAFLD (DEFENDER)”; Bando Ricerca Corrente and Piano Nazionale Complementare Ecosistema Innovativo della Salute - Hub Life Science-Diagnostica Avanzata (HLS-DA)’ - PNC-E3-2022-23683266 - ‘INNOVA’ (LV); Department of Pathophysiology and Transplantation, University of Milan, is funded by the Italian Ministry of Education and Research (MUR): Dipartimenti di Eccellenza Program 2023 to 2027) (LV); VA Merit Funding BX003362: “Genetics of Cardiometabolic Diseases in the VA Population” from the VA Office of R&D, Veterans Health Administration, USA (KMC, PST); The Swedish Cancerfonden (22 2270 Pj), the Swedish Research Council (Vetenskapsradet (VR), 2023-02079), the Swedish state under the Agreement between the Swedish government and the county councils (the ALF agreement, ALFGBG-965360), the Swedish Heart Lung Foundation (20220334), the Novonordisk Distinguished Investigator Grant - Endocrinology and Metabolism (NNF23OC0082114), the Novonordisk Project grants in Endocrinology and Metabolism (NNF24OC0091535) (SR).	

Time frame: past 36 months

**2**	Grants or contracts from any entity (if not indicated in item #1 above).	X **None**	
**3**	Royalties or licenses	X **None**	

		Name all entities with whom you have this relationship or indicate none (add rows as needed)	Specifications/Comments (e.g., if payments were made to you or to your institution)
4	Consulting fees	X **None**	
5	Payment or honoraria for lectures, presentations, speakers bureaus, manuscript writing or educational events	X **None**	
6	Payment for expert testimony	x **None**	
7	Support for attending meetings and/or travel	x **None**	
8	Patents planned, issued or pending	x **None**	
9	Participation on a Data Safety Monitoring Board or Advisory Board	x **None**	
10	Leadership or fiduciary role in other board, society, committee or advocacy group, paid or unpaid	x **None**	

		Name all entities with whom you have this relationship or indicate none (add rows as needed)	Specifications/Comments (e.g., if payments were made to you or to your institution)
11	Stock or stock options	x **None**	
12	Receipt of equipment, materials, drugs, medical writing, gifts or other services	x **None**	
13	Other financial or non-financial interests	x **None**	

Please place an "X" next to the following statement to indicate your agreement:

x I certify that I have answered every question and have not altered the wording of any of the questions on this form.

ICMJE DISCLOSURE FORM

Date: 12/10/2025

Your Name: Kyong-Mi Chang

Manuscript Title: ***Carriage of rare APOB variants predisposes to severe steatotic liver disease and hepatocellular carcinoma***

Manuscript Number (if known): 201762-JCI-CRPH-1

In the interest of transparency, we ask you to disclose all relationships/activities/interests listed below that are related to the content of your manuscript. "Related" means any relation with for-profit or not-for-profit third parties whose interests may be affected by the content of the manuscript. Disclosure represents a commitment to transparency and does not necessarily indicate a bias. If you are in doubt about whether to list a relationship/activity/interest, it is preferable that you do so.

The author's relationships/activities/interests should be defined broadly. For example, if your manuscript pertains to the epidemiology of hypertension, you should declare all relationships with manufacturers of antihypertensive medication, even if that medication is not mentioned in the manuscript.

In item #1 below, report all support for the work reported in this manuscript without time limit. For all other items, the time frame for disclosure is the past 36 months.

		Name all entities with whom you have this relationship or indicate none (add rows as needed)	Specifications/Comments (e.g., if payments were made to you or to your institution)
**Time frame: Since the initial planning of the work**			
**1**	All support for the present manuscript (e.g., funding, provision of study materials, medical writing, article processing charges, etc.) **No time limit for this item.**	☐ **None**	
		NIH since MV received funding via R01DK134575 from the National Institute for Diabetes and Digestive and Kidney Diseases (MV); Italian Ministry of Health (Ministero della Salute), Ricerca Finalizzata 2021 RF-2021-12373889, Italian Ministry of Health, Ricerca Finalizzata PNRR 2022 “RATIONAL” PNRR-MAD-2022-12375656 (LV); Italian Ministry of Health (Ministero della Salute), Fondazione IRCCS Ca’ Granda Ospedale Maggiore Policlinico, Ricerca Corrente (DP); The European Union, H2020-ICT-2018-20/H2020-ICT-2020-2 programme “Photonics” under grant agreement No. 101016726 - REVEAL (LV). The European Union, HORIZON-MISS-2021-CANCER-02-03 programme “Genial” under grant agreement “101096312” (LV, SP); Italian Ministry of Research (MUR) PNRR – M4 - C2 “National Center for Gene Therapy and Drugs based on RNA Technology” CN3, Spoke 4 “ASSET” (LV); PRIN 2022 MUR: “Disentangling genetic, epigenetic and hormonal regulation of Fe/heme metabolism in the gender-specific nature of NAFLD (DEFENDER)”; Bando Ricerca Corrente and Piano Nazionale Complementare Ecosistema Innovativo della Salute - Hub Life Science-Diagnostica Avanzata (HLS-DA)’ - PNC-E3-2022-23683266 - ‘INNOVA’ (LV); Department of Pathophysiology and Transplantation, University of Milan, is funded by the Italian Ministry of Education and Research (MUR): Dipartimenti di Eccellenza Program 2023 to 2027) (LV); VA Merit Funding BX003362: “Genetics of Cardiometabolic Diseases in the VA Population” from the VA Office of R&D, Veterans Health Administration, USA (KMC, PST); The Swedish Cancerfonden (22 2270 Pj), the Swedish Research Council (Vetenskapsradet (VR), 2023-02079), the Swedish state under the Agreement between the Swedish government and the county councils (the ALF agreement, ALFGBG-965360), the Swedish Heart Lung	Funding provided to institution.

		Name all entities with whom you have this relationship or indicate none (add rows as needed)	Specifications/Comments (e.g., if payments were made to you or to your institution)
		Foundation (20220334), the Novonordisk Distinguished Investigator Grant - Endocrinology and Metabolism (NNF23OC0082114), the Novonordisk Project grants in Endocrinology and Metabolism (NNF24OC0091535) (SR).	
**Time frame: past 36 months**			
**2**	Grants or contracts from any entity (if not indicated in item #1 above).	X **None**	
**3**	Royalties or licenses	X **None**	
**4**	Consulting fees	X **None**	
**5**	Payment or honoraria for lectures, presentations, speakers bureaus, manuscript writing or educational events	X **None**	
**6**	Payment for expert testimony	x **None**	

		Name all entities with whom you have this relationship or indicate none (add rows as needed)	Specifications/Comments (e.g., if payments were made to you or to your institution)
7	Support for attending meetings and/or travel	x **None**	
8	Patents planned, issued or pending	x **None**	
9	Participation on a Data Safety Monitoring Board or Advisory Board	x **None**	
10	Leadership or fiduciary role in other board, society, committee or advocacy group, paid or unpaid	x **None**	
11	Stock or stock options	x **None**	
12	Receipt of equipment, materials, drugs, medical writing, gifts or other services	x **None**	
13	Other financial or non-financial interests	x **None**	

Please place an "X" next to the following statement to indicate your agreement:

x I certify that I have answered every question and have not altered the wording of any of the questions on this form.
